# Phylogeography and DNA-based species delimitation provide insight into the taxonomy of the polymorphic rose chafer *Protaetia* (*Potosia*) *cuprea* species complex (Coleoptera: Scarabaeidae: Cetoniinae) in the Western Palearctic

**DOI:** 10.1371/journal.pone.0192349

**Published:** 2018-02-20

**Authors:** Dominik Vondráček, Aneta Fuchsová, Dirk Ahrens, David Král, Petr Šípek

**Affiliations:** 1 Department of Zoology, Faculty of Science, Charles University, Prague, Czech Republic; 2 Department of Arthropoda, Zoologisches Forschungsmuseum Alexander Koenig, Bonn, Germany; Sichuan University, CHINA

## Abstract

The development of modern methods of species delimitation, unified under the “integrated taxonomy” approach, allows a critical examination and re-evaluation of complex taxonomic groups. The rose chafer *Protaetia* (*Potosia*) *cuprea* is a highly polymorphic species group with a large distribution range. Despite its overall commonness, its taxonomy is unclear and subject to conflicting hypotheses, most of which largely fail to account for its evolutionary history. Based on the sequences of two mitochondrial markers from 65 individuals collected across the species range, and a detailed analysis of morphological characters including a geometric morphometry approach, we infer the evolutionary history and phylogeography of the *P*. *cuprea* species complex. Our results demonstrate the existence of three separate lineages in the Western Palearctic region, presumably with a species status. However, these lineages are in conflict with current taxonomic concepts. None of the 29 analyzed morphological characters commonly used in the taxonomy of this group proved to be unambiguously species- or subspecies- specific. The geometric morphometry analysis reveals a large overlap in the shape of the analyzed structures (pronotum, meso-metaventral projection, elytra and aedeagus), failing to identify either the genetically detected clades or the classical species entities. Our results question the monophyly of *P*. *cuprea* in regard to *P*. *cuprina*, as well as the species status of *P*. *metallica*. On the other hand, we found support for the species status of the Sicilian *P*. *hypocrita*. Collectively, our findings provide a new and original insight into the taxonomy and phylogeny of the *P*. *cuprea* species complex. At the same time, the results represent the first attempt to elucidate the phylogeography of these polymorphic beetles.

## Introduction

It has been repeatedly demonstrated that DNA sequences provide a powerful tool for the recognition of phylogenetic patterns within a species complex (e.g. [1–4]) which is essential for the reconstruction of their range evolution (phylogeography) and for defining potential conservation units [5]. Propelled by an easy DNA data acquisition, this approach has led to a renaissance of its use for taxonomic hypothesis testing in a number of insect species (e.g. [6–10]). This resulted in global campaigns to build reference libraries for such data as in the Barcoding of Life initiative ([11] http://www.ibol.org/). Although DNA barcoding is a promising approach for easy and fast taxon identification, its results may not be sufficient for a total resolution of complicated taxonomic questions (e.g. [12–15]). Therefore, the barcode is increasingly regarded only as a partial data source and a more complex approach combining various molecular results with classical taxonomical tools (i.e. morphological analyses), geometric morphometry, phylogeographic data, as well as information on ecology and biology united under the “integrated taxonomy” approach [16] has proven to better address critical taxonomic groups. Such a combined approach provided novel insight even in such notoriously known taxa as the Hercules beetle *Dynastes hercules* (Linnaeus, 1758) species complex [17].

Rose chafers (Coleoptera: Scarabaeidae: Cetoniinae) are arguably some of the showiest and most popular beetles. However, the overall state of knowledge in this group is rather poor, due to a general ignorance by professional taxonomists as well as by the number of inappropriate and rather confusing taxonomic works published in journals without peer review (e.g. [18–20]). The rose chafer *Protaetia* (subgenus *Potosia*) *cuprea* (Fabricius, 1775), hereafter abbreviated as *P*. *cuprea*, is among the most charismatic beetle species in Europe. It has a broad range extending from Canary Islands, Portugal and Spain in the west towards Vladivostok in the Russian Far East, Mongolia, and northern China [21–23]. In the Middle East, the species is present in Turkey, the Levant, northern Egypt, and Iran, but it also occurs in Pakistan, and Nepal [21, 24, 25]. Hand in hand with its vast geographical distribution, *P*. *cuprea* shows an exceptionally wide range of ecological preferences. It occurs in forests as well as steppe habitats, and from the shoreline up to elevations of 2000 meters [21, 26, 27]. The species usually has a one-year life cycle, but rarely the development may be completed more quickly and adults emerge in the same year that the eggs were laid by parental generation [28]. Larvae are considered primarily saproxylophagous [21] with a strong affinity to deciduous trees (especially oaks–*Quercus* spp.). However, transitions to pure saprophagy (e.g. development in compost heaps [29] or association with ant-colonies (e.g. [30, 26, 31]) are also frequently reported.

Arguably one of the most widely distributed flower chafer species of the Palearctic region, this taxon comprises several morphologically distinct forms, frequently referred to as subspecies or species. Contrasting to its high morphological variability, the chromosome number of the species is 2n = 20 as reported for other representatives of the genus *Protaetia*. Still, minor differences in X-chromosome morphology are reported between some subspecies [32, 33]. A number of opposing taxonomies have been proposed by different authors based on its wide geographical range, variation in coloration, body size, macrosetation, and punctation ([34, 21, 23, 35] see S1 Table for an overview). The taxonomic confusion is extended with the ongoing publications of new descriptions of sub-specific taxa based solely on coloration patterns, body size and geographical distribution without proper taxonomic analysis or comparison with the type material [36].

To investigate the relevance of the applied classifications (see S1 Table) we examined representatives of 11 out of 16 currently recognized and relevant subspecies of *P*. *cuprea* and closely allied *P*. *cuprina* (Motschulsky, 1849) and *P*. *hypocrita* (Ragusa, 1905) [21] from Europe and adjacent regions (excluding Caucasian area). The latter two taxa, recently ranked as separate species [25], were historically classified as subspecies of *P*. *cuprea* (S1 Table). We evaluated the population divergence of the species complex using two mitochondrial DNA markers along with an analysis of morphology, coloration patterns and geographical distribution of identified clades. This type of integrated approach has proven to be useful for understanding species variability and genetic diversity in related taxa (e.g. [13]). Additionally, we tested (i) the alleged species status of *P*. *cuprea metallica* (Herbst, 1782), (ii) the status of the Sicilian *P*. *hypocrita*, (iii) the validity of subspecies described in the *P*. *cuprea* complex, and (iv) evaluated the relationship between *P*. *cuprea* and *P*. *cuprina* in the light of our current taxonomic hypothesis.

## Material and methods

### Taxon sampling and acquiring DNA sequences

**Fig 1:**
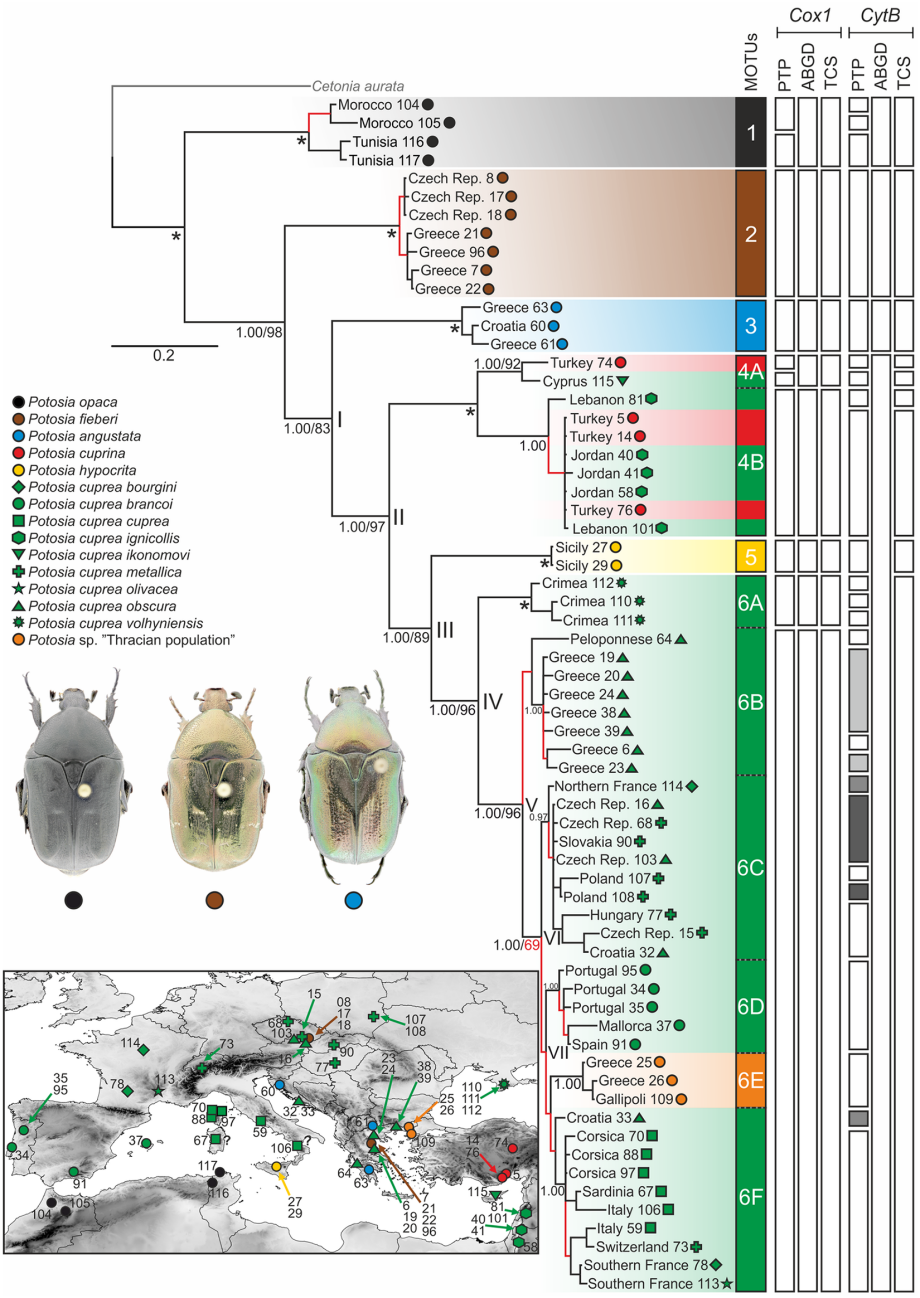
Majority-rule consensus tree from Bayesian analysis (BI) based on concatenate of *Cox1* and *CytB* fragments. Posterior probabilities (PP; left number) and bootstraps from maximum likelihood (ML) analysis (BS; right number) are provided for main splits and clades with identical topology in the two inference methods. Asterisks mark a fully supported branch in both analyses. In nodes with conflict topology (BI vs. ML) only PP values are provided. Red colored branches indicate low support value (PP < 0.95, BS < 75). Main splits are marked with Roman numbers (I—VII). Color symbols and shadowing reflects the species identity according to the current taxonomic opinion, see legend on the left side of the figure. Identified clades (MOTUs) are marked with Arabic numbers on the colored sidebar next to the tree. The other six sidebars represent the results of PTP, ABGD and TCS analyses for each gene. A map with localities of all vouchers is attached in bottom left corner of the figure (“?” = exact voucher locality in specific region is not available).

DNA was extracted from 65 individuals of *Potosia* spp. (S2 Table) from 47 localities (map in Fig 1) across Europe, North Africa, the Levant and Asian Turkey. Morphospecies identification was based on keys of Mikšić [21] and Baraud [23, 37]. The study included samples of nine *P*. *cuprea* subspecies (namely *P*. *c*. *bourgini* Ruter, 1967; *P*. *c*. *brancoi* Baraud, 1992; *P*. *c*. *cuprea* (Fabricius, 1775); *P*. *c*. *ignicollis* (Gory & Percheron, 1833); *P*. *c*. *ikonomovi* Mikšić, 1958; *P*. *c*. *metallica* (Herbst, 1782); *P*. *c*. *obscura* (Andersch, 1797); *P*. *c*. *olivacea* (Mulsant, 1842); *P*. *c*. *volhyniensis* (Gory & Percheron, 1833)). Three individuals from Greece and Turkey, which could not have been assigned to any valid taxon according to the current literature, were provisionally labeled as *Potosia* sp. “Thracian population”. Furthermore, the closely related *P*. *cuprina* and *P*. *hypocrita* [21] were added to cover the “*cuprea* species complex”, while *P*. *angustata* (Germar, 1817), *P*. *fieberi* (Kraatz, 1880), and *P*. *opaca* (Fabricius, 1787) were included to obtain a realistic view of the genetic variability within the subgenus *Potosia*. We used *Cetonia aurata* (Linnaeus, 1758) to root the resulting trees. Nomenclature was adopted from Bezděk [25].

Specimens for DNA extraction were stored in 96% ethanol immediately after the capture. However, a few dry samples were also included in the study. Genomic DNA from thoracic leg muscle tissue was extracted non-destructively using a Qiagen Blood and Tissue Kit, following standard protocols. After DNA extraction, beetles and male genitalia were dry mounted. Vouchers were deposited at the Charles University in Prague (collection of PS and DV). Partial sequences of two mitochondrial protein coding genes *cytochrome oxidase subunit 1* (C*ox1*) and *cytochrome b* (*CytB*) were used in the study. For amplification, primers stev_jerryF (5′-CAACATYTATTYTGATTYTTTGG-3′) and stev_patR (5′-GCACTAWTCTGCCATATTAGA-3′) were used for *Cox1* [38], and CB3 (5′-GAGGAGCAACTGTAATTACTAA-3′) and CB4 (5′-AAAAGAAARTATCATTCAGGTTGAAT-3′) for *CytB* [39]. The PCR conditions used are as follows for *Cox1*: initialization at 95°C for 5 min, denaturation at 95°C for 30 s, annealing at 50°C for 40 s, elongation at 72°C for 2 min (last three steps for 40 cycles), final elongation at 72°C for 10 min. For *CytB*: initialization at 95°C for 5 min, denaturation at 94°C for 1 min, annealing at 47°C for 1 min, elongation at 72°C for 90 s (last three steps for 40 cycles), final elongation at 72°C for 10 min. Sequencing was done using sequencer 3130 and 3130xl Genetic Analyzer (Applied Biosystems) with BigDye^®^Terminator v3.1 Cycle Sequencing Kit (Applied Biosystems). Bidirectional sequences were aligned to form contigs and edited using Geneious 9.1 ([40] http://www.geneious.com). Sequences were submitted to the GenBank (NCBI) under accession numbers in S2 Table.

### Phylogenetic analysis, molecular dating and genetic distances

Sequences were edited and aligned using the Geneious inbuilt algorithm. The best-fit models of molecular evolution for the aligned datasets were chosen using PartitionFinder v1.1.1 [41] with Bayesian information criterion (BIC) and the “greedy” algorithm. Phylogenetic trees were constructed under Bayesian inference (BI) with MrBayes 3.2.5 [42], using eight chains (in two runs) of 50*10^6^ generations, sampling the chains every 500 generations for a concatenate of both genes. For separate genes, we used 30*10^6^ (*Cox1*) and 20*10^6^ generations (*CytB*). Stationarity in MCM chains was determined using Tracer 1.6 [43] and burn-in was set accordingly. Maximum likelihood (ML) analysis was conducted for all three datasets using raxmlGUI 1.5 [44] running the ML+thorough bootstrap analysis with 1 run of 1000 replicates and appropriate substitution models.

Molecular dating was done in BEAST v.2.4.6 [45] using only the *Cox1* gene under strict clock with the appropriate nucleotide substitution models for each codon position. Due to a lack of fossil data or reliable geological events, the divergence time was based on previously published rates [46]. A rate of 0.0177 for *Cox1* was used for three separate runs with a Yule tree prior which lasted for 10^9^ generations. Stationarity in MCM chains was determined using Tracer 1.6 and the log files were later pooled using LogCombiner [45] with the suitable burn-in. A maximum clade credibility tree with means of node heights was constructed in TreeAnnotator [45].

Genetic distances were calculated for the concatenate using Kimura 2-parameter model [47] as implemented in MEGA6.06 software [48] with estimated standard errors using 1000 bootstrap replicate procedure.

### Network computation and species delimitation

A neighbor-joining (NJ) network as implemented in SplitsTree4 v4.12.3 [49] was used to represent incompatible and unclear signals in the concatenated dataset. In such a network, parallel edges, rather than single branches, are used to represent the splits calculated from the data. To accommodate incompatible splits, a split network may, and often does, contain nodes that do not represent ancestral species. Because of this, the representation of evolutionary history provided by the network is only ‘‘implicit” [49]. Parsimony networks were constructed for all three datasets via TCS 1.21 [50] with a connection limit of 95% (calculation for DNA pairwise differences until the probability exceeds 0.95) for visualization of possible phylogeographic patterns. For insect mtDNA this analysis split the haplotypes into subgroups (separated networks) and usually joins haplotypes above the species-level [51–53].

Poisson Tree Processes (PTP) and Automatic Barcode Gap Discovery (ABGD) were used to delimitate species for separate gene datasets. In PTP, the Yule-coalescent transition points that mark species boundaries are modeled based on the change of substitution rates on the phylogenetic input tree [54]. The analyses using likelihood-based (PTP) as well as the Bayesian approach (bPTP) were run using the web service (http://species.h-its.org/ptp/). MCMC chains for bPTP were run for 500,000 generations, sampling every 100 generations and discarding a burn-in of 10%. ABGD detects significant differences in intra- and interspecific pairwise distances (i.e. the barcoding gap) without an a priori species hypothesis [55]. The ABGD analysis was performed using the web interface at http://wwwabi.snv.jussieu.fr/public/abgd/abgdweb.html with default parameters and using Kimura 2-parameter (K2P) distances. The minimum relative gap width was set to different values between 0 and 1. K2P distance is widely used for DNA Barcoding [11], although it may be poorly justified as the model of choice for the character variation encountered in typical barcode datasets [56]. Differences in distance between best model and K2P model estimates are generally small and identification success rates are largely unaffected by model choice [57].

### Mapping of morphological characters

To study the congruence of coloration and other morphological patterns with DNA-inferred groups, the sequenced material was evaluated by scoring 29 multi-state morphological characters. These included characters of the meso-metaventral protrusion (MMV) as well as habitus characteristics like coloration, macrosetation and patterns of surface sculpture of various body parts (see S1 Text for a full list of character states and S1 Fig for detailed graphical explanation of most of the character states). Most of the evaluated characters were historically used for discrimination of species and/or subspecies (e.g. [34, 58, 59]). The characters were coded using NDE (Nexus Data Editor [60]) for all sequenced specimens (see S2 Text for full matrix). The coloration pattern was scored independently for nine separate body parts (namely: head, legs, mesepimeron, pronotum, basal emargination of pronotum, scutellum, elytra, pygidium, and the ventral body part) in order to describe the entire chromatic variability in detail. For the same reason, eight distinct color states ranging from black to vivid green were distinguished. We are aware that the classification of coloration, shine and sculptural patterns may be influenced by individual perception and external factors (e.g. illumination). Therefore, it was carried out under natural constant light conditions during a single session by a single researcher (PS). Although this approach might be biased by personal perception, we consider the habitus evaluation extremely important, as it is the most commonly used criterion for species separation in the subfamily Cetoniinae so far. See S1 Fig for images of the separate color states photographed under fluorescent light; a photo of a standard colorimetric chart (X-Rite Colorchecker passport) is provided for reference.

Each character was mapped on the resulting phylogenetic tree in Mesquite 3.10 [61] using parsimony for reconstruction of ancestral states and for each mapped character the consistency (CI) and retention index (RI) was calculated. In the search for diagnostic morphological characters, we subsequently investigated the morphological uniformity/variability of the identified clades, by observing the number of clades in which a given character was uniform (i.e. only one character stage per a clade) as well as the number of stable characters per clade.

### Shape analysis

To test the morphological differences between the traditionally described taxa and the molecular operational taxonomic units (MOTUs) as well as to test the morphological integrity of the newly identified clades, each of the 31 sequenced male specimens from the study was submitted to a body shape analysis. Additionally, we examined another 69 male specimens from the same or close collection series/localities/populations to increase the data set. To capture the maximum of the shape variability we used outlines of four distinct structures: the external outline of the left elytrum from the tip of the scutellum to the apex of the elytrum, the outline of the left half of pronotum, MMV (see S1 Fig) and the partial outline of the aedeagus. As default, we used the left paramere of the aedeagus. However, in 11 specimens the right paramere was overlapping the left one, so we used a mirror image of the right one (see S1 Fig). The extracted outline curves were subsequently converted into a set of 98 semi-landmarks and 2 landmarks using TPSUtil 1.44 [62]. These curves produce a measure that is independent of size in this sample. TpsRelw 1.49 [63] was employed to display the shape variation among the specimens. Landmarks were superimposed by a generalized Procrustes analysis; corresponding (homologous) landmarks were arranged over each other in a way that they would be as close to one another as possible by moving, scaling (enlarging or minimizing) and rotating them without changing their overall shape [64–66]. The program performs the relative warp analysis where relative warps are used to describe shape dissimilarity and to visualize it using D’Arcy Thompson’s transformation grids. The deformations in the grids represent the shape changes [67–69]. The results of the first two relative warps were afterwards plotted on an axis system and given different indication symbols using PAST v2.11 [70].

Subsequently, we tested the significance of the shape variations of groups defined under three different grouping criteria: (i) variation between the main clades identified by the mtDNA analysis (i.e. between clades 1–6, see “Results” for explanation); (ii) variation between the clades of “*Cuprea* complex” members only, i.e. clades 4, 5 and subclades of clade 6; (iii) variation between groups based on the currently recognized taxonomic system. We employed detrended correspondence analysis (DCA) and one-way nonparametric multivariate analysis of variance (NPMANOVA) on the relative warp scores matrix to test the significance of the variations between the groups. Additionally, we used multivariate analysis of variance (MANOVA) on a reduced dataset (i.e. a dataset containing only the groups with more than six members). Canonical variate analysis (CVA) was performed to illustrate these differences [71]. A graphical visualization of the DCA and CVA results representing the locations of the studied taxa was also performed. All these analyses were performed in PAST [70].

## Results

### Molecular trees and genetic divergence

The obtained gene fragments had a length of 779 base pairs (bp; *Cox1*) and 382 bp (*CytB*). The datasets comprised 52, 47, and 60 different haplotypes for *Cox1*, *CytB* and both genes combined, respectively. For individual gene analyses (results not shown here), we detected tree samples of two different subspecies co-occurring in Central Europe, i.e. two samples of *P*. *cuprea obscura* from the Czech Republic and a single Slovakian *P*. *cuprea metallica* sharing the same haplotype in *Cox1*. Similarly, we observed one sample of *P*. *c*. *metallica* from Italy sharing the haplotype with a Swiss *P*. *c*. *metallica* (*Cox1*) as well as with a *P*. *cuprea cuprea* from Corsica (*CytB*).

The overall topology was identical and well supported for all the trees obtained by both Bayesian inference (BI) and maximum likelihood (ML). However, the topology based only on the *CytB* alignment in both methods was inconsistent with those of *Cox1* and concatenated datasets (Fig 1), most likely due to the shortness of this fragment, which led to a very low resolution within clades 4 and 6. All the main nodes were marked by Roman numerals (I—VII), while the clades were marked with Arabic numerals (1–6) and with letters where necessary (Fig 1). All were well supported in both analyses (BI, ML), except the split VI with maximum posterior probability (PP = 1.00), but low bootstrap support (BS = 69). Two other exceptions inside of the clade 6 are mentioned further in the text.

In the phylogenetic tree, individuals of *P*. *opaca*, *P*. *fieberi* and *P*. *angustata* each formed a distinct monophyletic group (clades 1–3; Fig 1). *Potosia angustata* (clade 3) was identified as a sister group of the entire *P*. *cuprea* species complex (node I), including representatives of *P*. *cuprina* and *P*. *hypocrita*. The next split (node II) divided clade 4 with samples from Cyprus, Asian Turkey, and the Levant, traditionally assigned as *P*. *cuprea ikonomovi*, *P*. *cuprea ignicollis* and *P*. *cuprina*, respectively, from the rest of *P*. *cuprea* species complex. Thus, given the traditionally recognized taxa, *P*. *cuprea* did not show monophyly. The node III represents a split between the Sicilian population of *P*. *hypocrita* (clade 5) from clade 6 containing *P*. *cuprea* “sensu stricto” (i.e. European mainland, Balearic Islands, Corsica, and Sardinia, hereafter also referred to as the European clade or clade 6).

Among *P*. *cuprea* “sensu stricto”, the Crimean population of *P*. *cuprea volhyniensis* in clade 6A resulted as a sister group to all other populations (node IV), followed by Greek samples of *P*. *c*. *obscura* in clade 6B (node V). Clade 6B was subdivided into two groups due to a single sample from the Peloponnese, but the branch was weakly supported (posterior probability (PP) = 0.94, bootstrap value (BS) = 66). Node VI separated the central European populations (6C) from the southern clades (6D, 6E, 6F). The central European clade comprises specimens assigned to *P*. *c*. *metallica*, *P*. *c*. *obscura*, but also one specimen of *P*. *c*. *bourgini* from northern part of France. The relationship among the southern European clades (node VII, clade 6D, 6E, 6F) remained largely unresolved, although the clades themselves were fully supported: clade 6D from the Iberian Peninsula (*P*. *c*. *brancoi*), clade 6E with individuals from northeastern Greece and European Turkey (*Potosia* sp. “Thracian population”) and finally clade 6F from Italy, Sardinia, Corsica, southern France and southern Switzerland. According to the current taxonomy, the following subspecific taxa were assigned to the latter clade: nominotypic *P*. *cuprea cuprea*, *P*. *c*. *bourgini*, *P*. *c*. *olivacea* and *P*. *c*. *metallica*. This clade (6F) also included one sample from Adriatic Croatia (Brač island), but its position was not congruent between the analyses (BI vs. ML) and gained lower support (PP = 0.93).

The highest genetic divergence (Table 1) was observed between *P*. *opaca* (clade 1) and the remaining taxa (10.18–13.29%). Similarly, a high divergence (8.98–10.19%) was observed among the *P*. *cuprea* species complex and the clades of *P*. *fieberi* and *P*. *angustata* (clades 2 and 3), respectively. The average distance of clade 4 (specimens assigned to *P*. *cuprina*, *P*. *cuprea ignicollis* and *P*. *cuprea ikonomovi*) and the clades 5 and 6 ranged from 8.81 to 9.02%. The distance between the Sicilian *P*. *hypocrita* (clade 5) and the other European populations of *P*. *cuprea* (clade 6) resulted to be 6.71%. Within clade 6 (*P*. *cuprea* “sensu stricto”), the most divergent population was the Crimean *P*. *c*. *volhyniensis* (6A), with an average genetic distance of 3.63–4.12% from the other clades. The distance between the remaining clades (6B–6F) was rather low (1.27–2.31%). The mean distance within the whole clade 6 was 1.90%, but within each specific subclade (6A–6F) this value was much lower (0.26–0.96%).

**Table 1:** Mean average genetic distances inside and between main phylogenetic clades and subclades specified in Fig 1.

**Clade**	**1**	**2**	**3**	**4**	**4A**	**4B**	**5**	**6**	**6A**	**6B**	**6C**	**6D**	**6E**	**6F**	
**Mean distance**	0,0159	0,0033	0,0098	0,0170	0,0132	0,0034	0,0000	0,0190	0,0074	0,0069	0,0096	0,0048	0,0026	0,0095	
**Standard error**	0,0031	0,0011	0,0024	0,0024	0,0032	0,0010	0,0000	0,0020	0,0020	0,0014	0,0017	0,0014	0,0012	0,0017	
**Clade**	**1**	**2**	**3**	**4**	**5**	**6**	**Clade**	**4A**	**4B**	**6A**	**6B**	**6C**	**6D**	**6E**	**6F**
**1**	-	0,0092	0,0106	0,0110	0,0113	0,0100	**4A**	-	0,0056						
**2**	0,1018	-	0,0086	0,0098	0,0096	0,0086	**4B**	0,0410	-						
**3**	0,1227	0,0859	-	0,0093	0,0092	0,0083	**6A**			-	0,0056	0,0057	0,0062	0,0062	0,0059
**4**	0,1321	0,1019	0,0974	-	0,0086	0,0082	**6B**			0,0363	-	0,0035	0,0039	0,0045	0,0038
**5**	0,1329	0,0898	0,1016	0,0902	-	0,0070	**6C**			0,0374	0,0186	-	0,0027	0,0037	0,0026
**6**	0,1249	0,0869	0,0992	0,0881	0,0671	-	**6D**			0,0412	0,0199	0,0127	-	0,0041	0,0032
							**6E**			0,0412	0,0231	0,0171	0,0180	-	0,0037
							**6F**			0,0392	0,0215	0,0141	0,0156	0,0182	-

Calculation was done for a concatenate of *Cox1* and *CytB* genes using the Kimura 2-parameter model with standard errors estimated using 1000 bootstrap replicates (grey cells).

### DNA-based species delimitation

The results of Poisson Tree Processes (PTP) and Automatic Barcode Gap Discovery (ABGD) analyses based on separate gene datasets (Fig 1) showed quite consistent results. They were mostly congruent with the tree topology and its recognized main clades and subclades. However, the PTP analysis of the *CytB* dataset represented a remarkable exception, as the recognized clades (i.e. putative species units) were “over-split”. Similarly, ABGD of *CytB* dataset lumped clades 4 and 5 with the *P*. *cuprea* “sensu stricto” (clade 6). These results may be highly influenced by the shortness of the analyzed fragment, thus we refrain from commenting on them further in the text.

Populations of *P*. *opaca* were well separated in both analyses (with additional splitting into two subgroups identified by the PTP method for the *Cox1* dataset). Samples of *P*. *fieberi* were recognized as a single group in all analyses, as well as those of *P*. *angustata*. Clade 4 was separated into at least two subgroups (clade 4A vs. 4B) in both analyses. Clade 4A was divided into two subunits in PTP for *Cox1*, separating *P*. *cuprea ikonomovi* from the Turkish specimen of *P*. *cuprina*. *Potosia hypocrita* resulted as a distinct group in all analyses. Clade 6A with *P*. *cuprea volhyniensis* was recognized as an independent group from the rest of the samples of clade 6 in the ABGD and PTP based on the *Cox1* dataset.

### Haplotype networks

**Fig 2:**
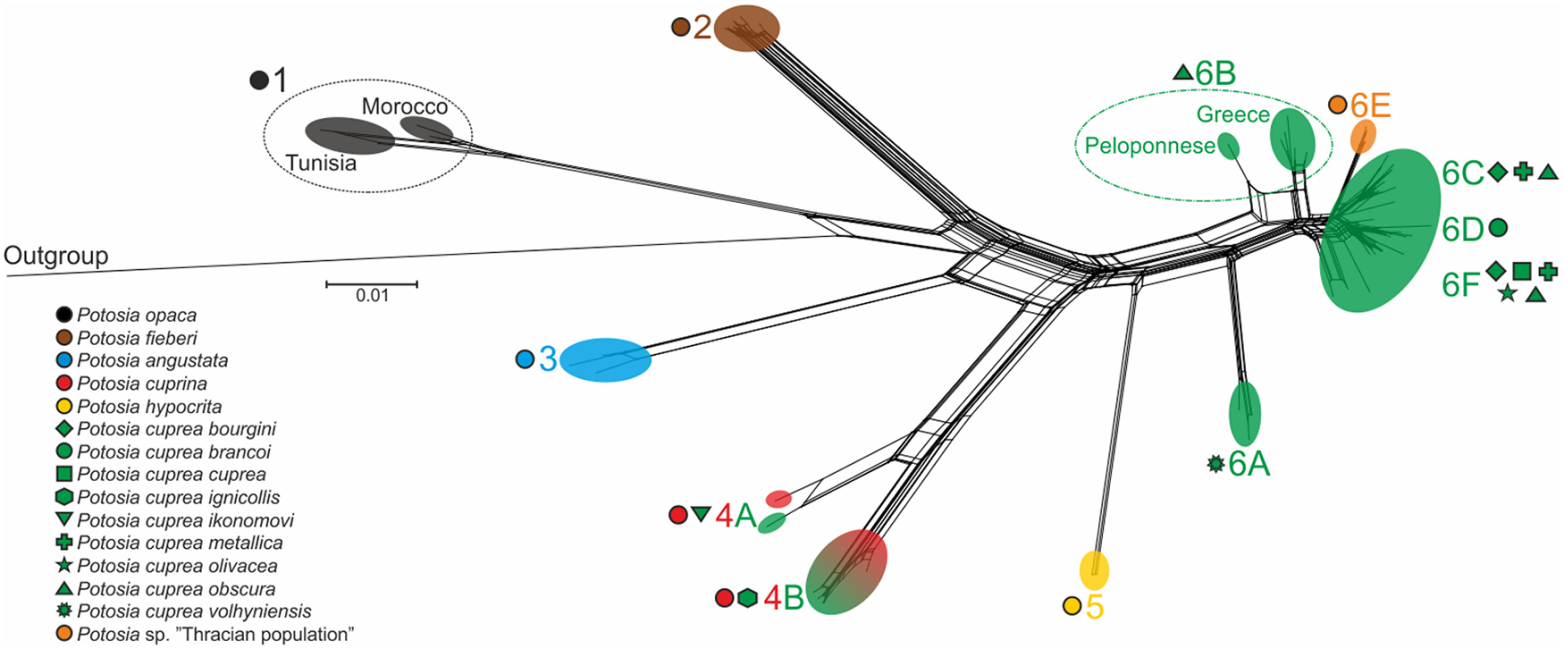
Neighbor-joining network computed in SplitsTree. Results of statistical parsimony analysis calculated in TCS software are highlighted using filled ellipses with taxon-specific color and symbols with captions of MOTUs (both analyses based on the concatenate of *Cox1* and *CytB* fragment).

**Fig 3:**
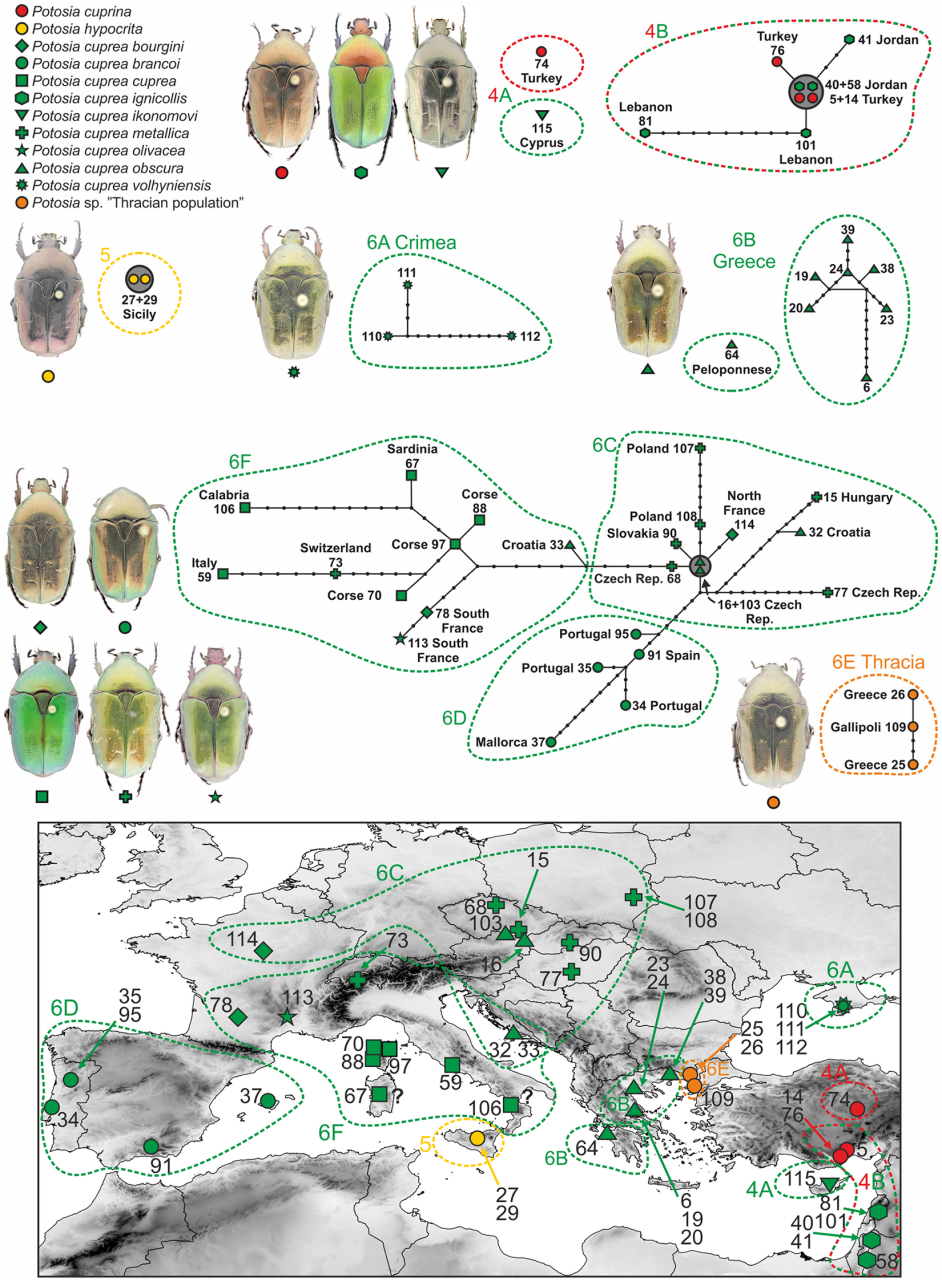
Detailed structure of statistical parsimony networks compute in TCS using the concatenate of *Cox1* and *CytB* fragment for clade 4, 5 and 6. The same symbols and captions as in Figs 1 and 2. The map shows a distribution of vouchers included in the calculated networks and their clusters.

Statistical parsimony analysis using TCS based on the concatenated dataset resulted in 13 independent networks (excluding outgroup), which were mapped onto the neighbor-joining (NJ) network calculated in SplitsTree (Fig 2). The number of steps at which two haplotypes have a 95% statistical probability of being linked without homoplasy was calculated to be 14. Separate clusters from TCS matched the major clades of the presented phylogenetic tree and the topology of the NJ network, with the following exceptions: (i) the *P*. *opaca* clade was separated into two clusters corresponding with the geographical origin of the samples; (ii) clades 4 and 6 were split into several separate clusters (Fig 3).

Clade 4 showed a structure with the central haplotype comprising two samples of *P*. *cuprea ignicollis* from Jordan and two samples of *P*. *cuprina* from Turkey. Other samples were quite close to this centrally positioned haplotype except for *P*. *cuprea ikonomovi* from Cyprus and *P*. *cuprina* from central Turkey (both as singletons), which resulted in two separate networks with a distance of 43 and 49 mutations, respectively, from the main network and 16 mutations from each other (Fig 3). Clade 5 formed an independent cluster containing two Sicilian specimens of *P*. *hypocrita* with matching haplotypes (Fig 3).

Clade 6 was split into five different networks (i—v): (i) the first network identified by the analyses resulted identical to clade 6A and contained specimens of *P*. *cuprea volhyniensis* from the Crimean Peninsula, separated by 39 mutations from the other network (ii), which includes the Greek *P*. *cuprea obscura* (clade 6B). A single sample of *P*. *c*. *obscura* from the Peloponnese was identified as an independent singleton network (iii) with 17 mutation steps from the remaining Greek samples (ii). The fourth network (iv) comprised the individuals of clade 6E “Thracian population” from eastern Greece and European Turkey with 15 mutations from the central network (v). The central network, which was 16 mutation steps from the network (ii) composed of Greek specimens, contained all other samples (e.g. members of clades 6C, 6D, 6F) and formed three branches. One of these branches contained samples of *P*. *cuprea brancoi* from the Iberian Peninsula and the Balearic Islands (clade 6D). The second contained individuals of *P*. *cuprea cuprea* from the Apennine Peninsula, Corsica and Sardinia (clade 6F) along with the specimens of *P*. *cuprea bourgini* and *P*. *cuprea olivacea* from southern France and one individual morphologically assigned to *P*. *cuprea metallica* from southern Switzerland. The last branch was composed of individuals from central parts of Europe and comprised samples of *P*. *c*. *obscura* and *P*. *c*. *metallica* from Hungary, Slovakia, Czech Republic and Poland, and one sample of *P*. *c*. *obscura* from Adriatic Croatia (BI; clade 6C). This branch contained also one specimen of *P*. *c*. *bourgini* from northern France. One sample of *P*. *c*. *obscura* from Croatia was identified as an intermediate, being located between the Central European branch and the populations of the Apennine Peninsula (Fig 3). Statistical parsimony analyses using TCS were calculated for individual genes and mapped on the phylogenetic tree as well (Fig 1). Results of these analyses were broadly matching the outcomes of ABGD and PTP, moreover they were congruent with the output of TCS for concatenate data (Figs 2 and 3).

### Molecular dating

**Fig 4:**
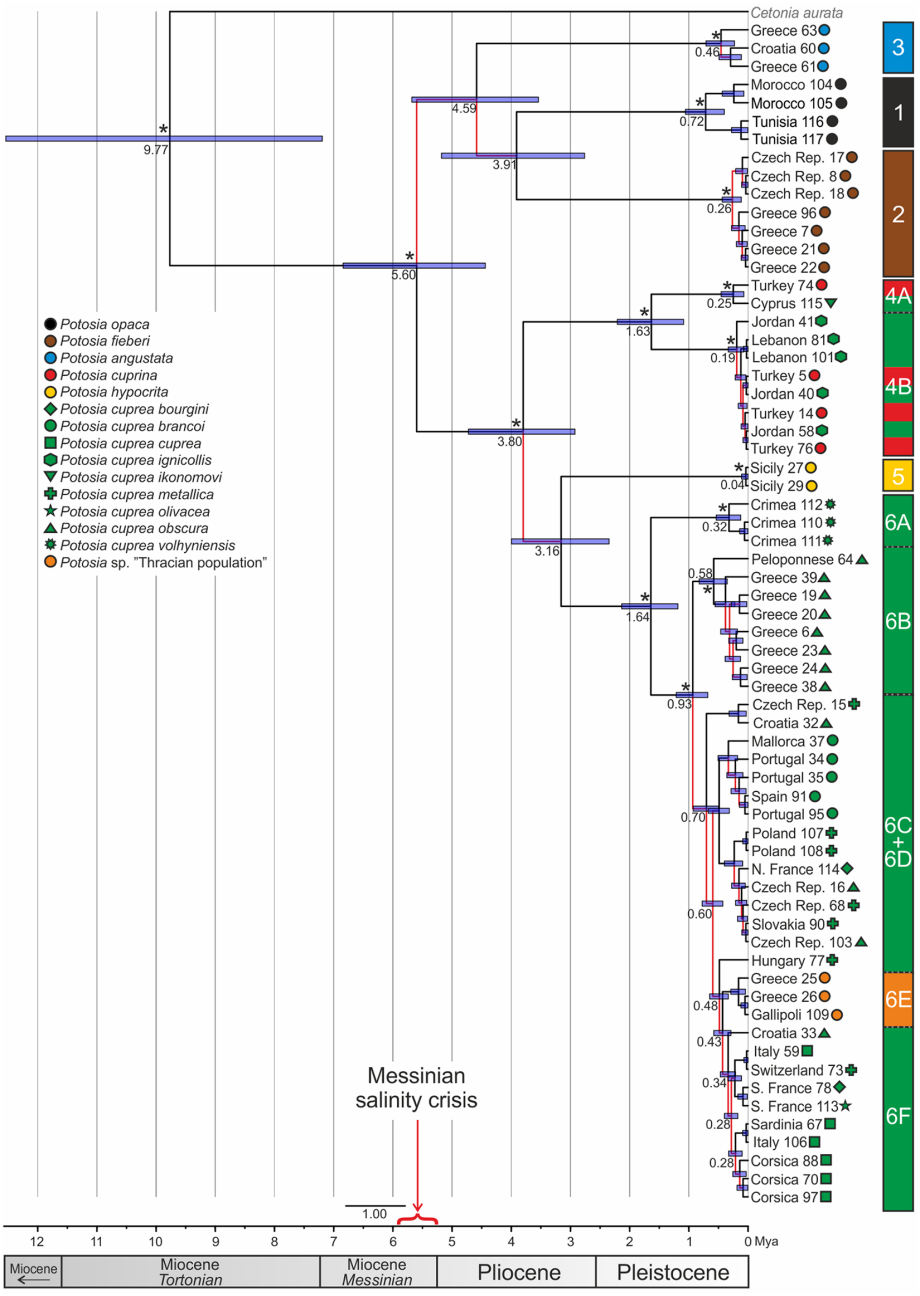
Time-calibrated phylogram calculated in BEAST using the *Cox1* dataset. Numbers on nodes are estimated dates of diversification with resulting confidence intervals (blue bars). Posterior probabilities (PP) resulting from Bayesian inference analyses are marked with an asterisk (PP = 1.00) for the main splits. Red colored branches indicate low support value (PP < 0.95).

The age of the split between the well-established *Potosia* species (clades 1–3) and the clades comprising the *P*. *cuprea* species complex (clades 4–6) was estimated between 6.84–4.44 Mya, i.e. during the Messinian stage (7.25–5.33 Mya) of Miocene or the Zanclean stage (5.33–3.60 Mya) of Pliocene (Fig 4). The splits between clades 1, 2, and 3 as well as the split between the clade 4 and clade 5+6 were estimated primarily to have occurred during Pliocene (5.3–2.58 Mya) with the confidence intervals varying between 5.68–2.76 Mya. The age of the split of the Sicilian *P*. *hypocrita* (clade 5) from *P*. *cuprea* “sensu stricto” (clade 6) was estimated between 4.00–2.35 Mya. The radiation of *P*. *cuprea* “sensu stricto” in clade 6 was estimated to have occurred during Pleistocene with the oldest split of clade 6A (*P*. *cuprea volhyniensis*) dated between 2.14–1.18 Mya and the subsequent split of clade 6B (Greek *P*. *cuprea obscura*) between 1.21–0.68 Mya. The rest of the splits correspond to late Pleistocene, with varying intervals between 0.93–0.33 Mya.

### Morphological character mapping

**Fig 5:**
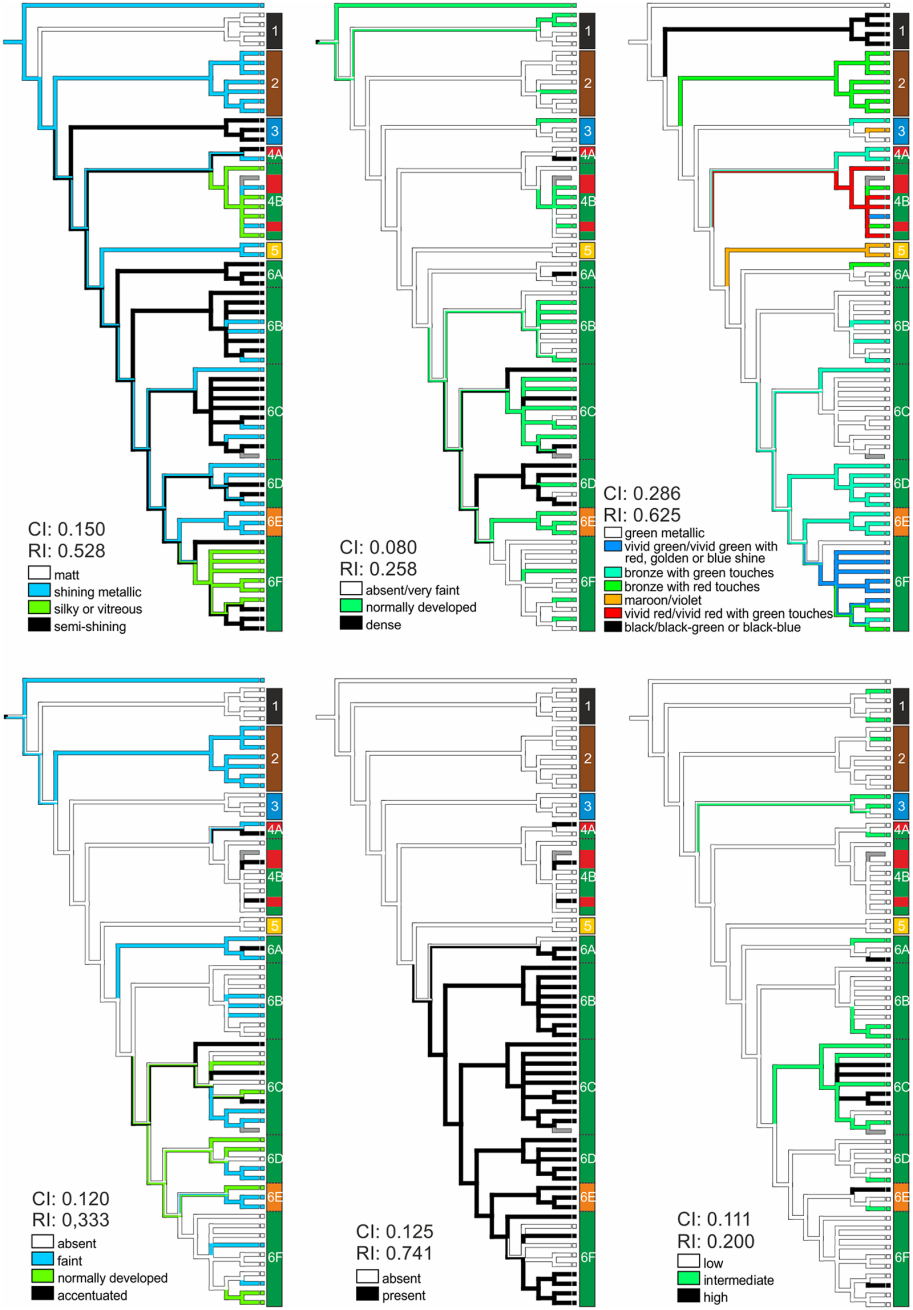
Six morphological characters mapped on the phylogenetic tree from Fig 1, along with the legends for each tree. Upper row from left: shine of the dorsal body face, ventral macrosetation and color of the pronotum. Lower row from left: white markings on elytra, white markings on the “knees” and punctation coarseness on the meso-metaventral (MMV) protrusion. Consistency index (CI) and retention index (RI) are shown for each tree.

With the exception of character 29 (reflection of left-handed polarized light; CI = 1, RI = 1), which was present in all metallic forms, we found no congruence of any character or character state with the entire tree topology (matrix in S2 Text). Usually these characters were highly variable both within and between the identified clades, especially within the European populations of *P*. *cuprea* in clade 6 (Fig 5, S2 Fig, S3 Table). The individual CI of all characters ranged between 0.074–0.333. The highest value of CI was observed in character 10 and 14 (i.e. color of pronotal emargination and color of ventral body parts, respectively). However, these characters might be influenced also by the high number their character states (8) in these characters. The highest RI (0.741) was observed in character 19 (most proximal part of meso- and metatibiae with (or absent from) whitish patches laterally, hereafter abbreviated as white markings on the “knees”). The values of both indexes for individual characters are given in S1 Text and S2 Fig.

To infer the taxonomical relevance of the characters, we observed the character state stability within the clade (i.e. number of achieved character states per character in a given clade) and the morphological stability of a given clade (i.e. the number of characters with only single character state per clade; see S3 Table). Character 19 (presence or absence of white markings on “knees”) showed the highest consistency regarding clade stability. White markings were present in almost all members of clade 6, though also in some specimens of clade 4 (Fig 5). With no surprise, the highest morphological stability was observed in the chromatically uniform clades (*P*. *opaca*, *P*. *hypocrita*), with only 5 and 2 variable characters, respectively. *Potosia fieberi* was also rather uniform in coloration, macrosetation, and patterns of punctation, but it exhibited a variable morphology of MMV. In addition, the clade of *P*. *angustata* appeared morphologically uniform (with only 8 variable characters), although this may be influenced by the limited number of examined specimens. Clades 4 and 6 contained the most variable phenotypes. In both clades, almost no character was found which would follow either the traditional taxonomic groupings or the reconstructed tree topology. Still, some clade-specific character states have been observed, e.g. the particular coloration and shine patterns of *P*. *cuprea cuprea* or coloration of the head in *P*. *cuprea brancoi*.

### Geometric morphometrics

**Fig 6:**
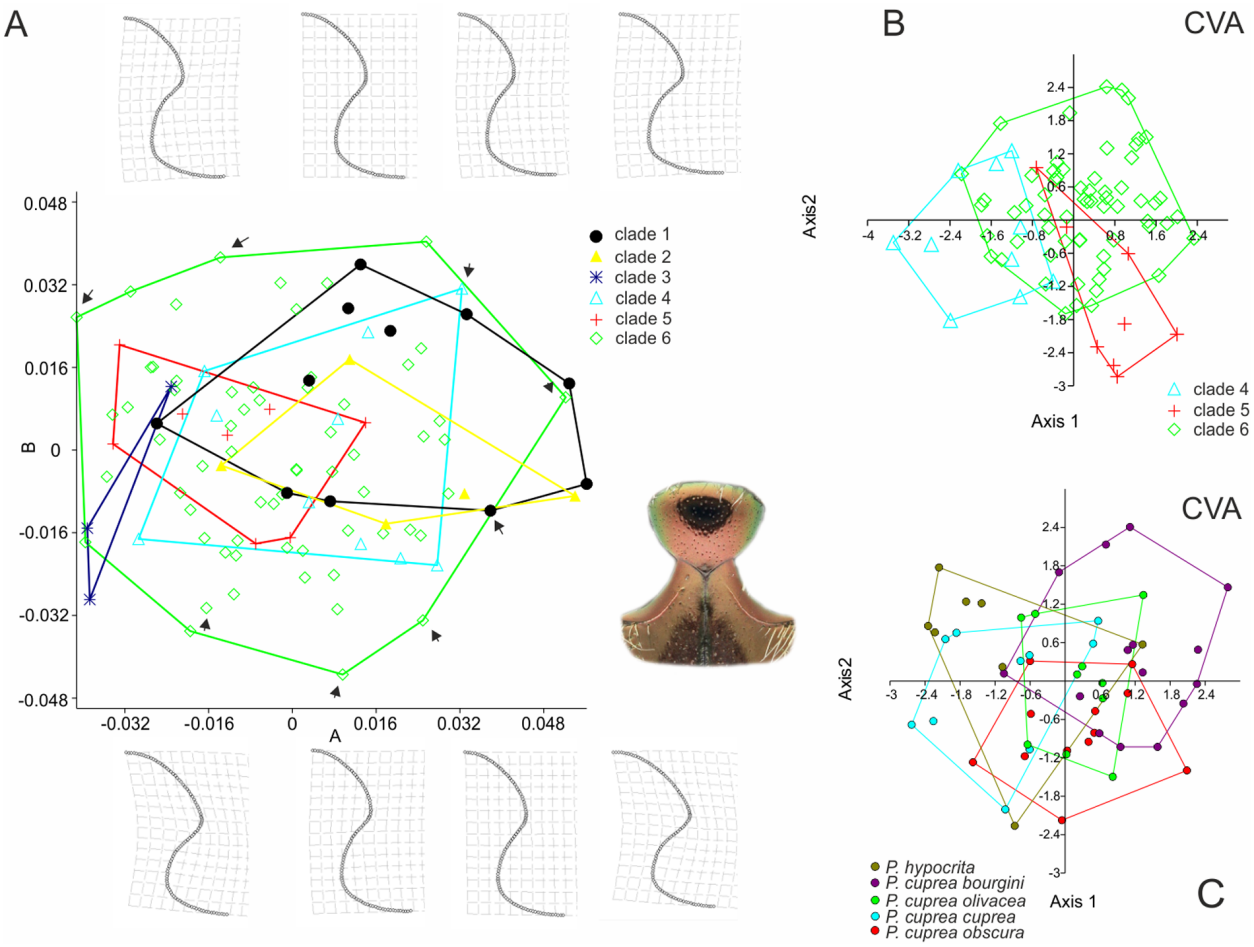
Morphometric shape analysis of the partial analysis of meso-metaventral protrusions (MMV; colored inset). A) Scatter plot based on the relative warp scores, with group members corresponding to the clades identified by the molecular tree in Fig 1. Insets represent variation in thin-plate spline (TPS) transformation grids of MMV for selected specimens. B—C) CVA plots with groups according to clades 4, 5 and 6 (B), and according to the currently recognized taxonomic units (C).

**Fig 7:**
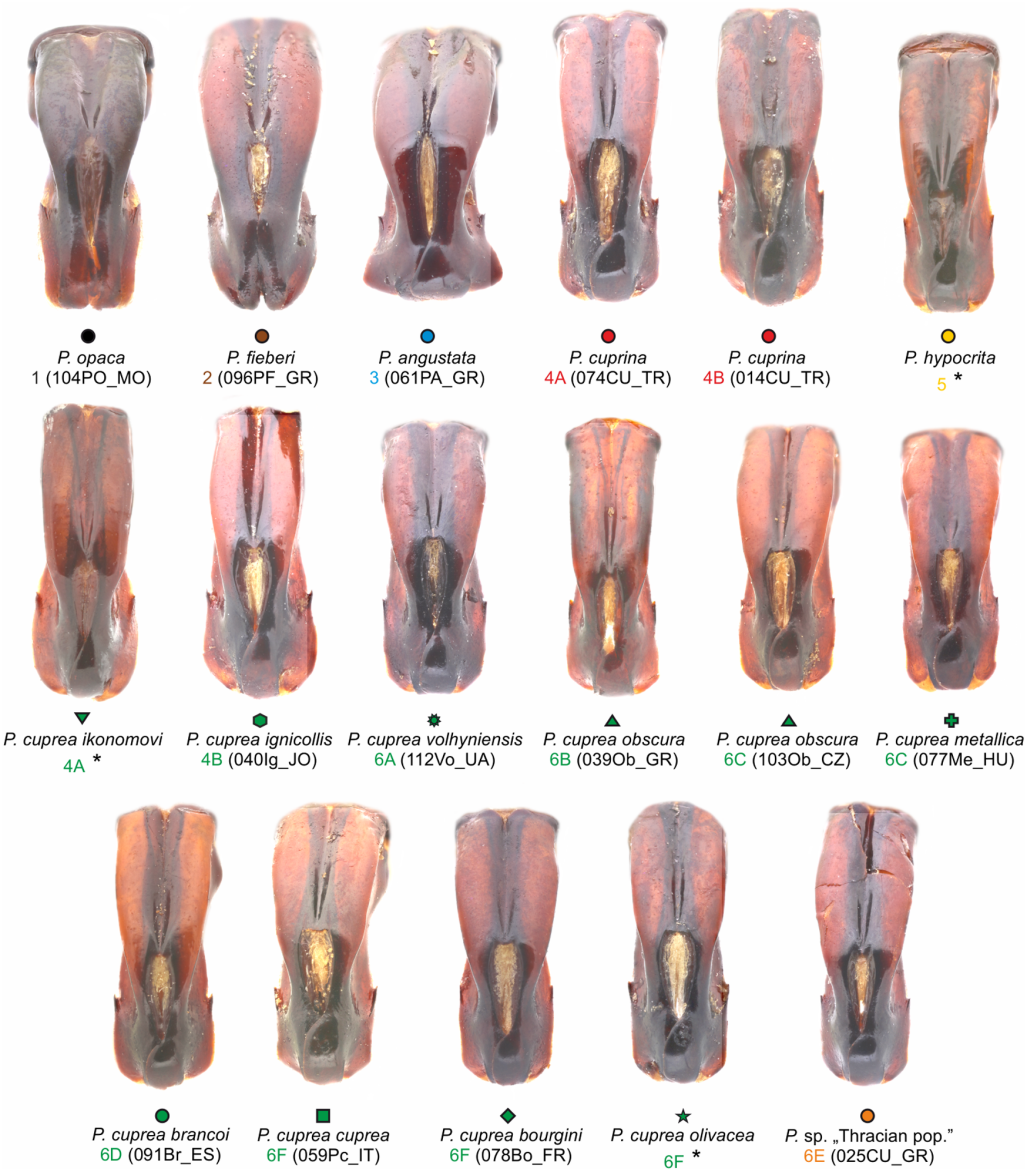
Male genitalia of voucher specimens included in the analyses. Three taxa marked with an asterisk (specimens with identical collecting area and date–*P*. *hypocrita*, *P*. *cuprea olivacea* or at least from the same population (Cyprus)–*P*. *cuprea ikonomovi*).

Out of the four structures analyzed with morphometrics, the shape of MMV (Fig 6) and the aedeagus seemed to be the most informative, with 95% of cumulative variability explained by 5 or 7 eigenaxes, respectively. However, the results of the shape analysis did not reveal any unambiguous match between the shape of any of the four structures and the three main clades of mtDNA tree (i.e. clade 4, clade 5, and subclades of clade 6). The DCA plots (S3 Fig), as well as the CVA scatter plots, resulted widely overlapping, both under the molecular or taxonomic criteria. The MANOVA (Table 2) analysis of MMV was the most congruent with the “main clades” grouping, with significant differences between clades 6 and 4, and 6 and 5. On the other hand, the confusion matrix showed an unreasonably high error rate in the “a posteriori” identification of clade memberships (results not shown here). In addition, the scatter plot based on the relative warp scores (S3 Fig) and the CVA plot resulted widely overlapping. Also, the shape of parameres observed in members of clades 4–6 have proved to be not very clade- or taxon-specific but rather variable among individuals (Fig 7, S3 Fig).

**Table 2:** Amount of variability explained by the first three eigenaxes identified by relative warps analysis.

Structure	Elytra		Aedeagus		MMV		Pronotum	
**no of eigenaxes > 95% of var**.	**12**		**7**		**5**		**9**	
**eigenaxis 1**	41.08%	41.08%	50.98%	50.98%	76,51%	76,51%	62.73%	62.73%
**eigenaxis 2**	15.26%	56.34%	24.37%	75.35%	8,93%	85,43%	13.92%	76.65%
**eigenaxis 3**	12.82%	69.16%	9.25%	84.60%	5,95%	91,39%	6.93%	83.59%

**Lower Section**:												
**p-values CVA/clades**	**4**	**5**	**6**	**4**	**5**	**6**	**4**	**5**	**6**	**4**	**5**	**6**
4	-	0.14551	0.00717	-	0.25747	0.11320	-	0.056883	0.00044	-	0.09746	0.00040
5	0.14551	-	0.06020	0.25747	-	0.00011	0.056883	-	0.01433	0.09746	-	0.06694
6	0.00717	0.06020	-	0.11320	0.00011	-	0.00044	0.01433	-	0.00040	0.06694	-

First column variability explained by the respective axis, second column in cumulative percent. Lower section: results of the MANOVA test of the pairwise shape differences of the meso-metaventral protrusion (MMV) among clades 4, 5, 6 (significant values in grey cells).

## Discussion

Here we present the first comprehensive phylogenetic analysis of *Potosia cuprea* and related species in Europe and adjacent regions, combining data from mtDNA genes, geometrical morphometry, and classical morphology. Our molecular analyses have revealed the existence of three well-supported and geographically differentiated major clades (4–6) within the Western Palearctic *P*. *cuprea* species complex. The taxonomic status of these clades remains unclear and the results of our analyses contradict the currently accepted taxonomical hypotheses of the group as well as the alternative hypotheses. Based on the output of the molecular analyses three alternative hypotheses can be derived.

(i) Clades 4, 5, 6 represent three different species. Clade 4 would therefore join *P*. *cuprina* with two subspecies of *P*. *cuprea* (*P*. *c*. *ignicollis* and *P*. *c*. *ikonomovi*), Clade 5 corresponds with the Sicilian *P*. *hypocrita* and clade 6 contains *P*. *cuprea* with all the remaining European subspecies.
(ii) Clades 4, 5, 6 represent three deeply separated lineages of a single species (*P*. *cuprea*) and they are sub-specific.
(iii) Clade 4 is elevated to species status (joining *P*. *cuprina* with two subspecies of *P*. *cuprea* (*P*. *c*. *ignicollis* and *P*. *c*. *ikonomovi*), while clades 5 and 6 represent two deeply separated lineages of *P*. *cuprea* (with possible sub-specific status).

While the first scenario is well supported by the wide congruence of the DNA-based species delimitation methods and by the geographical distribution of the clades, the other two scenarios (ii, iii) cannot be entirely rejected due to the absence of clear morphological characters supporting or separating the clades. The average pairwise genetic distances of *Cox1* between these three clades (4–6) are well above the interspecific divergence range published for pleurostict Scarabaeoidea [52, 72–75], but see [13]. The distances within subclades of clade 6 range from 1.27 to 4.12% and correspond largely with the consensually accepted sub-specific values.

To date, only few European Cetoniinae species have been investigated for their genetic structure. Audisio et al. [7] revealed a hidden diversity linked to glacial refugia of the European hermit beetle *Osmoderma eremita* (Scopoli, 1763) species complex based on the *Cox1* sequences. The reported genetic distances between *O*. *cristinae* Sparacio, 1994 from Sicily and *Osmoderma* LePeletier & Audinet-Serville, 1828 populations from mainland Italy were between 6.0 and 6.8%. These results were corroborated later by Zauli et al. [76]. The phylogeographic analysis of the European rose chafer (*Cetonia aurata*) [13] showed incongruent results from nuclear, mitochondrial, and morphological datasets. The average pairwise genetic distance between the endemic Sicilian subspecies *Cetonia aurata sicula* Aliquó, 1983 and populations of *Cetonia aurata pisana* Heer, 1841 from the Apennine peninsula was 2.1% using the mtDNA marker *Cox1*. On the other hand these values have to be interpreted with care as other, largely sympatric haplotype lineages of *Cetonia aurata aurata* (Linnaeus 1758) showed divergence rates of 8–9% (i.e. values typically found between good established species even within the genus *Cetonia* Fabricius, 1775 [13]. This might indicate possible incomplete lineage sorting with subsequent hybridization or gene introgression linked to areal fragmentation during glacial periods or other evolutionary or human induced scenario [13].

The estimated timing of the basal splits in the *Potosia cuprea* complex provides additional support for the first scenario. The main speciation event i.e. the split between clade 4 and 5+6 occurred after the Messinian salinity crisis (5.96–5.33 Mya) indicating a post-Messinian colonization of Sicily by *P*. *hypocrita*. The yet outstanding examination of the Maltese populations of *P*. *hypocrita* [77] and the enigmatic *P*. *mayeti* (Le Compte, 1906) from Libya could further elucidate the origin of this lineage.

Under the assumption that clades 4, 5 and 6 represent separate species, *P*. *cuprea*, under the current nomenclatural treatment (see [25]), is polyphyletic, especially in regard to *P*. *cuprea ignicollis* and *P*.*cuprea ikonomovi* which both formed a clade together with *P*. *cuprina* (clade 4). The TCS analysis of concatenated data recognized identical haplotypes shared by individuals assigned to *P*. *cuprina* and *P*. *cuprea ignicollis*. However, based on our data we are not able to tell whether if this is a result of crossbreeding of individual beetles, recent gene flow, incomplete lineage sorting, gene introgression or other mechanism. Due to the limited number of sampled individuals and the absence of nuclear markers in our dataset we refrain from any taxonomical interpretation.

The results of our analysis confirmed the Sicilian *P*. *hypocrita* so far as a separate evolutionary entity with the estimated divergence time from the rest of the European *P*. *cuprea* between 4.00–2.35 Mya (i.e. Pliocene or onset of Pleistocene). The status of this taxon has been already addressed by Sparaccio [78] and widely accepted in subsequent literature (e.g. [58, 25]). This taxon is also characterized by comparatively stable morphological and coloration pattern, which is an unusual among the members of *P*. *cuprea* species complex.

### The topology of *P*. *cuprea* “sensu stricto” and the contradicting taxonomical hypotheses

The observed topology of *P*. *cuprea* “sensu stricto.” (clade 6) does not correspond to any of the previously proposed classifications of the species. *Potosia cuprea volhyniensis* and *P*. *cuprea brancoi* were the only subspecies forming monophyletic clades (6A, 6D), with the former clade being well separated and genetically most distant from the remaining members of the clade 6. *Potosia cuprea obscura* appeared polyphyletic with our dataset, as individuals assigned to this subspecies were recovered at least in three unrelated positions (clades 6B, 6C, 6E). Clade 6B contained exclusively specimens of “typical *obscura* morphotype” as well as slightly aberrant beetles from eastern Greece (still morphologically assignable to *P*. *c*. *obscura*) and were shown to be a sister to all other clades except of clade 6A. Clade 6C contained beside the morphotypes of *P*. *c*. *obscura* also *P*. *cuprea metallica* and *P*. *cuprea bourgini*. The nominotypic subspecies *P*. *cuprea cuprea* from the Apennine peninsula, Sardinia and Corsica also appeared paraphyletic as it was recovered within the clade 6F together with other individuals assigned to *P*. *c*. *bourgini*, *P*. *c*. *metallica* and *P*. *cuprea olivacea* all geographically limited to the Adriatic-Mediterranean region (including south-western parts of France). These results strongly challenge the recently proposed elevation of the subspecies *metallica* as a separate species ([34, 79, 80, 24, 58] see below) as well as the taxonomic status of most of the recognized subspecies. All splits in the topology of the clade 6 were estimated in Pleistocene between 2.14–1.18 Mya (clade 6A), 1.21–0.68 Mya (clade 6B) and the rest in varying intervals between 0.93–0.33 Mya, inducing the idea, that *P*. *cuprea* species group could be highly affected by the climatic fluctuations during glacials and interglacials, which resulted in high number of different morphs over the Europe. Our results confirm that populations of *P*. *cuprea* found on the western Mediterranean islands (e.g. Mallorca, Corsica and Sardinia but not Sicily) are likely to originate from the adjacent mainland populations.

The results of TCS analysis of clade 6 were mostly congruent with the tree topology and revealed some geographical patterns. The highest haplotype variability was observed in the eastern parts of distribution range. Three out of the five haplotype networks were identified in the southernmost parts of Balkan peninsula, isolated by the Hellenides and the Pirin mountain range. Another network was composed solely of beetles from Crimea. The last TCS network includes beetles from the remaining part of Europe and again exhibits a distinct geographical structure with tree branches composed of Atlantic-Mediterranean, Adriatic-Mediterranean and central European samples, respectively.

### *Potosia cuprea metallica* versus *Potosia metallica*

In the recent history, one of the most discussed taxonomical questions concerning the *P*. *cuprea* species group has been the status of *P*. *cuprea metallica* as a separate species outside *P*. *cuprea*. This view initially introduced by Medvedev [34] was, however, rejected by Mikšić [21], Baraud [23] and Krajčík [81, 35]. It was later resurrected by Alexis & Delpont [79, 80] followed by Smetana [82] and Tauzin [58]. Tauzin [58] summarized arguments in favor of this treatment, under which he made a list of the morphological characters (e.g. shape of prescutellum, humeral emargination and punctation of elytra) but also noted ecological differences of the presumed separate species. The most serious argument stressing his point of view was the inability of *P*. *c*. *metallica* to interbreed with the other taxa (e.g. *P*. *c*. *cuprea*, *P*. *c*. *bourgini*, *P*. *c*. *olivacea* and *P*. *cuprina*) for more than five generations in artificial breeding experiments, followed by the assumption that larvae of *P*. *c*. *metallica* are strictly associated with ant nests. Tauzin [58] also excludes the previously proposed subspecies *P*. *metallica bourgini* as well as the Iberian *P*. *metallica brancoi* from *P*. *metallica* and re-classifies those two taxa as subspecies of *P*. *cuprea*.

Our results indicate a possibility of a gene flow between the different morphotypes of *P*. *cuprea* as we found identical haplotypes between several *P*. *cuprea* subspecies in both mitochondrial genes. One of the many explanations for this can be a crossbreeding between different subspecies/populations of *P*. *cuprea*. As demonstrated above, our results contradict species rank status of the form “*metallica”*. In fact, we do not have support even for the validity of the subspecies *P*. *cuprea metallica*. Beside the non-polarized morphological variation of the *cuprea* complex, the alleged ecological differences as the strict tendency to myrmecophily and utilization of coniferous tree substrate (needles, rotten wood) does not hold for the entire distribution area of *P*. *c*. *metallica* [31]. Moreover, we have repeatedly reared larvae of *P*. *c*. *metallica* from various types of microhabitats in Czech Republic including organic soil, hollows of trees, compost heaps, rotting wood of deciduous trees or heaps of rotting coniferous bark, as well as from ant nests (genus *Formica* Linnaeus, 1758). The crossbreeding abilities of the French and Central-European populations should be tested thoroughly along with an *in situ* genetic research of the hybridization zones which were reported by Décobert & Stéphany [27]. The absence of the reproductive isolation mechanisms on chromosomal level was demonstrated by Dutrillaux et al. [32] who analyzed the karyotypes of five European *P*. *cuprea* subspecies. The authors found that *P*. *c*. *bourgini*, *P*. *c*. *brancoi*, *P*. *c*. *cuprea* and *P*. *c*. *metallica* share identical karyotypes, while *P*. *cuprea obscura* from Greece varies slightly from the others in the presence of heterochromatin on the shorter arm of X chromosome. With other words, populations which were according to our results more related have identic karyotypes, while those from more distant lineages show some (minor) differentiation.

### Morphology and coloration pattern—Geographical rather genealogical determination or a neutral variability?

The morphological variation of the *P*. *cuprea* species complex does not follow the observed phylogenetic structure and species entities derived from mitochondrial DNA. This was especially obvious in the clades containing more than one traditional subspecies, e.g. clades 4, 6C and 6F. This applies also for some of the so far retained crucial taxonomical characters as coloration of several body parts, macrosetation, punctation and structure of elytral and discal surface of pronotum as well as the shape of MMV and aedeagus [34, 58, 59].

The sculpture of the distal part of the elytra and white markings on the “knees” proved to be the most clade-specific characters. However, none of these is of unambiguous taxonomical relevance. Moreover, geometric morphometry (GM) analysis also demonstrated a somewhat gradual rather discrete variation in the shape of the humeral emargination, MMV and anterior scutellar margin (results not shown here) or shape of aedeagus in the *P*. *cuprea* species complex (Fig 7). Interestingly, GM analyses of the MMV shape (Fig 6) also failed to discriminate the traditionally accepted subspecies of *P*. *cuprea*, questioning the taxonomic significance of this character (but see [57, 58]).

Similar to the situation in *Cetonia aurata* [13], the high color variation of the *Potosia cuprea* species complex (clade 4–6) was largely incongruent with the mtDNA topology, although most of the clades contained a more or less characteristic (or geographically stable) phenotype. Therefore, other mechanisms may be involved in maintaining color polymorphism. Beside the Mendelian (genetic) polymorphism (e.g. [83, 84]), environmental conditions (e.g. temperature or latitude [85, 86]) or biotic factors (e.g. mimicry and aposematism [87]) may often account for phenotypic plasticity in beetles. This can result in a complex multilevel regulation of color polymorphism [88, 89]. Iridescent coloration of numerous beetle groups including flower chafers has led to several possible biological explanations. Davis et al. [86] demonstrated that some dung beetles might exhibit a clinal coloration variability across longitudinal gradient owing for a thermal explanation of the phenomena. Other hypotheses encompass crypsis or predator blinding [90, 91], intra and inter specific signaling [92, 93] often in combination with the reflection of circularly polarized or UV light [94, 95], or even neutral evolution [96]. Henrotte et al. [97] found a correlation between substrate composition and cuticle thickness in *P*. *cuprea* and *C*. *aurata*. While an altitudinal gradient in coloration, setation or density of body punctation can be observed at least in some parts of the *P*. *cuprea* species complex range (e.g. France, Balkan peninsula etc.), some populations of *P*. *cuprea* (Apennine peninsula, Corsica and Sardinia, Levant) share similar coloration and morphological features (punctation coarseness, body shine and elytra coloration) with co-occurring *Cetonia* taxa [13]. Interestingly, while syntopic individuals of *C*. *aurata pisana* from the Italian mainland exhibit an unusually high genetic distance (ca 9% in *Cox1*), their coloration pattern (e.g. the bright coloration, vitreous or silky shine and microsculpture of dorsal body surface) remained the same and much resembling to syntopic populations of *P*. *cuprea*.

## Conclusion

Our results based on the integrative approach combining several different methods confirm the alleged polyphyly of *Potosia cuprea* and allowed three different types of taxonomic scenarios for the group as well as provide an initial preview in the phylogeography of this complex species group. Additionally, we found no support for the proposed classification of *P*. *cuprea metallica* as a separate species, as well for most of the traditionally recognized subspecies of *Protaetia* (*Potosia*) *cuprea*. The obtained tree topology reflects (with a single exception) geographical distribution of the taxa allowing discussions about an eastern Mediterranean/ponto-mediterranean origin of *P*. *cuprea* species complex. Moreover, we found that, with the exception of Sicily, most of the island populations (including Cyprus) show relationship to the adjacent mainland populations, questioning the taxonomic status of these populations. Most of the observed taxa-defining morphological characters were to variable for unambiguous taxonomic discrimination of the hypothesized evolutionary lineages. Thus, none of the proposed recent or past taxonomies were in congruence with mitochondrial data and the results of geometric morphometry.

Additional nuclear markers, more dense sampling and accumulation of other missing *P*. *cuprea* taxa especially from eastern parts of Western Palearctic will be crucial in further investigations, and will allow tests of possible gene flow within this highly variable and widespread group.

## Supporting information

S1 Table Taxonomy of *Potosia cuprea* species complex—Historical overview. A complex overview including information about distribution for each taxa (see shortcut list at the end of the file).
(DOCX)

S2 Table List of samples used in this study with exact localities and GenBank accession numbers. (XLSX)

S3 Table Character frequency per clade. The number of clades in which a given character was uniform (i.e. only one character stage per in a clade) as well as the number of stable characters per a clade.
(XLSX)

S1 Text Characters and their states. (DOCX)

S2 Text Character matrix. (DOCX)

S1 Fig Character explanation. Additional explanation of several characters and outlines for geometric morphometrics (green lines).
(PDF)

S2 Fig Character visualization using Mesquite software. All 29 characters mapped separately on the phylogenetic tree from Fig 1. Legend as well as consistency (CI) and retention (RI) index values included in each tree.
(PDF)

S3 Fig DCA plots from outline analysis. (PDF)
