# Supplementary material for: Phylogeography and DNA-based species delimitation provide insight into the taxonomy of the polymorphic rose chafer Protaetia (Potosia) cuprea species complex (Coleoptera: Scarabaeidae: Cetoniinae) in the Western Palearctic
Source: PLoS One. 2018 Feb 20;13(2):e0192349. doi: 10.1371/journal.pone.0192349 (PMC5819786; doi:10.1371/journal.pone.0192349)
Supplement: S1 Table — A complex overview including information about distribution for each taxa (see shortcut list at the end of the file). (DOCX) [file pone.0192349.s001.docx]

**S1 Table: Main taxa historical attributed to *Potosia cuprea* species complex with their respective taxonomical treatment in the most important works.**

| **Taxon**  **(Bezděk 2016)** | **Medvedev**  **1964** | **Mikšić**  **1987** | **Baraud**  **1992** | **Smetana**  **2006** | **Krajčík**  **2012^e^** | **Tauzin**  **2015** | **Distribution**  **(Bezděk 2016)** |
| --- | --- | --- | --- | --- | --- | --- | --- |
| *P. bessarabica*  Panin, 1942^a^ | + | *P. cuprea cuprina* | *P. cuprina* | nomina dubia | - | NA | **E**: MC |
| *P. caucasica*  (Kolenati, 1846) | + | *P. cuprea caucasica* | + | + | *P. cuprea caucasica* | NA | **E**: AB AR **A**: IN TR |
| *P. cuprea alainilerestifi*  Montreuil & Legrand, 2010 | NA | NA | NA | NA | + | NA | **A**: IN |
| *P. cuprea bicolorata*  Petrovitz, 1959 | - | - | - | - | + | - | **E**: GR (Lindos, Rhodos) |
| ***P. cuprea bourgini***  **Ruter, 1967** | **NA** | **+** | **+** | ***P. metallica bourgini*** | **+** | **+** | **E: BE FR GE LU** |
| ***P. cuprea brancoi***  **Baraud, 1992** | **NA** | **NA** | **+** | ***P. metallica brancoi*** | **+** | **+** | **E: PT SP N: CI** |
| ***P. cuprea cuprea***  **(Fabricius, 1775)** | **+** | **+** | **+** | **+** | **+** | **+** | **E: FR (Corse) IT SZ** |
| *P. cuprea daurica*  (Motschulsky, 1860) | *P. metallica daurica* | + | NA | + | + | NA | **A**: ES FE HEB HEI |
| *P. cuprea ferreriesensis*  Compte & Carreras, 2013^b^ | NA | NA | NA | NA | NA | NA | **E:** SP (Menorca) |
| *P. cuprea hesperica*  (Motschulsky, 1849)^c^ | NA | - | - | - | + | + | **E**: SP |
| ***P. cuprea ignicollis***  **(Gory & Percheron, 1833)** | **+** | **+** | **NA** | **+** | **+** | **NA** | **N: EG A: IQ IS LE SAi SI SY TR** |
| ***P. cuprea ikonomovi***  **Mikšić, 1958** | ***P. cuprina ikonomovi*** | **+** | **NA** | ***P. ikonomovi*** | ***P. ikonomovi*** | **NA** | **A: CY** |
| *P. cuprea levantina*  Schatzmayr, 1938 | NA | - | NA | + | + | NA | **E**: GR (Aegean Islands) |
| *P. cuprea mandli*  Balthasar, 1930 | - | - | NA | + | + | NA | **A**: ES |
| *P. cuprea mehrabii*  Montreuil & Legrand, 2008 | NA | NA | NA | NA | + | NA | **A**: IN |
| ***P. cuprea metallica***  **(Herbst, 1782)** | ***P. metallica*** | **+** | **+** | ***P. metallica*** | **+** | ***P. metallica*** | **E: AU BE BY CR CZ DE EN FI FR GB GE HU IT LA LT LU NL NR NT PL RO SB SK SL ST SV SZ UK A: ES WS** |
| ***P. cuprea obscura***  **(Andersch, 1797)** | **+** | **+** | **+** | **+** | **+** | **+** | **E: AL AU BH BU CR CZ GR HU IT KO MC ME RO SB SK SL SZ** |
| ***P. cuprea olivacea***  **(Mulsant, 1842)** | ***P. cuprea cuprea*** | **+** | **+** | **+** | **+** | **+** | **E: FR** |
| *P. cuprea phoebe*  Reitter, 1899^a^ | *P. cuprea ignicollis* | + | NA | + | + | NA | **A**: SY TR |
| *P. cuprea viridiaurata*  Fuente, 1897^c^ | NA | - | - | - | - | + | E: SP |
| **Taxon**  **(Bezděk 2016)** | **Medvedev**  **1964** | **Mikšić**  **1987** | **Baraud**  **1992** | **Smetana**  **2006** | **Krajčík**  **2012^e^** | **Tauzin**  **2015** | **Distribution**  **(Bezděk 2016)** |
| ***P. cuprea volhyniensis***  **(Gory & Percheron, 1833)** | ***P. metallica volhyniensis*** | **-** | **-** | **+** | **+** | **NA** | **E: CT ST UK A: KZ** |
| ***P. cuprina***  **(Motschulsky, 1849)** | **+** | ***P. cuprea cuprina*** | **+** | **+** | ***P. cuprea cuprina*** | **NA** | **E: AB AR BU GR MD RO ST TR UK A: TR** |
| *P. cuprina transfuga*  (Schaufuss, 1882)^a^ | *P. cuprina* | *P. cuprea cuprina* | - | + | - | NA | **E**: GR TR **A**: TR |
| *P. fausti*  (Kraatz, 1891) | + | *P. cuprea splendidula* | + | + | + | NA | **E**: AR GG **A**: TR |
| *P. h. hieroglyphica*  (Ménétriés, 1832) | + | *P. cuprea hieroglyphica* | + | + | *P. cuprea hieroglyphica* | NA | **E**: AB GG ST **A**: IN TM |
| *P. h. depressiuscula*  Reitter, 1891 | + | *P. cuprea hieroglyphica* | NA | + | *P. cuprea depressiuscula* | NA | **A**: IN TM |
| ***P. hypocrita***  **Ragusa, 1905** | ***P. cuprea obscura*** | ***P. cuprea incerta*** | ***P. cuprea incerta*** | ***P. incerta*** | ***P. cuprea hypocrita*** | **+** | **E: IT (Sicilia) MA** |
| *P. indica*  Mikšić, 1965 | NA | *P. cuprea indica* | NA | + | *P. cuprea indica* | NA | **A**: AF HP KA PA |
| *P. marginicollis*  (Ballion, 1871) | + | + | NA | + | + | NA | **A**: AF KI KZ TD UZ XIN |
| *P. mayeti*  Le Comte, 1906 | NA | - | - | + | *P. cuprea mayeti* | NA | **E**: MA **N**: LB |
| *P. multifoveolata*  Reitter, 1899 | + | + | NA | + | + | NA | **A**: GAN HUN SCH XIZ |
| *P. neglecta*  (Hope, 1831) | - | +^d^ | NA | + | + | NA | **A**: AF AP HP KA NP PA SD UP XIZ **ORR** |
| *P. nitididorsis*  (Fairmaire, 1889) | NA | *P. cuprea nitididorsis* (?) | NA | + | + | NA | **A**: BEI CHQ FE GUI GUX HEI |
| *P. splendidula*  (Faldermann, 1835) | + | *P. cuprea splendidula* | + | + | + | NA | **E**: AB AR **A**: IQ SY TR |

Taxa in bold are represent in this study. The list of shortcuts used for countries and other areas are in S1 Table. Explanation: “+” taxon cited in the respective study, “-” taxon absent in the respective study, “NA” taxon not listed in the respective study, either its range is outside the scope of the work, or it was not described at the time of the publication.

^a^Taxa which are listed in the new edition of Catalogue of Palaearctic Coleoptera (Bezděk 2016) as synonyms, but they were somehow historically connected with *cuprea* complex in main publications and we think that they should be listed here:

Krajčík (1998) listed *P. cuprina transfuga* (Schaufuss, 1882). Name is currently synonym of *P. cuprina* (Bezděk 2016).

*Potosia bessarabica* Panin, 1942 is listed only by Medvedev (1964). In other publications is mentioned as a synonym of *P. cuprina* or *P. cuprina transfuga*.

*Potosia cuprea phoebe* Reitter, 1899 is mentioned in Bezděk 2016 as synonym of *P. cuprea ignicollis*.

^b^*Potosia cuprea ferreriesensis* Compte & Carreras, 2013 was not mentioned in Tauzin 2015 and Bezděk 2016.

^c^These taxa are listed in Bezděk 2016, however we consider them as dubious and irrelevant for the purpose of our study.

^d^In the introduction of *P. cuprea* chapter in Mikšić (1987) the author note that this species might be a member of the “*cuprea* Gruppe”, based on the shape of parameres. However, he treated the taxa as a separate species.

^e^In Krajčík (2012) all mentioned *Potosia* taxa are incorporated in subgenus *Netocia* Costa, 1852. Also he listed *P. cuprea hypocrita* and *P. cuprea incerta* both as valid subspecies.

|  | **S1: List of shortcuts used in Table 1.** | | |
| --- | --- | --- | --- |
| **E** | **Europe** | **A** | **Asia** |
| **AB** | Azerbaijan | **AF** | Afghanistan |
| **AL** | Albania | **AP** | India: Arunachal Pradesh |
| **AR** | Armenia | **BEI** | Beijing |
| **AU** | Austria | **CHQ** | Chongqing |
| **BE** | Belgium | **CY** | Cyprus |
| **BH** | Bosnia Herzegovina | **ES** | Russia: East Siberia |
| **BU** | Bulgaria | **FE** | Russia: Far East |
| **BY** | Belarus | **GAN** | Gansu |
| **CR** | Croatia | **GUI** | Guizhou |
| **CT** | Russia: Central European Territory | **GUX** | Guangxi |
| **CZ** | Czech Republic | **HEB** | Hebei |
| **DE** | Denmark | **HEI** | Heilongjiang |
| **EN** | Estonia | **HP** | India: Himachal Pradesh |
| **FI** | Finland | **HUN** | Hunan |
| **FR** | France (incl. Corsica, Monaco) | **IN** | Iran |
| **GB** | Great Britain (incl. Channel Islands) | **IQ** | Iraq |
| **GE** | Germany | **IS** | Israel |
| **GG** | Georgia | **KA** | India: Kashmir |
| **GR** | Greece (incl. Crete) | **KI** | Kyrgyzstan |
| **HU** | Hungary | **KZ** | Kazakhstan |
| **IT** | Italy (incl. Sicily, San Marino) | **LE** | Lebanon |
| **KO** | Kosovo | **NP** | Nepal |
| **LA** | Latvia | **PA** | Pakistan |
| **LT** | Lithuania | **SAi** | Saudi Arabia |
| **LU** | Luxembourg | **SCH** | Sichuan |
| **MA** | Malta | **SD** | India: Sikkim, Darjeeling District |
| **MC** | Macedonia | **SI** | Egypt: Sinai |
| **MD** | Moldova (Moldavia) | **SY** | Syria |
| **ME** | Montenegro | **TD** | Tajikistan |
| **NL** | The Netherlands | **TM** | Turkmenistan |
| **NR** | Norway | **TR** | Turkey |
| **NT** | Russia: North European Territory | **UP** | India: Uttarakhand (Uttaranchal, former part of Uttar Pradesh) |
| **PL** | Poland | **UZ** | Uzbekistan |
| **PT** | Portugal | **WS** | Russia: West Siberia |
| **RO** | Romania | **XIN** | Xinjiang |
| **SB** | Serbia | **XIZ** | Xizang |
| **SK** | Slovakia | **ORR** | Oriental Region |
| **SL** | Slovenia |  |  |
| **SP** | Spain (incl. Gibraltar) | **N** | **North Africa** |
| **ST** | Russia: South European Territory | **CI** | Canary Islands |
| **SV** | Sweden | **EG** | Egypt |
| **UK** | Switzerland | **LB** | Libya |
| **TR** | Turkey |  |  |
