# Supplementary material for: Phylogeography and DNA-based species delimitation provide insight into the taxonomy of the polymorphic rose chafer Protaetia (Potosia) cuprea species complex (Coleoptera: Scarabaeidae: Cetoniinae) in the Western Palearctic
Source: PLoS One. 2018 Feb 20;13(2):e0192349. doi: 10.1371/journal.pone.0192349 (PMC5819786; doi:10.1371/journal.pone.0192349)
Supplement: S1 Text — (DOCX) [file pone.0192349.s004.docx]

S1 Text: List of 29 characters morphological characters and their states, which were used in this study, along with their consistency and retention indexes. Refer to S3 for explanation of character 20 – 28 on meso-metaventral (MMV) protrusion.

**1. Shine of the dorsal body face (CI=0.150/ RI=0.528):**

0) matt 1) shining metallic 2) silky/vitreous 3) semi-shining.

**2. Ventral macrosetation (CI=0.080/ RI=0.228):**

0) absent/very faint 1) normally developed 2) dense.

**3. Pronotal sculpture (CI=0.133/ RI=0.519):**

0) coarse 1) fine 2) absent/glabrous.

**4. Elytral sculpture (humeral part) (CI=0.095/ RI=0.424):**

0) coarse 1) fine 2) absent/glabrous.

**5. Elytral sculpture (distal part) (CI=0.143/ RI=0.400):**

0) coarse 1) fine.

**6. Color of head (CI=0.304/ RI=0.579):**

0) green metallic 1) vivid green/vivid green with red, golden or blue shine 2) bronze with green touches 3) bronze with red touches 4) dark bronze with blue or violet touches or dark blue/violet with metallic and bronze touches 5) maroon/violet 6) vivid red/vivid red with green touches 7) black/black-green or black-blue.

**7. Color of legs (CI=0.279/ RI=0.579):**

0) green metallic 1) vivid green/vivid green with red, golden or blue shine 2) bronze with green touches 3) bronze with red touches 4) dark bronze with blue or violet touches or dark blue/violet with metallic and bronze touches 5) maroon/violet 6) black/black-green or black-blue.

**8. Color of mesepimeron (CI=0.300/ RI=0.641):**

0) green metallic 1) vivid green/vivid green with red, golden or blue shine 2) bronze with green touches 3) bronze with red touches 4) dark bronze with blue or violet touches or dark blue/violet with metallic and bronze touches 5) maroon/violet 6) black/black-green or black-blue.

**9. Color of pronotum (CI=0.286/ RI=0.625):**

0) green metallic 1) vivid green/vivid green with red, golden or blue shine 2) bronze with green touches 3) bronze with red touches 4) maroon/violet 5) vivid red/vivid red with green touches 6) black/black-green or black-blue.

**10. Color of pronotal emargination (CI=0.333/ RI=0.650):**

0) green metallic 1) vivid green/vivid green with red, golden or blue shine 2) bronze with green touches 3) bronze with red touches 4) dark bronze with blue or violet touches or dark blue/violet with metallic and bronze touches 5) maroon/violet 6) vivid red/vivid red with green touches 7) black/black-green or black-blue.

**11. Color of scutellum (CI=0.286/ RI=0.625):**

0) green metallic 1) vivid green/vivid green with red, golden or blue shine 2) bronze with green touches 3)bronze with red touches 4) maroon/violet 5) vivid red/vivid red with green touches 6) black/black-green or black-blue.

**12. Color of elytra (CI=0.238/ RI=0.590):**

0) green metallic 1) vivid green/vivid green with red, golden or blue shine 2) bronze with green touches 3) bronze with red touches 4) maroon/violet 5) black/black-green or black-blue.

**13. Color of pygidium (CI=0.292/ RI=0.575):**

0) green metallic 1) vivid green/vivid green with red, golden or blue shine 2) bronze with green touches 3) bronze with red touches 4) dark bronze with blue or violet touches or dark blue/violet with metallic and bronze touches 5) maroon/violet 6) vivid red/vivid red with green touches 7) black/black-green or black-blue.

**14. Color of ventral body part (CI=0.333/ RI=0.636):**

0) green metallic 1) vivid green/vivid green with red, golden or blue shine 2) bronze with green touches 3) bronze with red touches 4) dark bronze with blue or violet touches or dark blue/violet with metallic and bronze touches 5) maroon/violet 6) black/black-green or black-blue.

**15. White markings on elytra (CI=0.120/ RI=0.333):**

0) absent 1) faint 2) normally developed 3) accentuated.

**16. White markings on pronotum (excl. marginal parts) (CI=0.231/ RI=0,167):**

0) absent 1) faint, only one pair of paramedial discal markings 2) faint, two or three pairs of paramedial discal spots + occasionally other spots 3) three pairs of paramedial discal markings and other white cretaceous spots on pronotal disk.

**17. White markings on abdominal ventrites (CI=0.111/ RI=0.273):**

0) absent 1) only one or more small spots at the lateral ends of each sternite 2) lateral spots and mediolateral band on each side of at least sternite 3) only paramedial white bands.

**18. White markings on pronotal margin (CI=0.105/ RI=0.261):**

0) without any white marking 1) with single white spot or with several white spots 2) with an entire or broken white line.

**19. most proximal part of meso- and metatibiae with (or absent from) whitish patches laterally (CI=0.125/ RI=0.741):**

0) absent 1) present.

**20. MMV protrusion: apical margin (CI=0.095/ RI=0.240)**:

0) straight 1) slightly convex 2) convex.

**21. MMV protrusion: lateral margin (CI=0.143/ RI=0.333):**

0) straight 1) slightly convex 2) with angle or strongly convex.

**22. MMV protrusion: punctation density (CI=0.083/ RI=0.290):**

0) low 1) intermediate 2) high.

**23. MMV protrusion: punctation coarseness (CI=0.111/ RI=0.200):**

0) low 1) intermediate 2) high.

**24. Visibility of MMV suture (CI=0.095/ RI=0.240):**

0) indistinct 1) low 2) high.

**25. Angle of MMV suture (CI=0.118/ RI=0.343):**

0) with acute or right angle 1) with obtuse angle 2) almost straight.

**26. MMV protrusion: surrounding macrosetation (CI=0.074/ RI=0.138):**

0) absent/very faint 1) normally developed 2) dense.

**27. Separation of MMV suture and the suture of the emargination of mesocoxal cavity (CI=0.111/ RI=0.273):**

0) with at least a single contact point 1) sutures separated, distance between both lines is smaller than the average thickness of the emargination of mesocoxal cavity 2) sutures separated, distance between both lines is equal or thicker as the average thickness of the emaragination of mesocoxal cavity.

**28. Metasternum: Emargination of the mesocoxal cavity (CI=0.111/ RI=0.385):**

0) with an entire suture reaching to the midline suture of mesosternal disc 1) suture not completely developed at one or both sides, however its direction towards the midline suture of the metasternal disc well indicated 2) suture of the emargination indistinct or absent proximity to the midline suture of mesosternal disc.

**29. Reflection of lefthanded polarized light (CI=1/ RI=1):**

0) absent 1) present.
