# Supplementary material for: Phylogeography and DNA-based species delimitation provide insight into the taxonomy of the polymorphic rose chafer Protaetia (Potosia) cuprea species complex (Coleoptera: Scarabaeidae: Cetoniinae) in the Western Palearctic
Source: PLoS One. 2018 Feb 20;13(2):e0192349. doi: 10.1371/journal.pone.0192349 (PMC5819786; doi:10.1371/journal.pone.0192349)
Supplement: S2 Text — (DOCX) [file pone.0192349.s005.docx]

**S2 Text: Matrix of 29 characters for 65 *Potosia* samples and *Cetonia aurata* as outgroup used in further analyses in Mesquite software:**

MATRIX

'005CU_TR' ?????????????????????????????

'006Ob_GR' 30000555050055000011131111111

'007PF_GR' 10110333333333100001110110111

'008PF_CZ' 10110333333333100001110111101

'014CU_TR' 11110443333334332211120111111

'015Me_CZ' 32000444000044121111031102101

'016Ob_CZ' 31100555050025000010031111111

'017PF_CZ' 10110333333333101001131201101

'018PF_CZ' 10110333333333100001130110111

'019Ob_GR' 31010555050055003011120112111

'020Ob_GR' 310105550500550030111200?2111

'021PF_GR' 10110333333333100002220101011

'022PF_GR' 10110333333333101000110112101

'023Ob_GR' 11000555222255001010221112101

'024Ob_GR' 11000555252255102110110211111

'025Th_GR' 11000442222244221210022201101

'026Th_GR' 11000552222225101010130112011

'027PH_IT' 10110555555555000001030202211

'029PH_IT' 10110555555555000002030102211

'032Ob_HR' ?????????????????????????????

'033Ob_HR' 30110555050025000011230101111

'034Br_PT' 12110232222224202111010202001

'035Br_PT' 31000242222242000111120102111

'037Br_ES' 30000222222244101110010100211

'038Ob_GR' 10000444000044102111130101111

'039Ob_GR' 30000444222244102111130111111

'040Ig_JO' 21221611666161002002130110011

'041Ig_JO' 21121611666161000002120201001

'058Ig_JO' 20221111111111000001130210011

'059Pc_IT' 21121555151155000011120211111

'060PA_HR' 30100000505500000000031201001

'061PA_GR' 30100000000000000000020201011

'063PA_GR' 31100000203300000000031202011

'064Ob_GR' 30110555050055000012120200211

'067Pc_IT' 20121555151155000001220111211

'068Me_CZ' 31000444000044221111132112101

'070Pc_FR' 20110555151155000001120111111

'071CA_SW' 11100033030033101002110122-21

'073Me_SW' 30000443333344111110132110101

'074CU_TR' 301002422222241211121300?1011

'076CU_TR' 11110443333334332212120211111

'077Me_HU' 11110444000044102011130111101

'078Bo_FR' 31110232222244201011120211111

'081Ig_LE' 2022161166605?000001130200111

'088Pc_FR' 20110555151155000011120111-21

'090Me_SK' 320004440000443321101320?2101

'091Br_ES' 12110232222244102110131112111

'095Br_PT' 12110232222244202211120111101

'096PF_GR' 11110333333333101001120111011

'097Pc_FR' 21120555151155100101120111111

'101Ig_LE' 20221611666061000001120100111

'103Ob_CZ' 31010555000025003011131112001

'104PO_MO' 01111777777777000000221201100

'105PO_MO' 01111777777777000001220201100

'106Pc_IT' 20111555151155000001130111211

'107Me_PL' 30000044040034231110032100001

'108Me_PL' 11000444000044332110122201001

'109Th_TR' 11000344242234101110131111001

'110Vo_UA' 320005440000343121101300?2001

'111Vo_UA' 30000040000034111111132111101

'112Vo_UA' 30000333333334132200131110211

'113Ol_FR' 300003553533342110112300?01?1

'114Bo_FR' 12011355252234312111121212101

'115Ik_CY' 12000222222222302201131212-21

'116PO_TU' 00110777777777000001220201100

'117PO_TU' 00110777777777000001221201000
