# Supplementary material for: Phylogeography and DNA-based species delimitation provide insight into the taxonomy of the polymorphic rose chafer Protaetia (Potosia) cuprea species complex (Coleoptera: Scarabaeidae: Cetoniinae) in the Western Palearctic
Source: PLoS One. 2018 Feb 20;13(2):e0192349. doi: 10.1371/journal.pone.0192349 (PMC5819786; doi:10.1371/journal.pone.0192349)

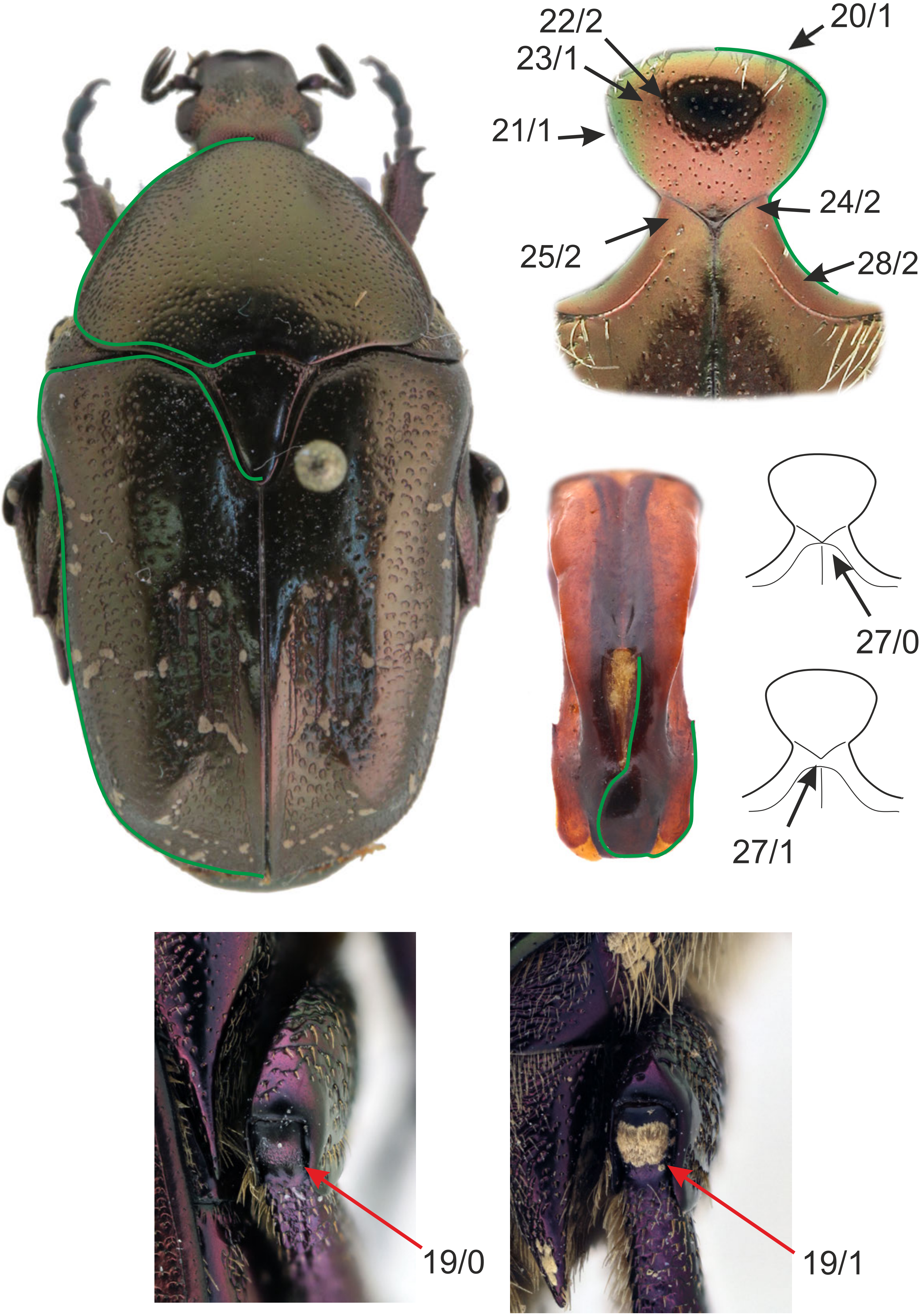

22/2
20/1
23/1
21/1
24/2
25/2
28/2
27/0
27/1
19/0
19/1

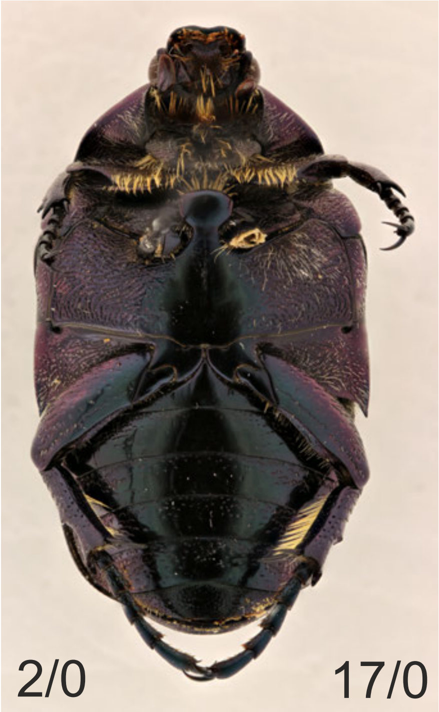

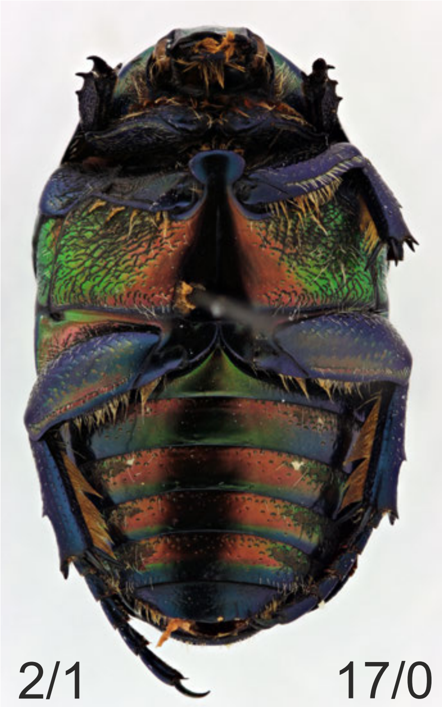

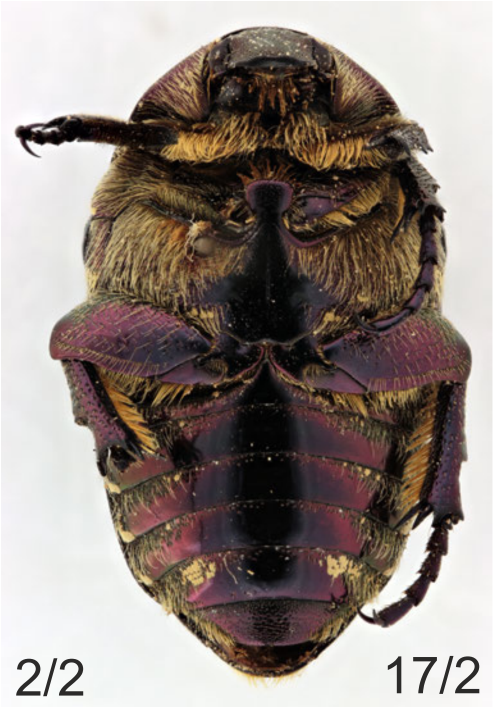

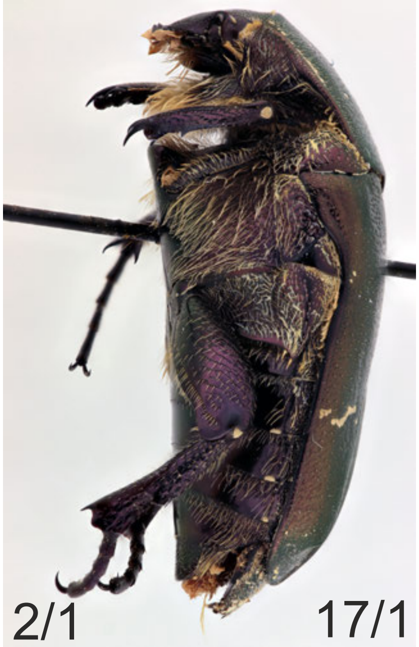

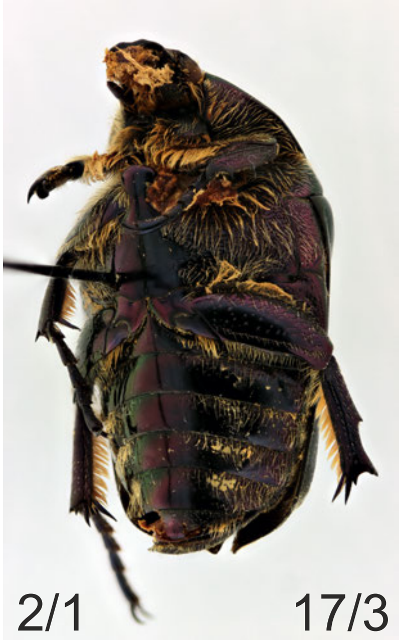

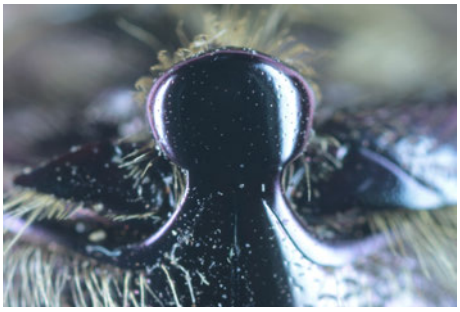

20/2, 21/1, 22/1, 23/0, 24/2
25/1, 26/1, 27/1, 28/1

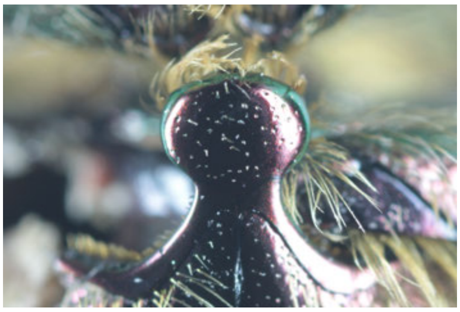

20/1, 21/1, 22/1, 23/1, 24/2
25/1, 26/2, 27/1, 28/0

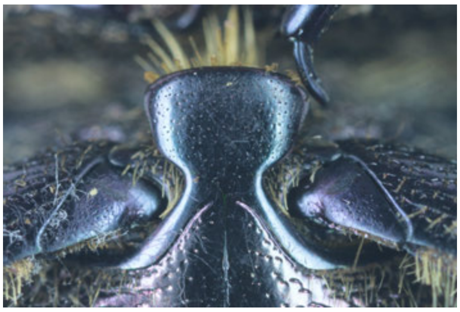

20/1, 21/1, 22/2, 23/0, 24/1
25/1, 26/1, 27/1, 28/1

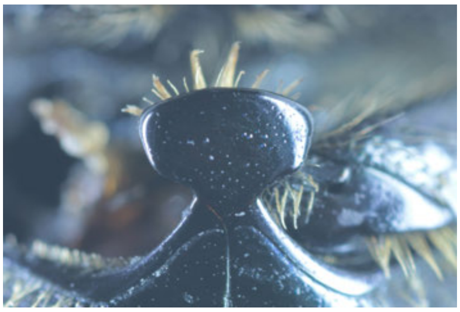

20/1, 21/2, 22/1, 23/0, 24/2
25/0, 26/1, 27/1, 28/0

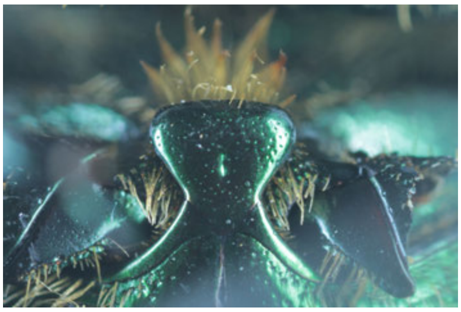

20/0, 21/0, 22/2, 23/1, 24/2
25/0, 26/1, 27/0, 28/0

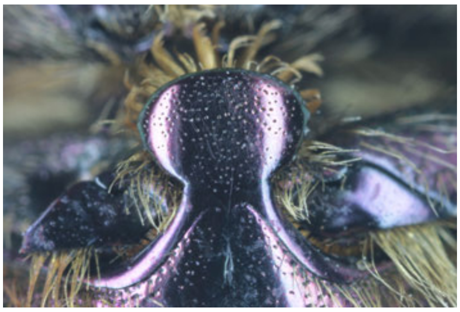

20/1, 21/1, 22/2, 23/1, 24/1
25/1, 26/2, 27/0, 28/0

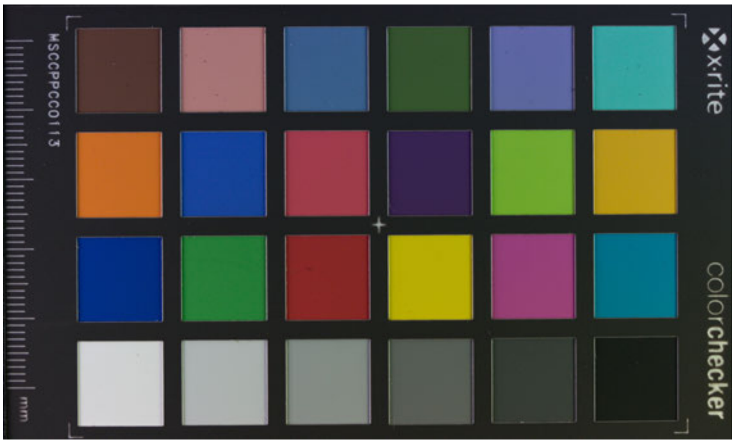

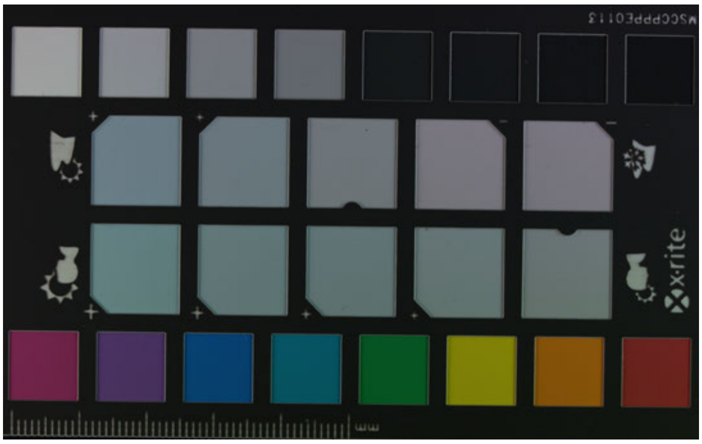

Potosia angustata
(063PA_GR)

1/3
3/1
4/0
5/0
6/0
7/0
8/0
9/2
10/0
11/3
12/3
15/0
16/0
18/0

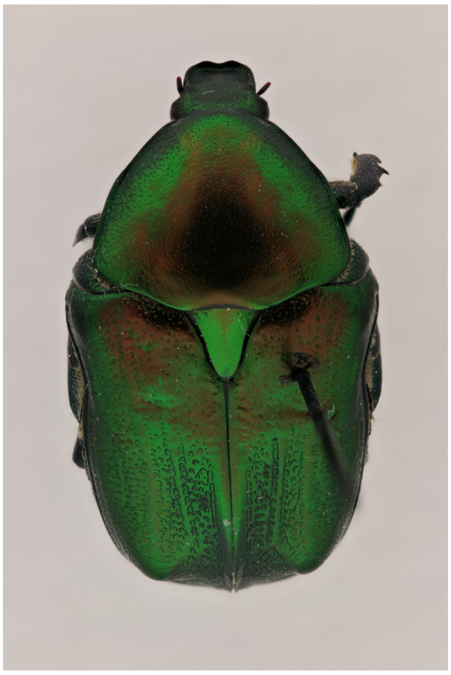

Potosia cuprina
(076CU_TR)

1/1
3/1
4/1
5/0
6/4
7/4
8/3
9/3
10/3
11/3
12/3
15/3
16/3
18/2

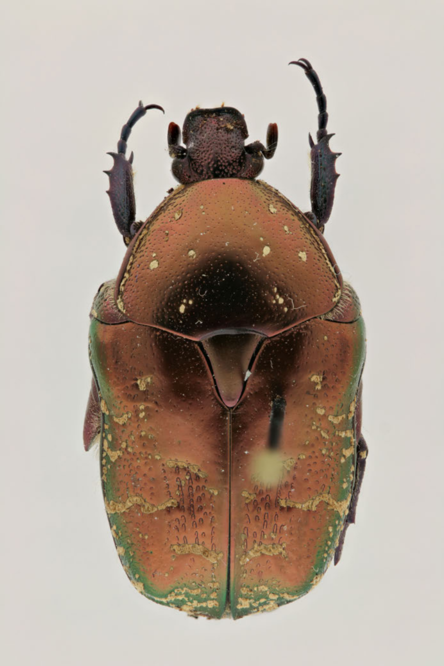

Potosia cuprea brancoi
(034Br_PT)

1/1
3/1
4/1
5/0
6/2
7/3
8/2
9/2
10/2
11/2
12/2
15/2
16/0
18/1

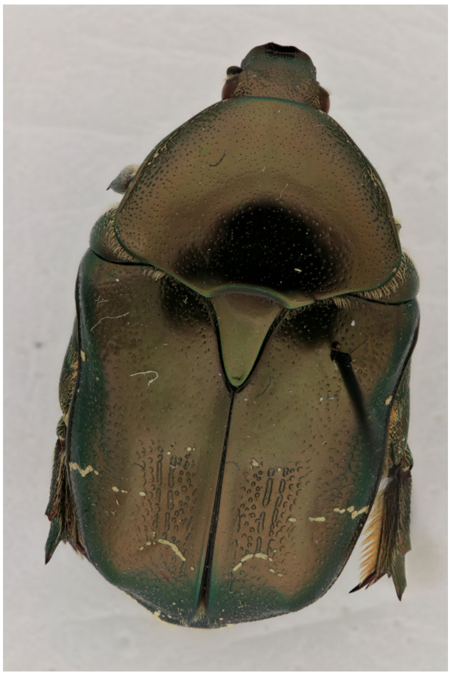

Potosia cuprea cuprea
(059Pc_PT)

1/2
3/1
4/2
5/1
6/5
7/5
8/5
9/1
10/5
11/1
12/1
15/0
16/0
18/0

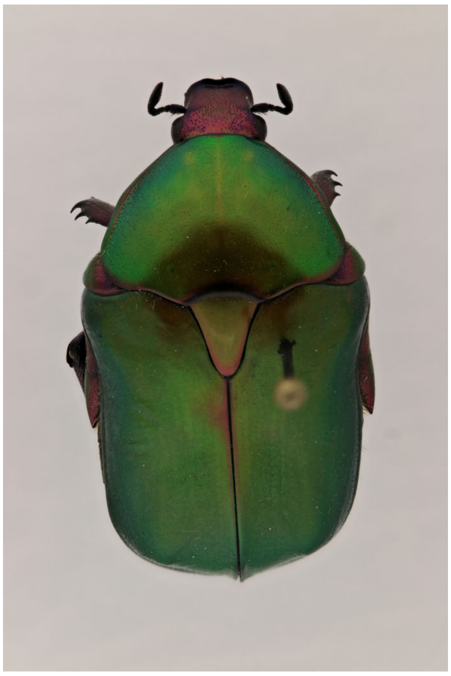

Potosia cuprea ignicollis
(041Ig_JO)

1/2
3/1
4/2
5/1
6/6
7/1
8/1
9/5
10/6
11/5
12/1
15/0
16/0
18/0

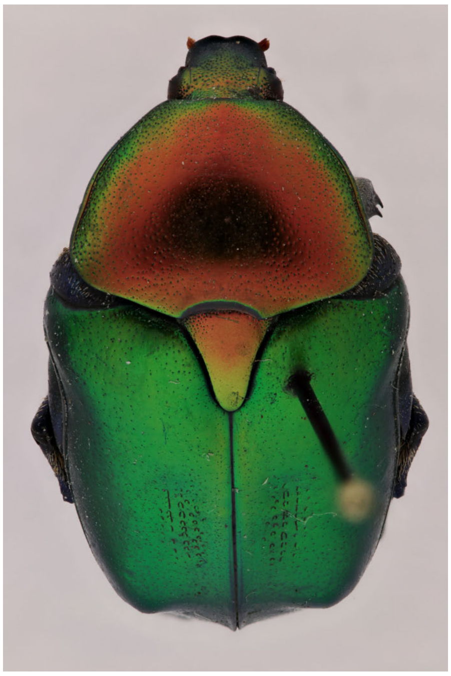

Potosia cuprea metallica
(077Me_HU)

1/1
3/1
4/1
5/0
6/4
7/4
8/4
9/0
10/0
11/0
12/0
15/1
16/0
18/0

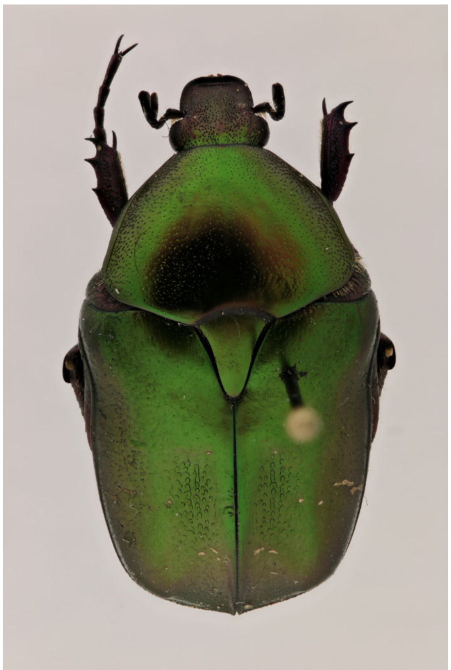

Potosia cuprea ignicollis
(058Ig_JO)

1/2
3/2
4/2
5/1
6/1
7/1
8/1
9/1
10/1
11/1
12/1
15/0
16/0
18/0

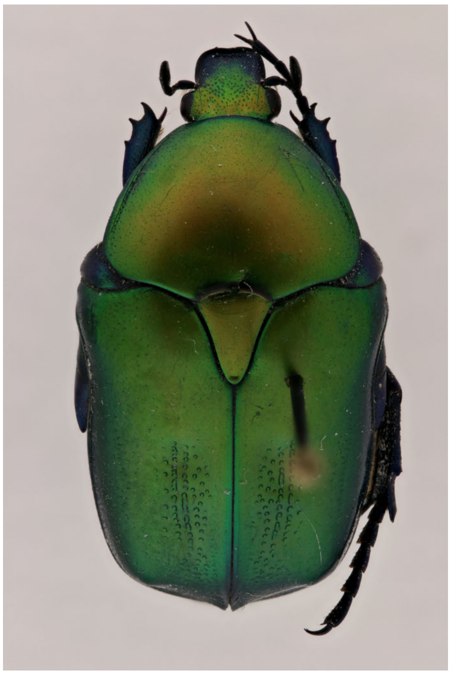

Potosia fieberi
(096PF_GR)

1/1
3/1
4/1
5/0
6/3
7/3
8/3
9/3
10/4
11/4
12/4
15/1
16/0
18/0

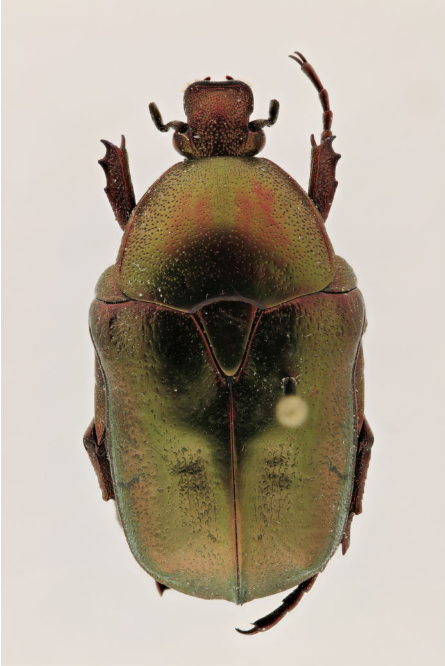

Potosia hypocrita
(027PH_IT)

1/1
3/1
4/1
5/0
6/5
7/5
8/5
9/4
10/5
11/4
12/4
15/0
16/0
18/0

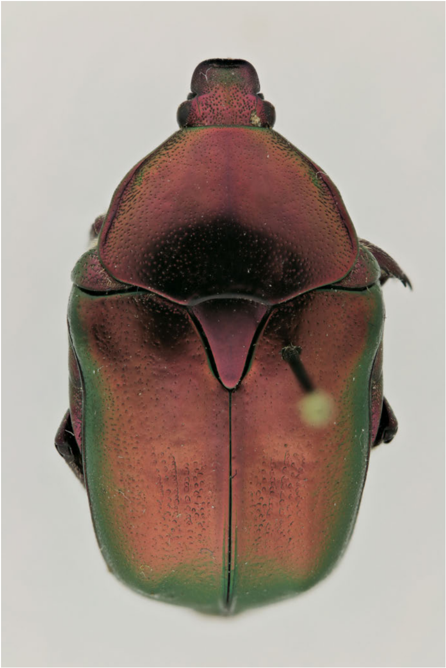

Potosia opaca
(117PO_TN)

1/0
3/1
4/1
5/0
6/7
7/6
8/6
9/6
10/7
11/6
12/5
15/0
16/0
18/0

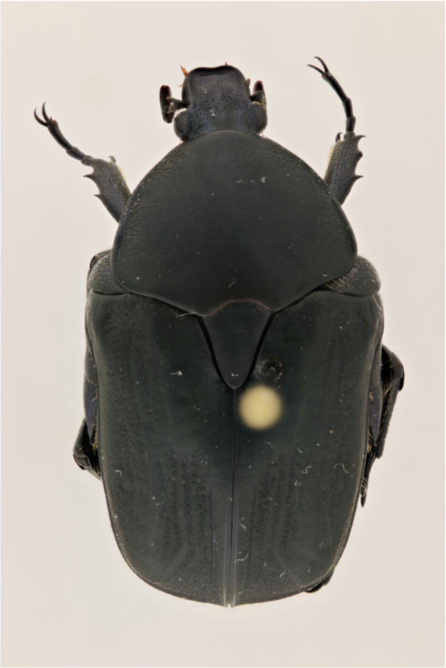

Supplement: S1 Fig — Additional explanation of several characters and outlines for geometric morphometrics (green lines). (PDF) [file pone.0192349.s006.pdf]
