# Supplementary figures and images for: Phylogeography and DNA-based species delimitation provide insight into the taxonomy of the polymorphic rose chafer Protaetia (Potosia) cuprea species complex (Coleoptera: Scarabaeidae: Cetoniinae) in the Western Palearctic
Source: PLoS One. 2018 Feb 20;13(2):e0192349. doi: 10.1371/journal.pone.0192349 (PMC5819786; doi:10.1371/journal.pone.0192349)

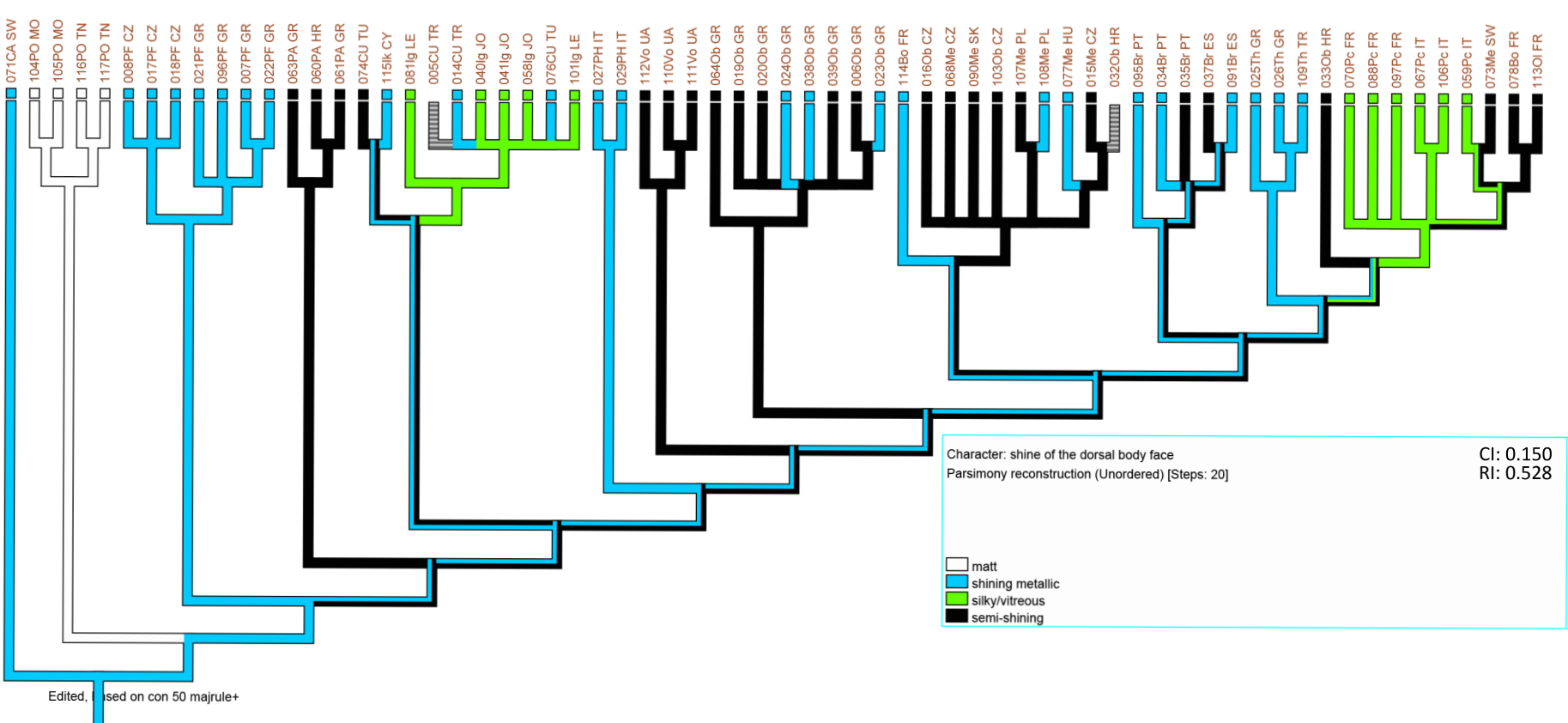

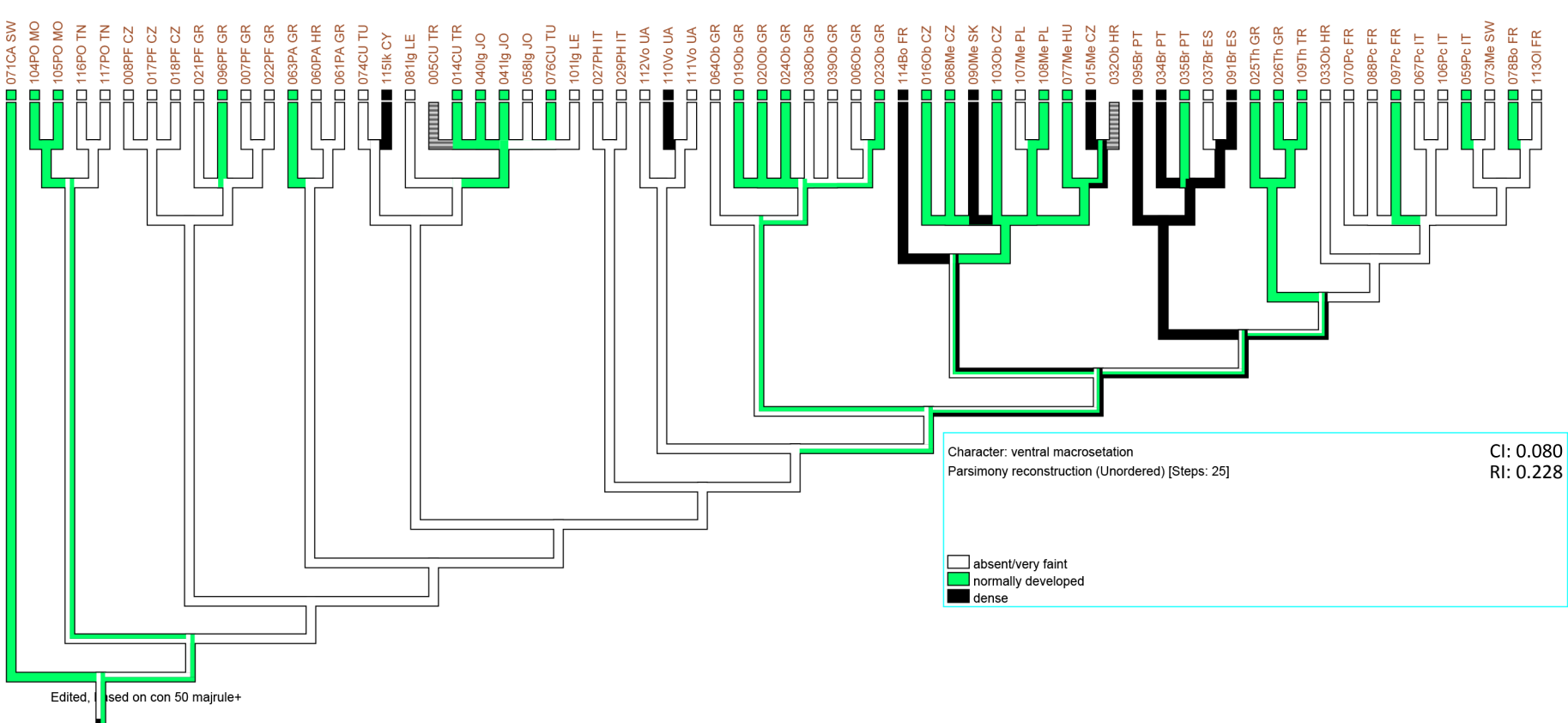

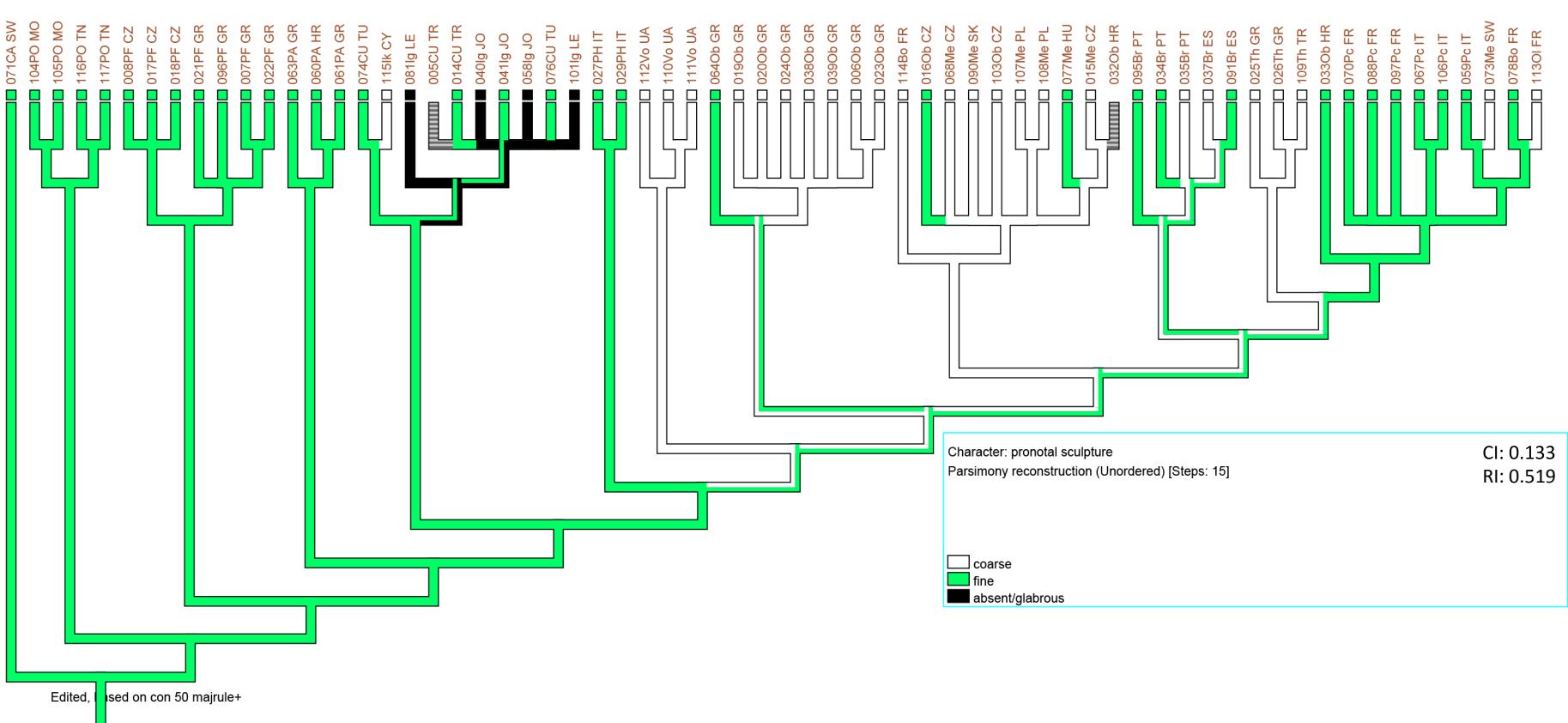

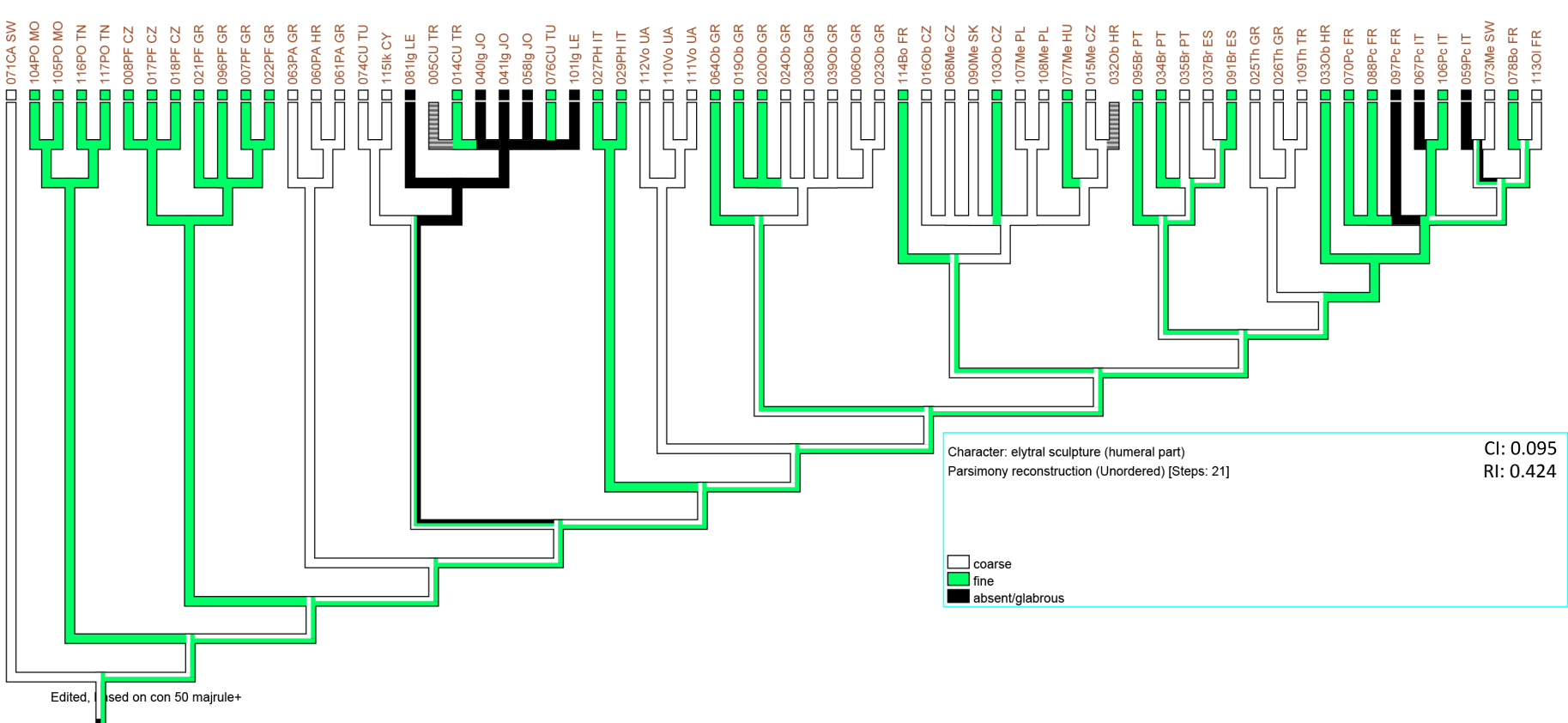

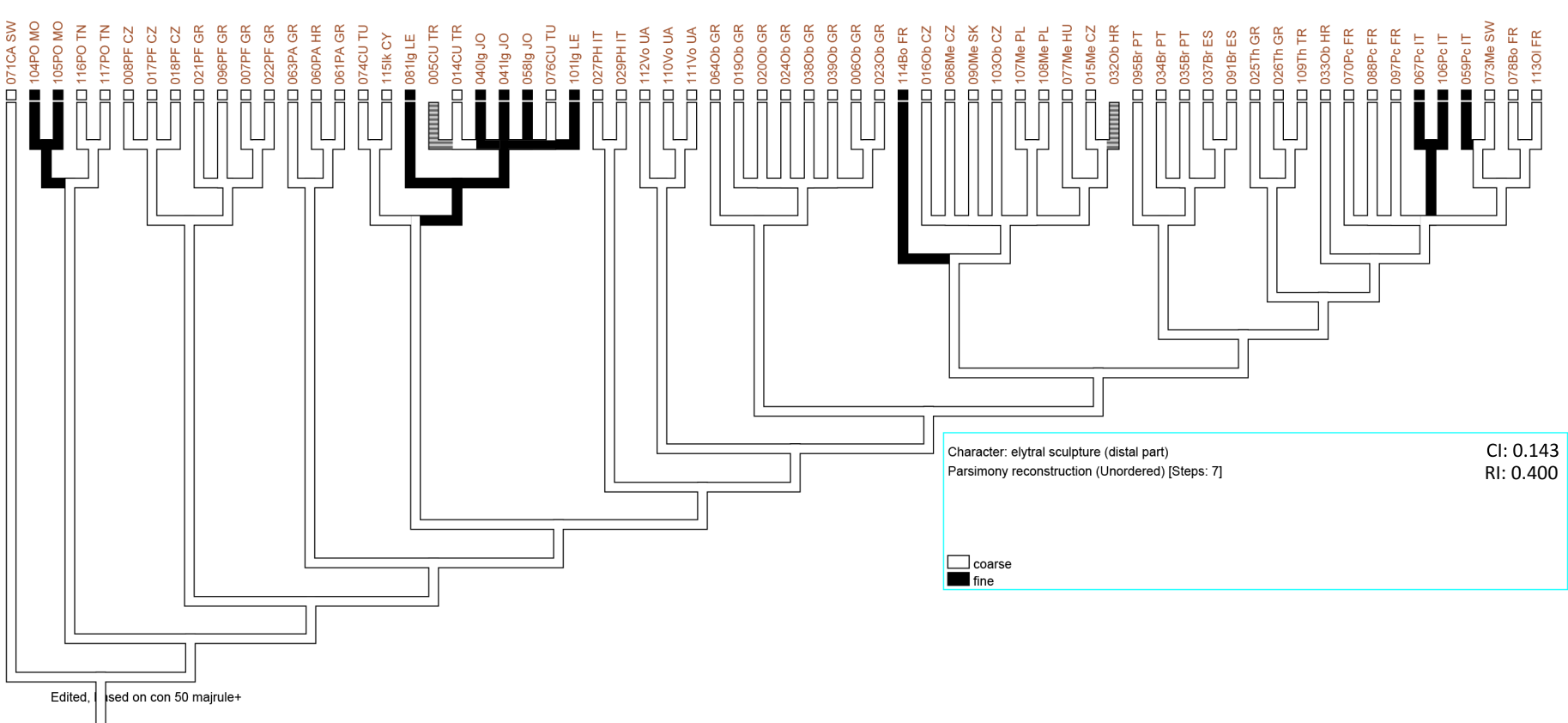

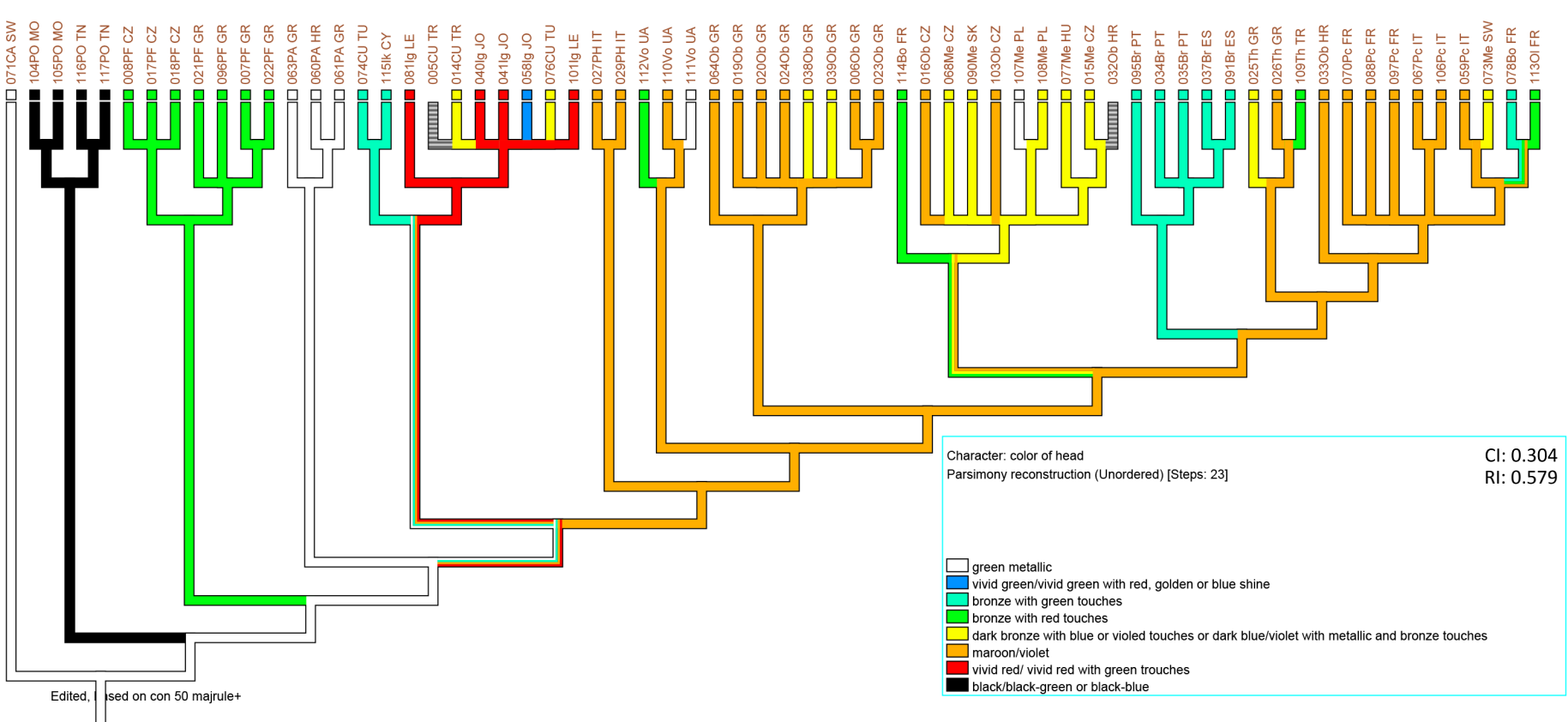

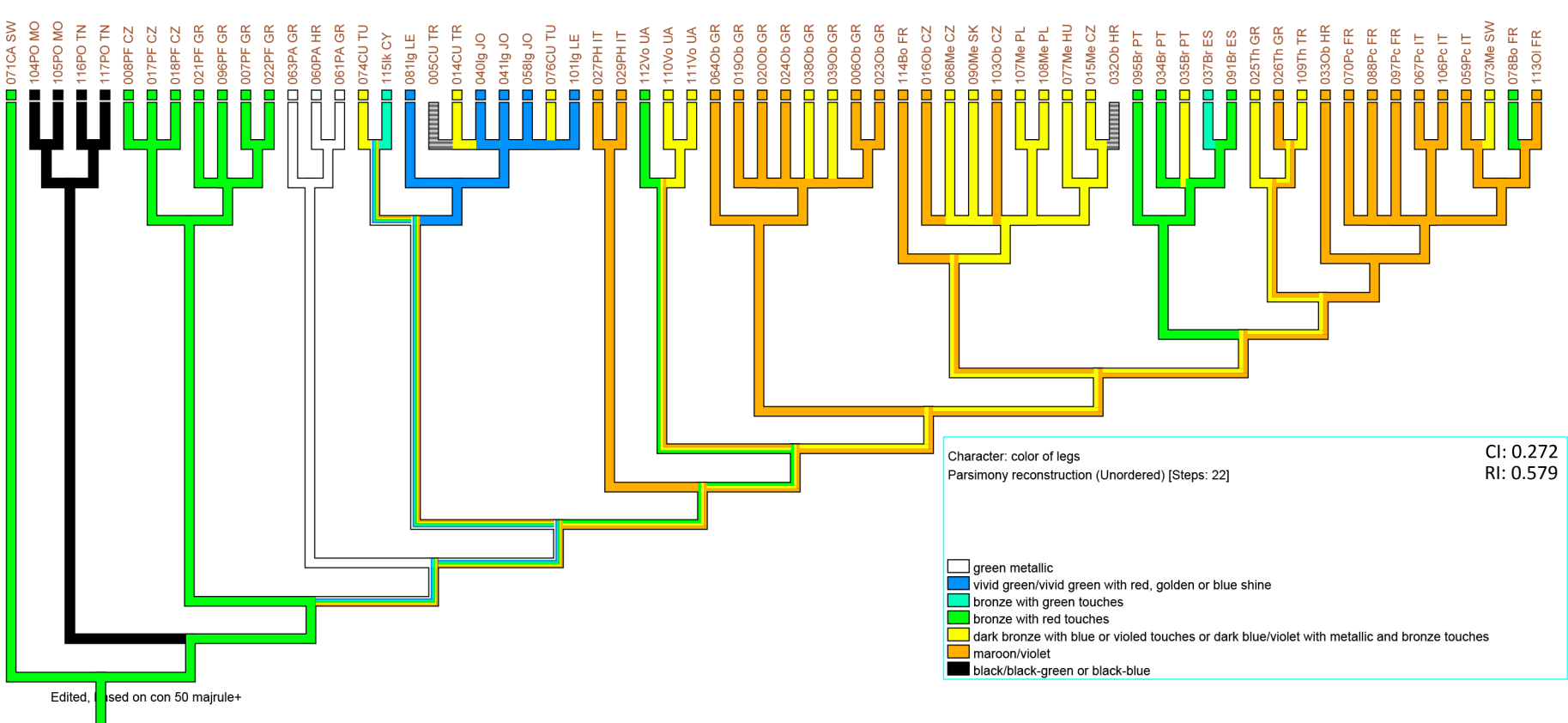

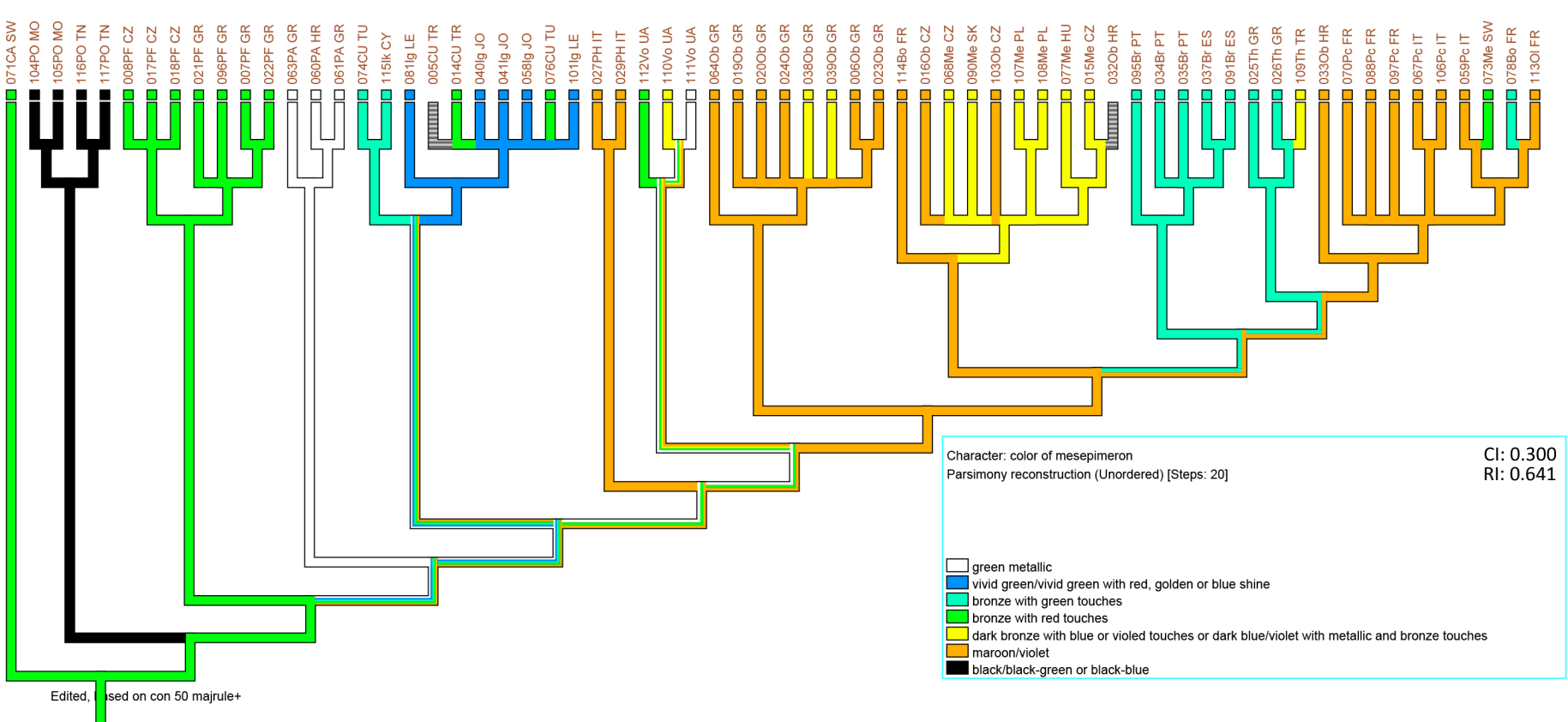

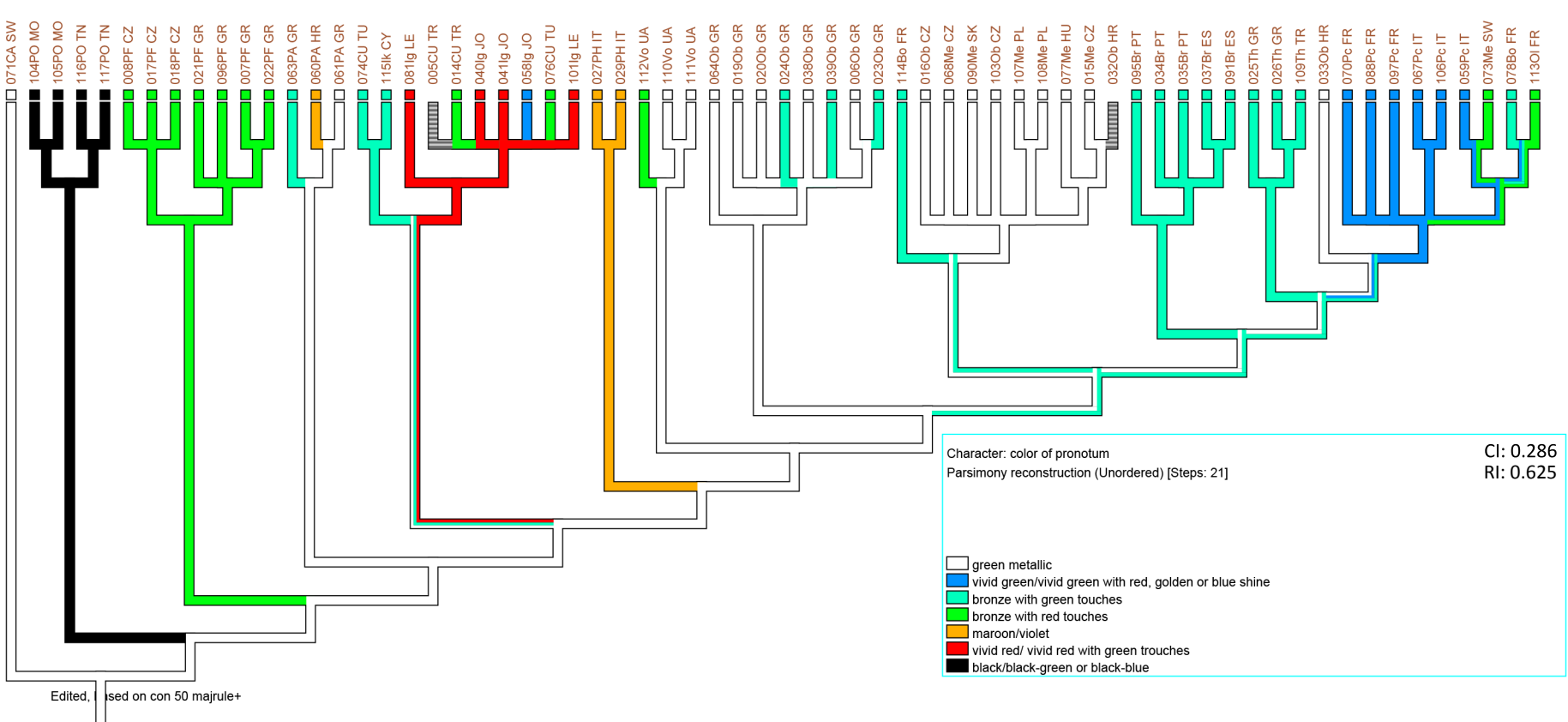



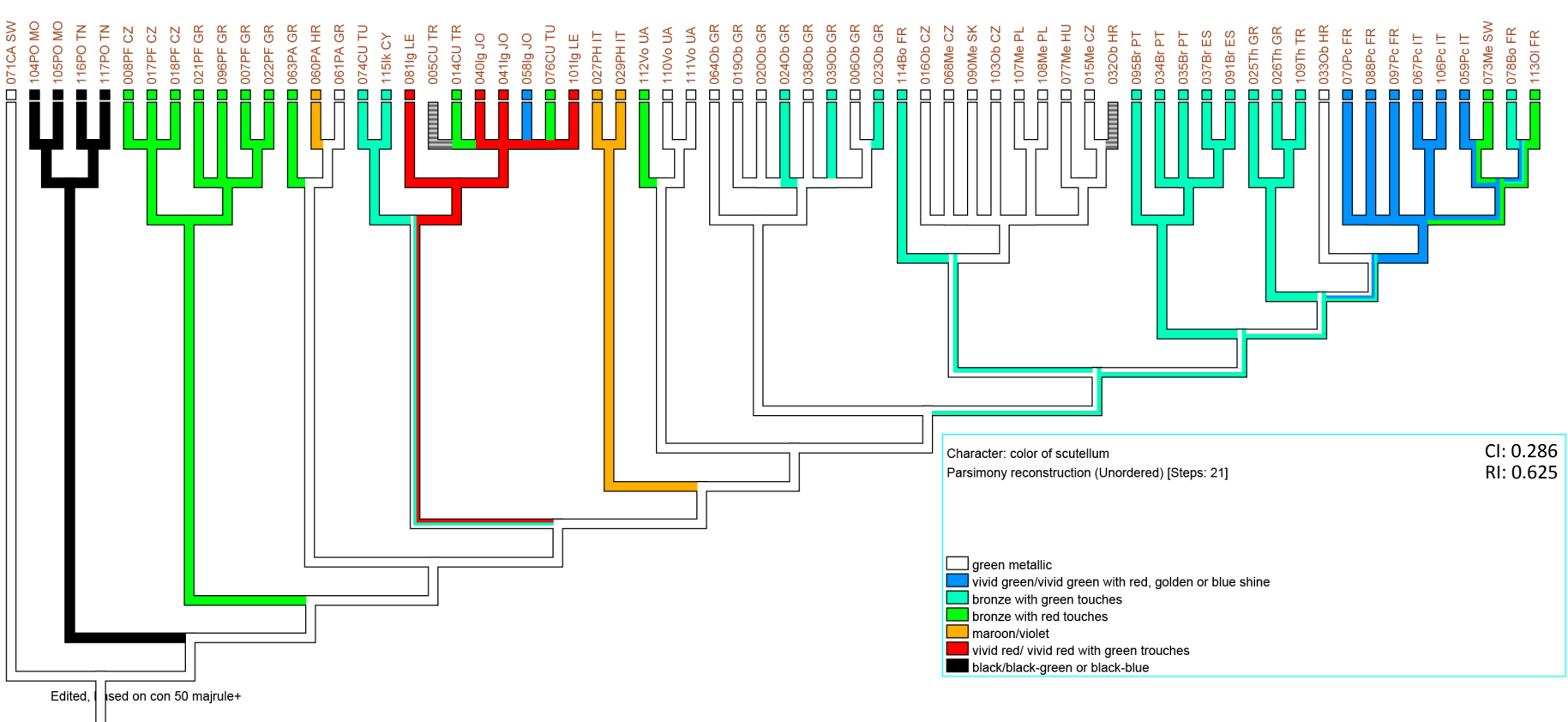

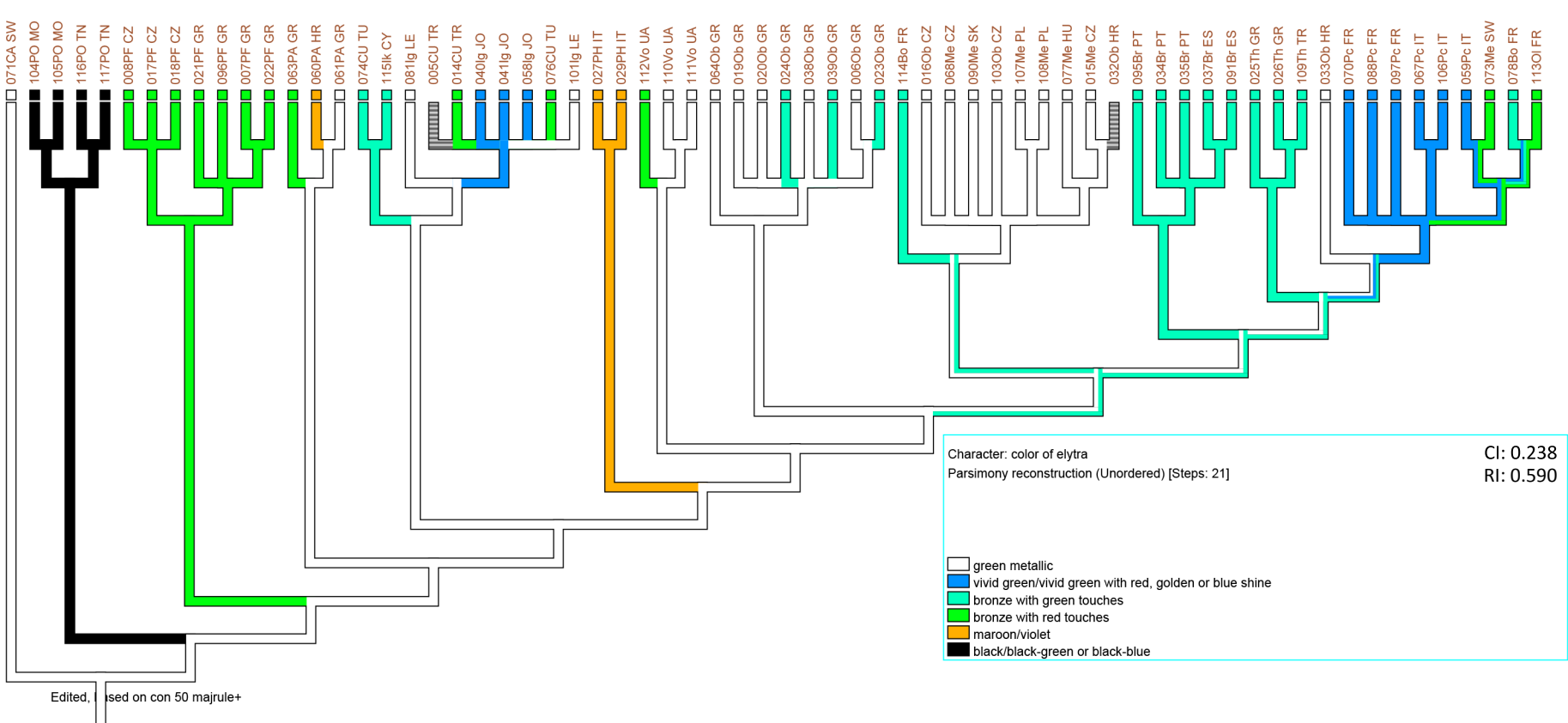

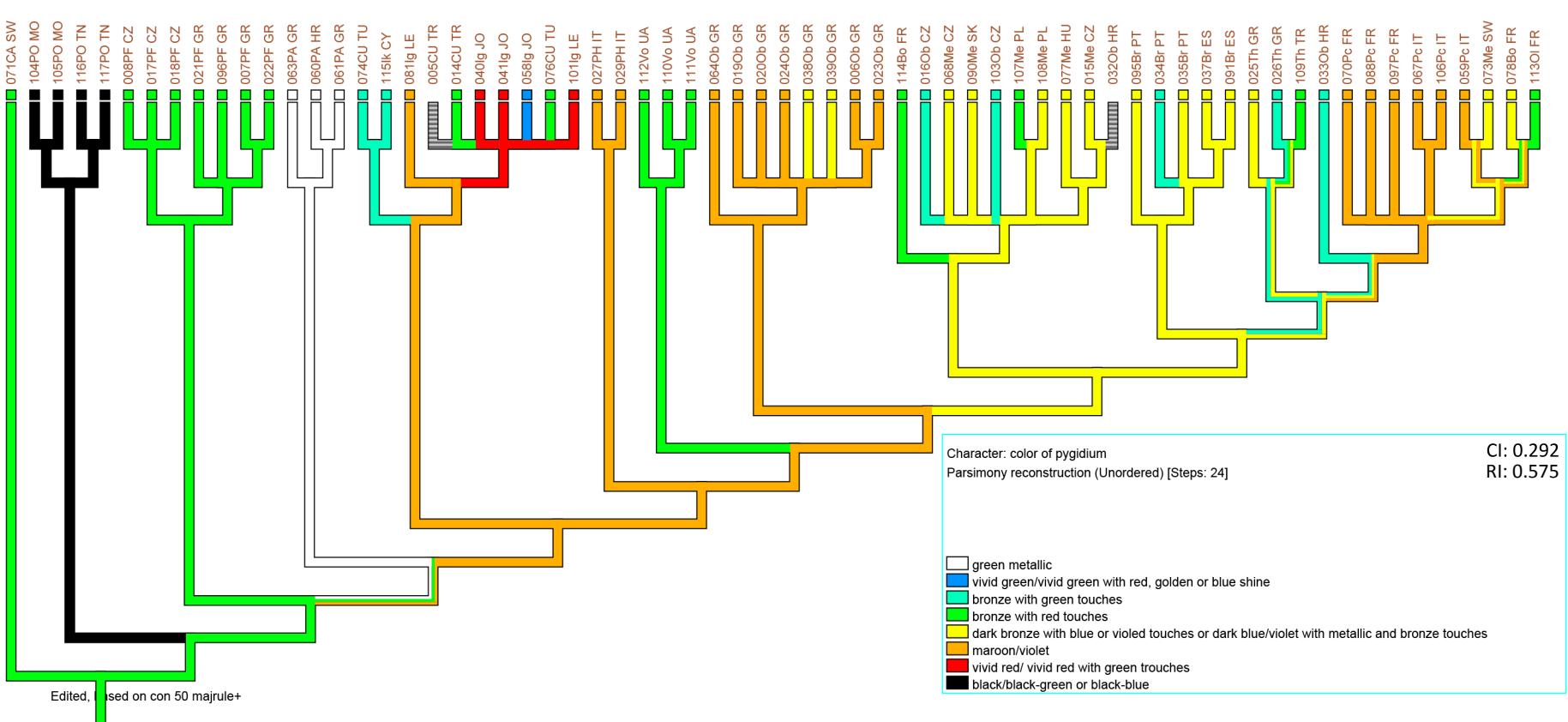



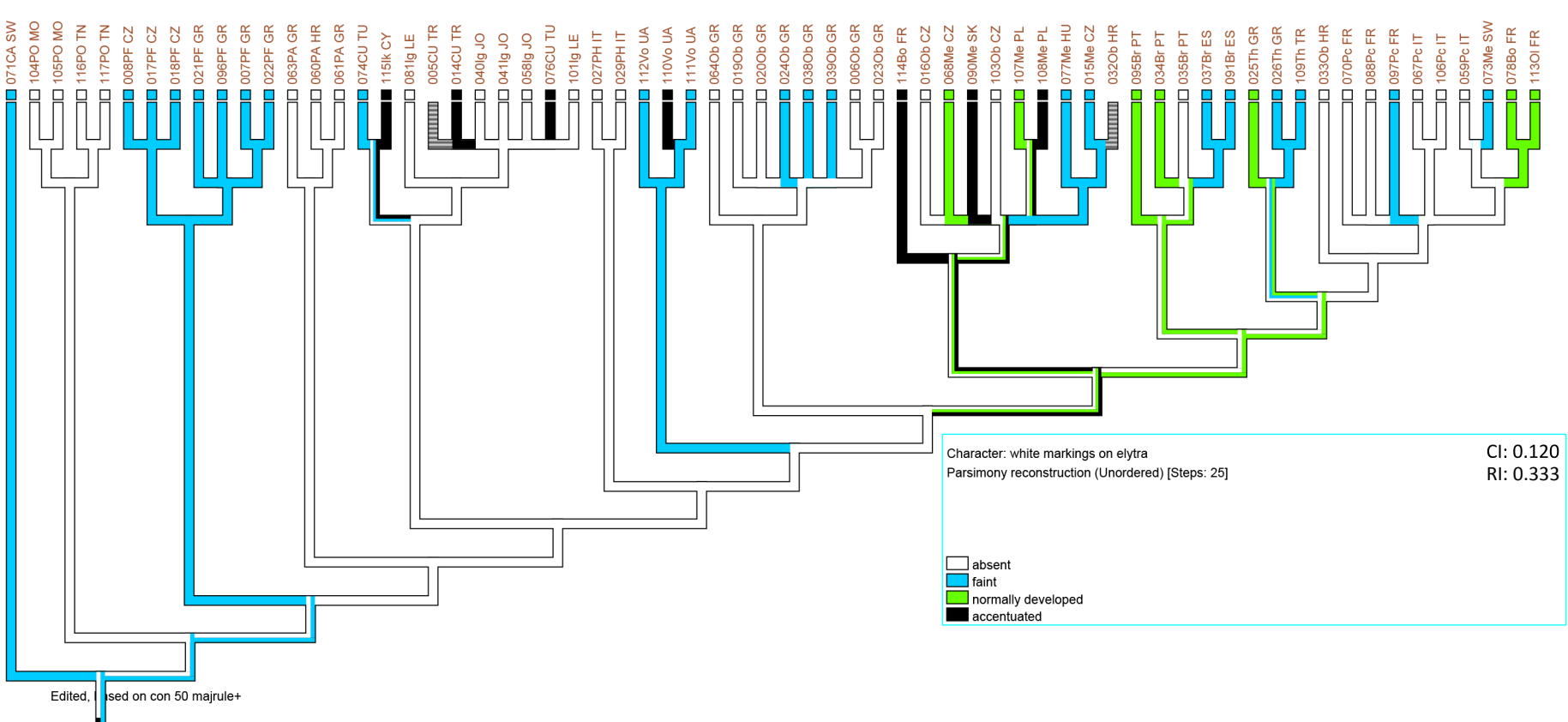



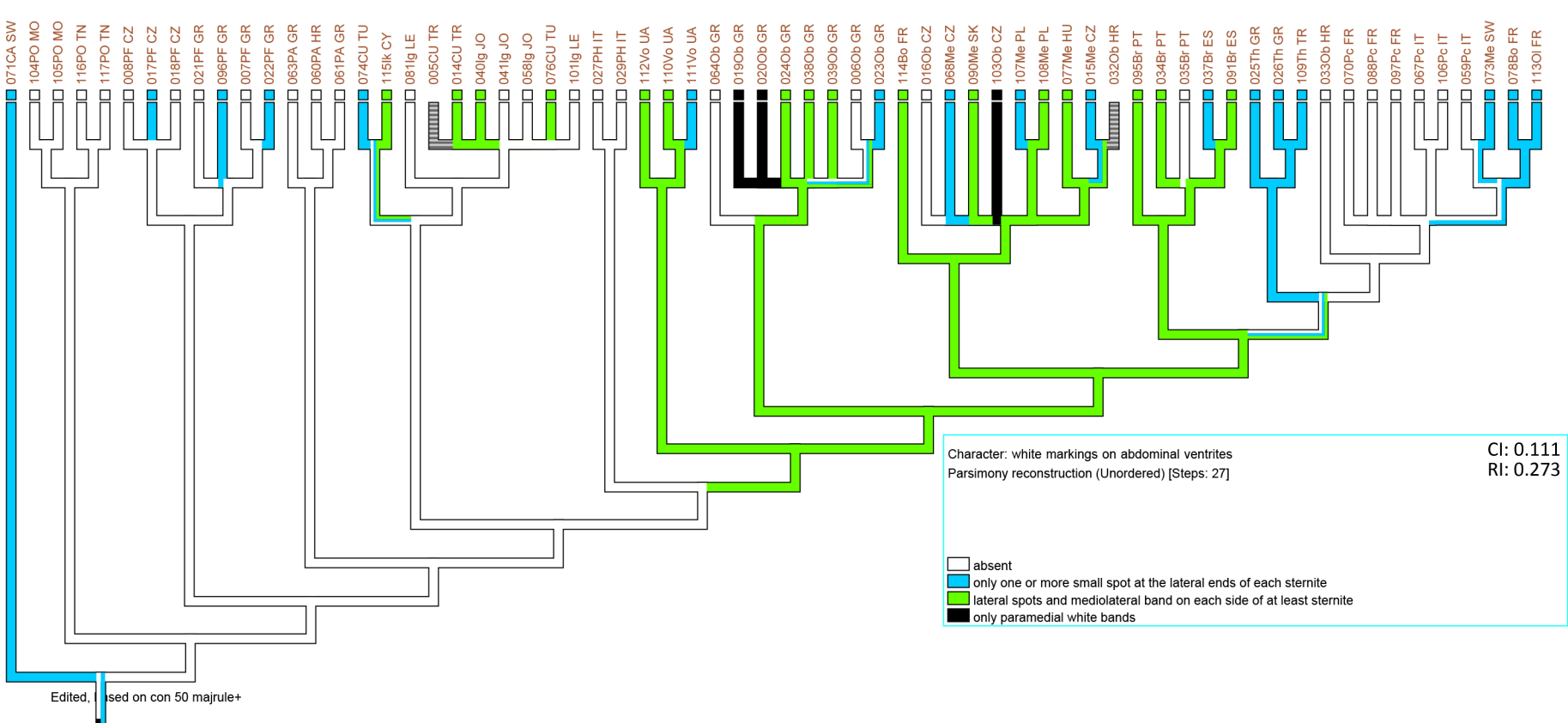

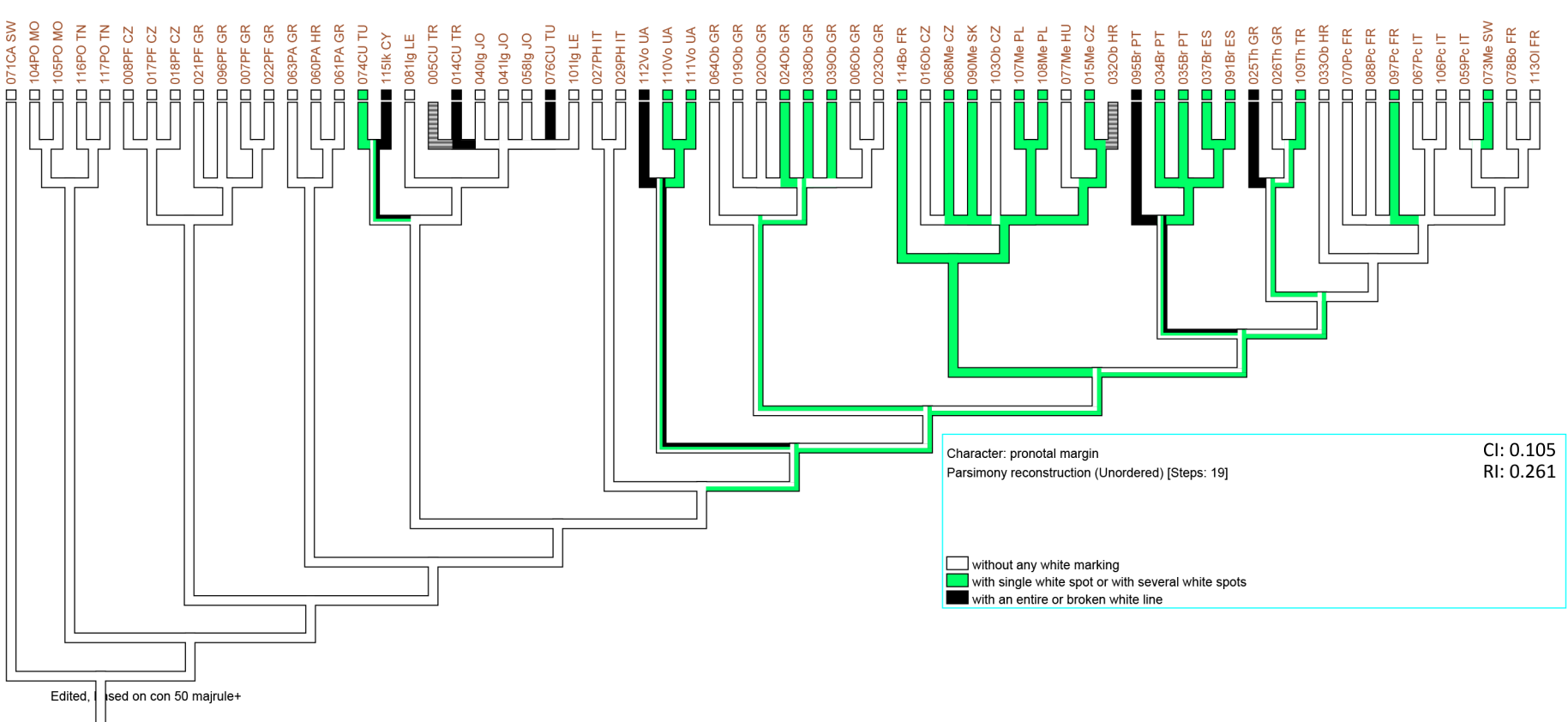

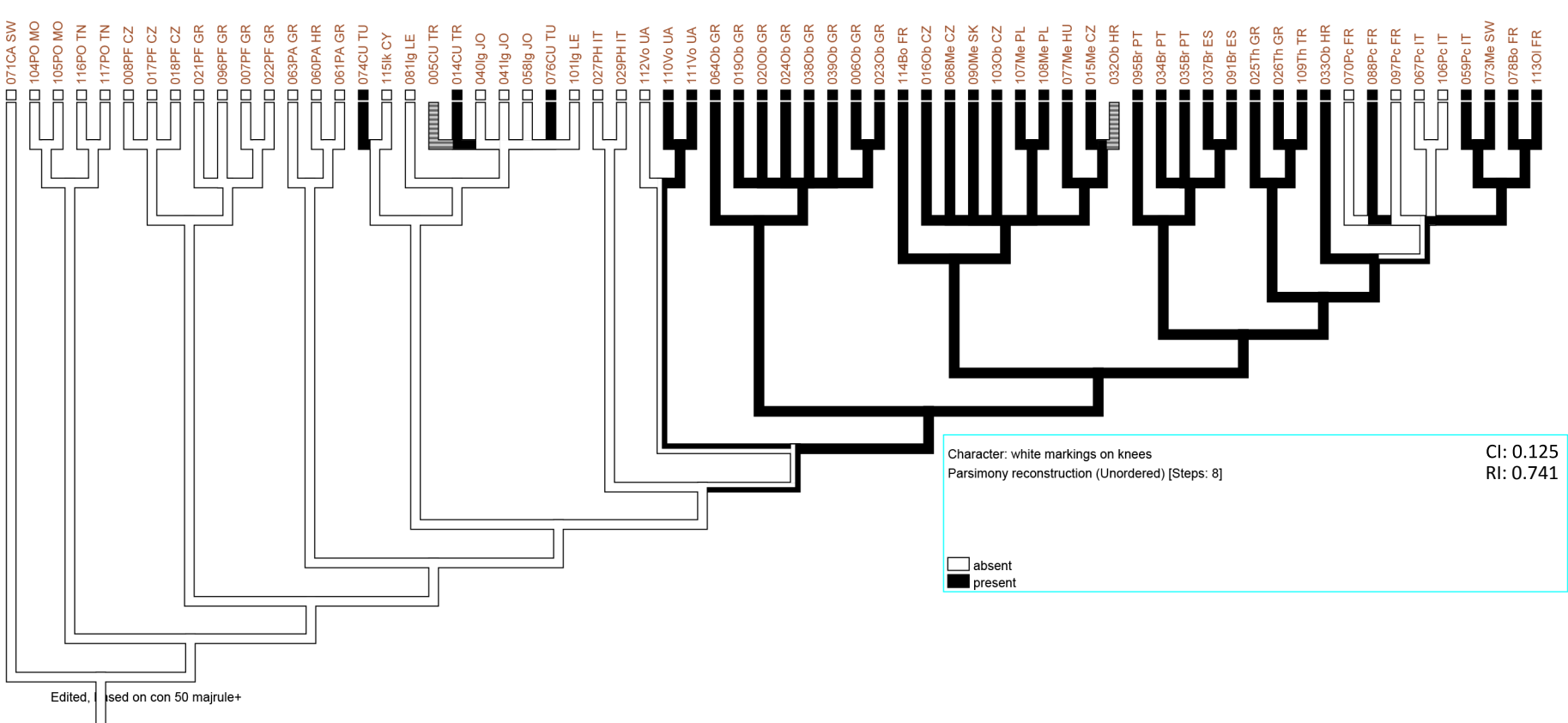

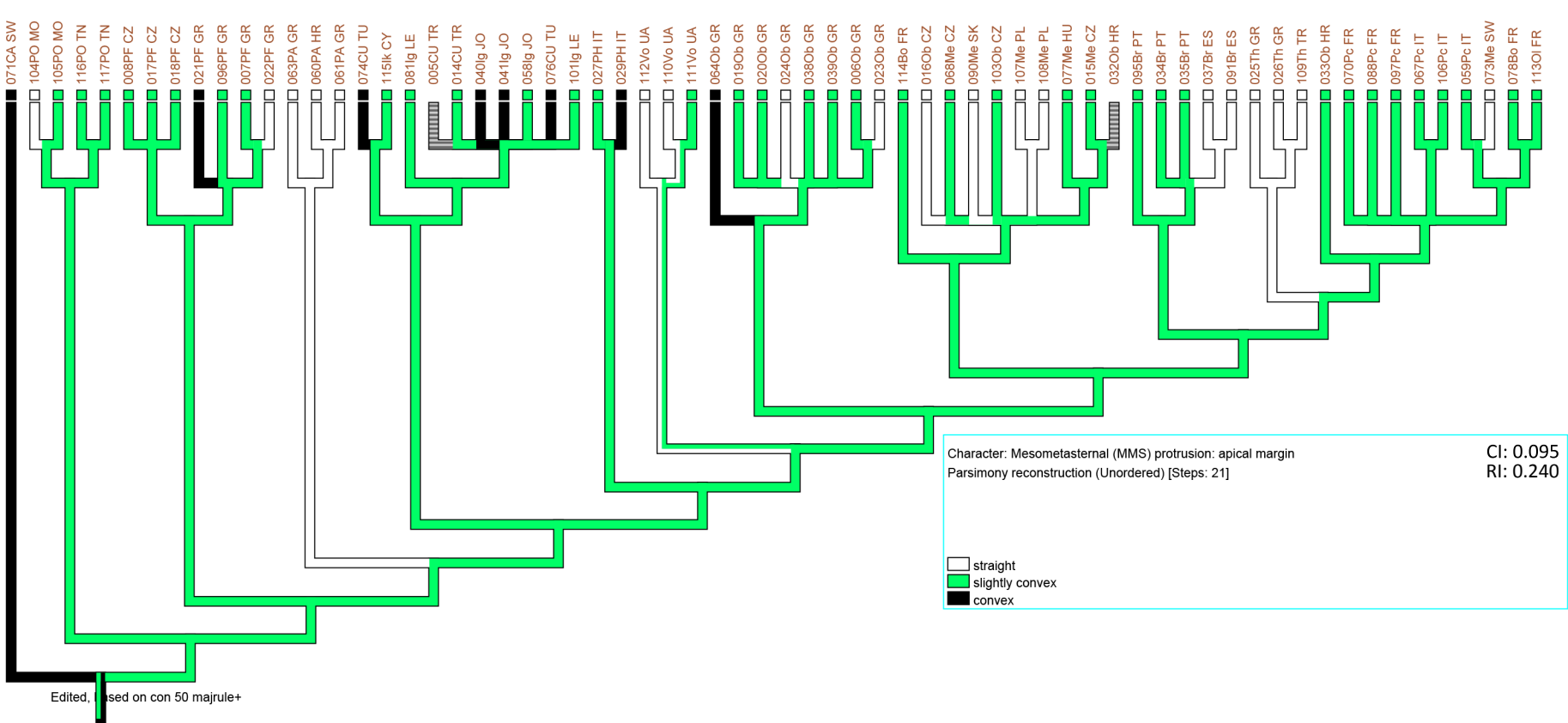

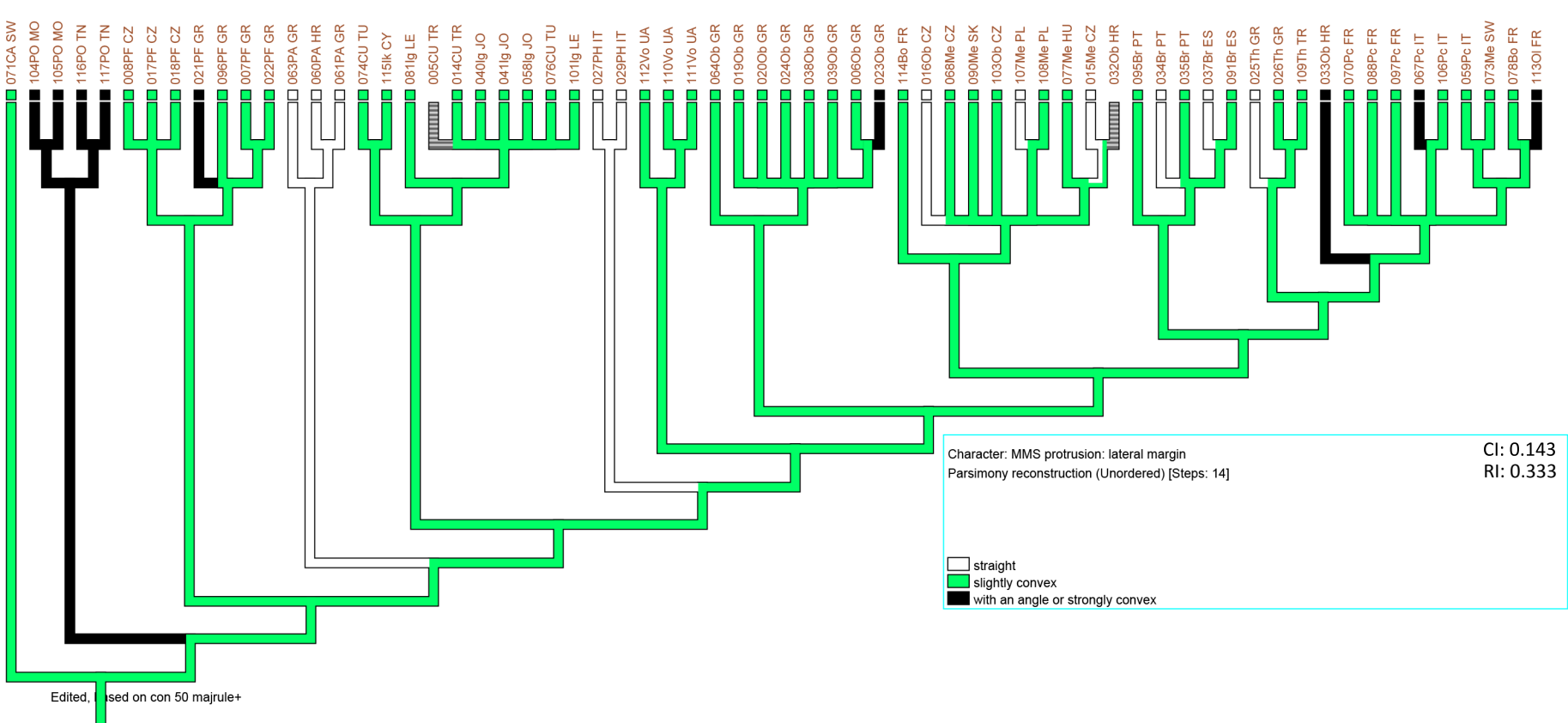

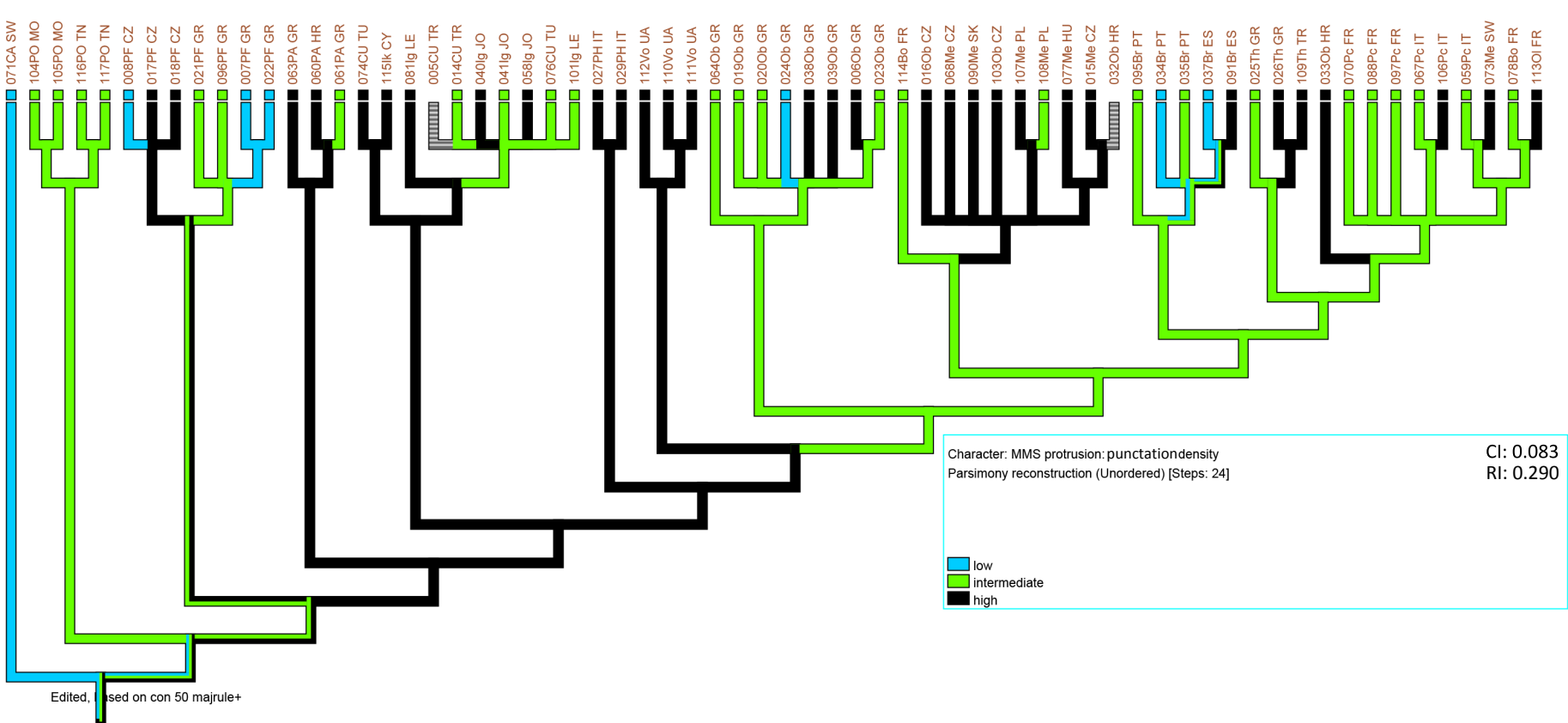

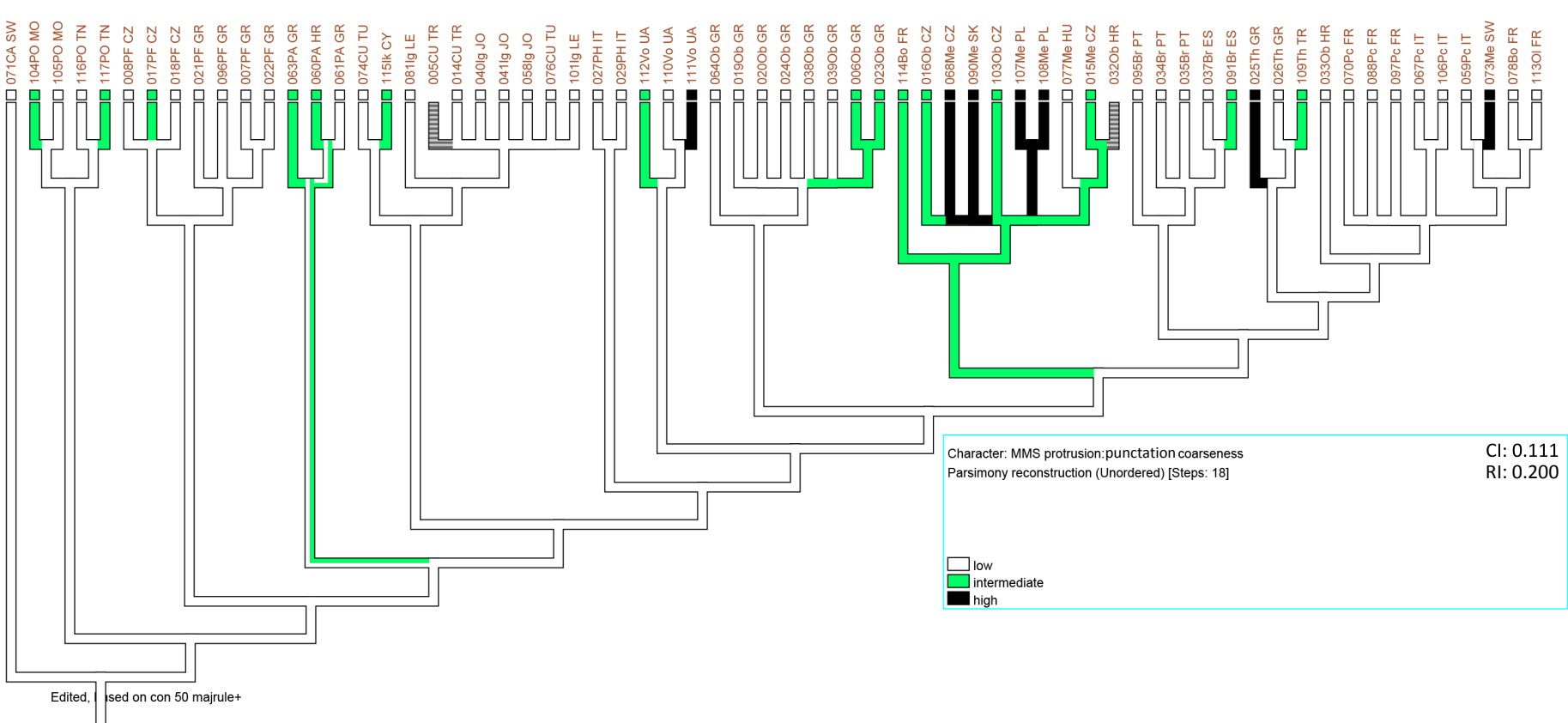

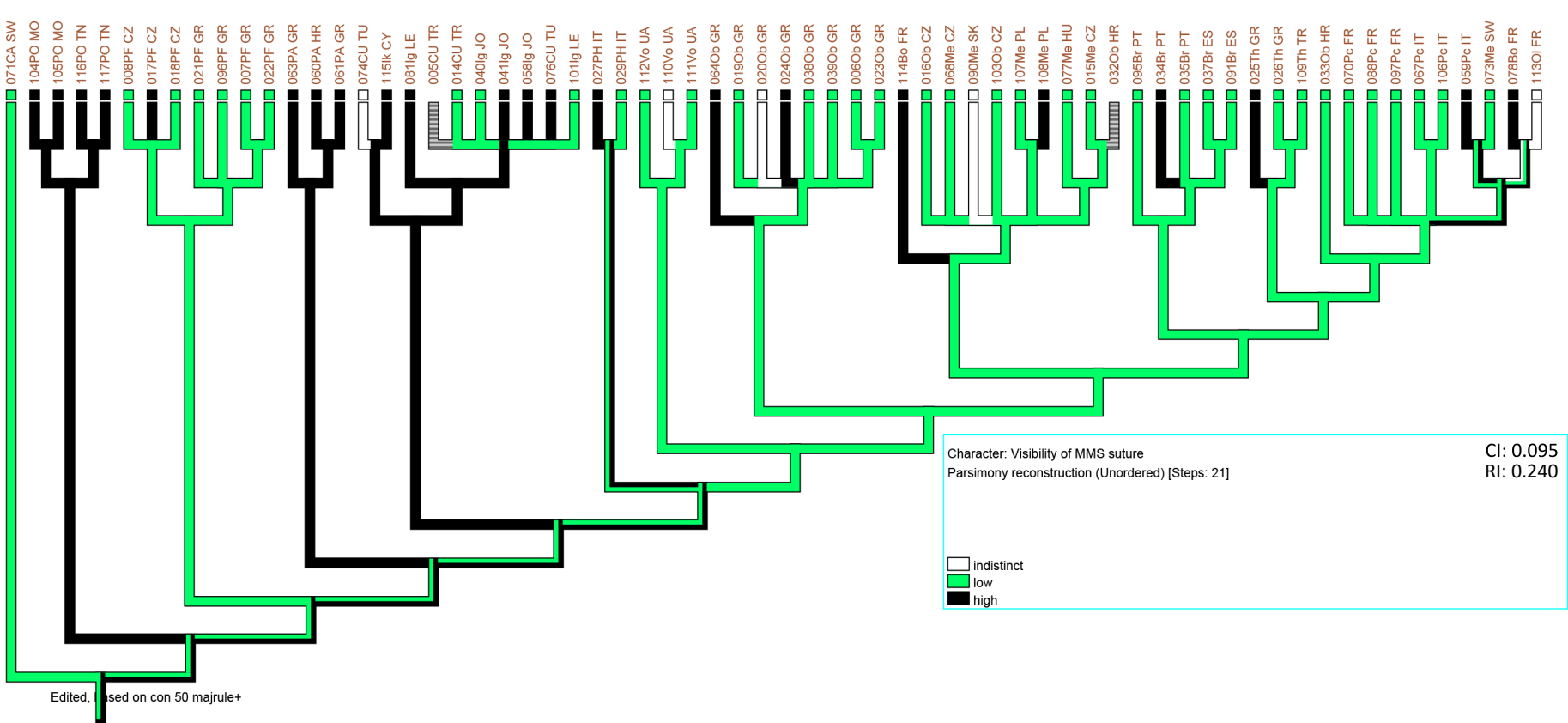

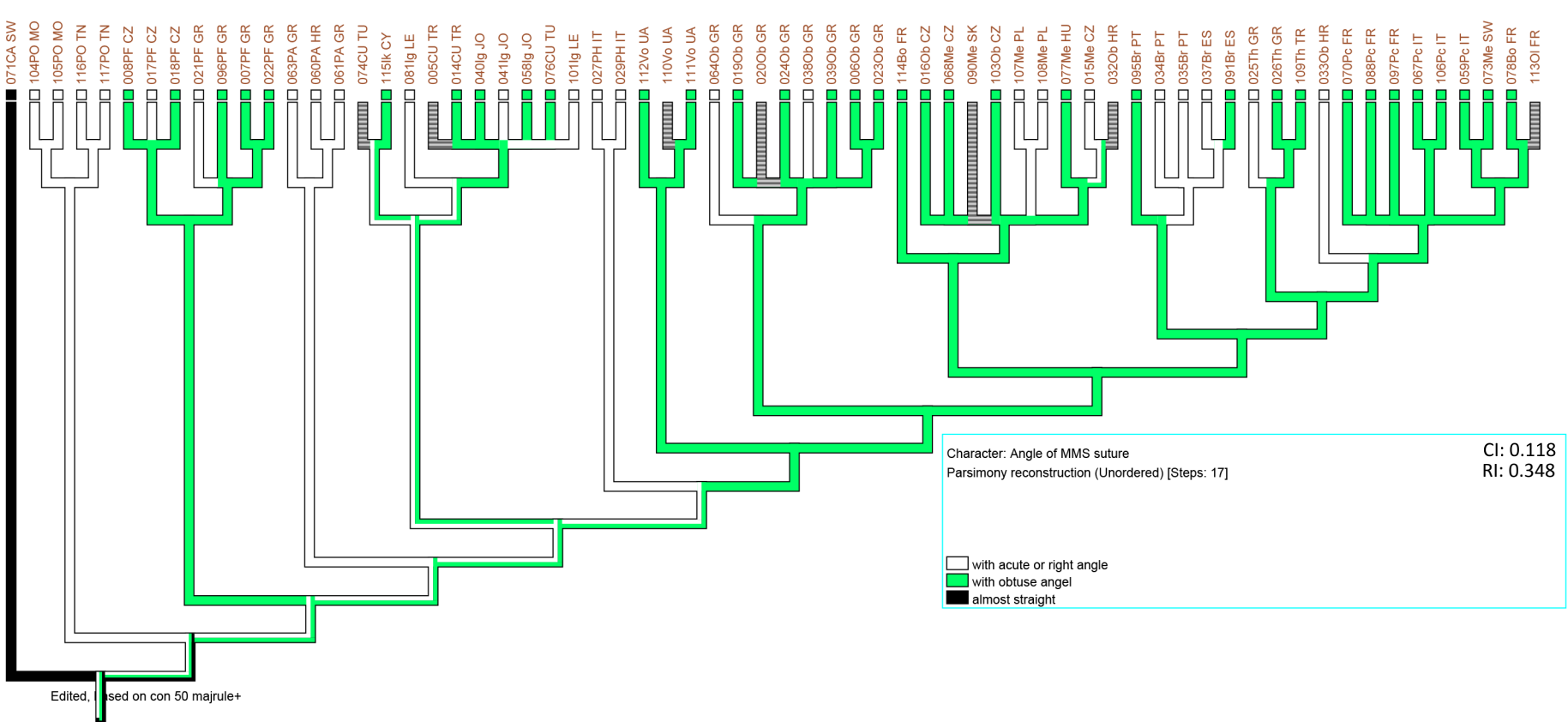

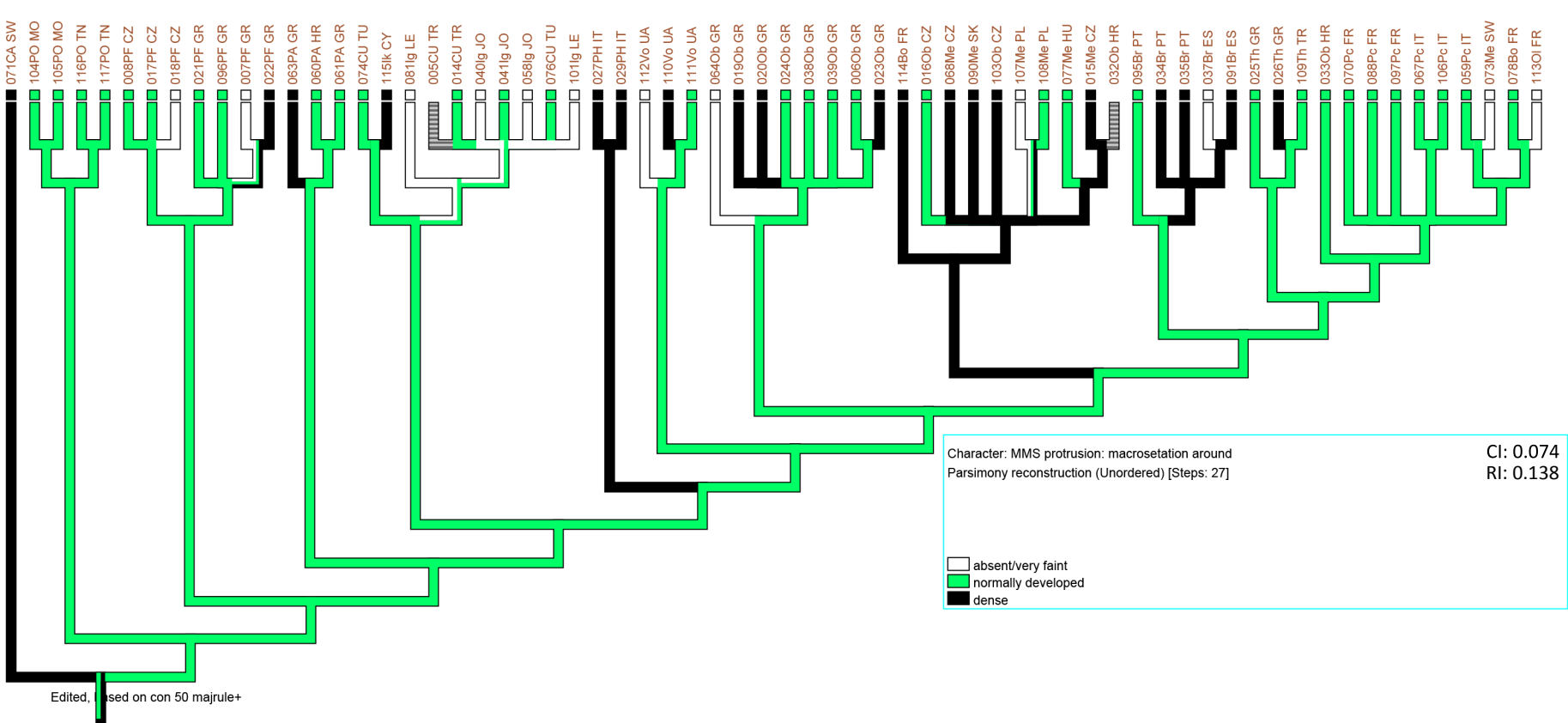

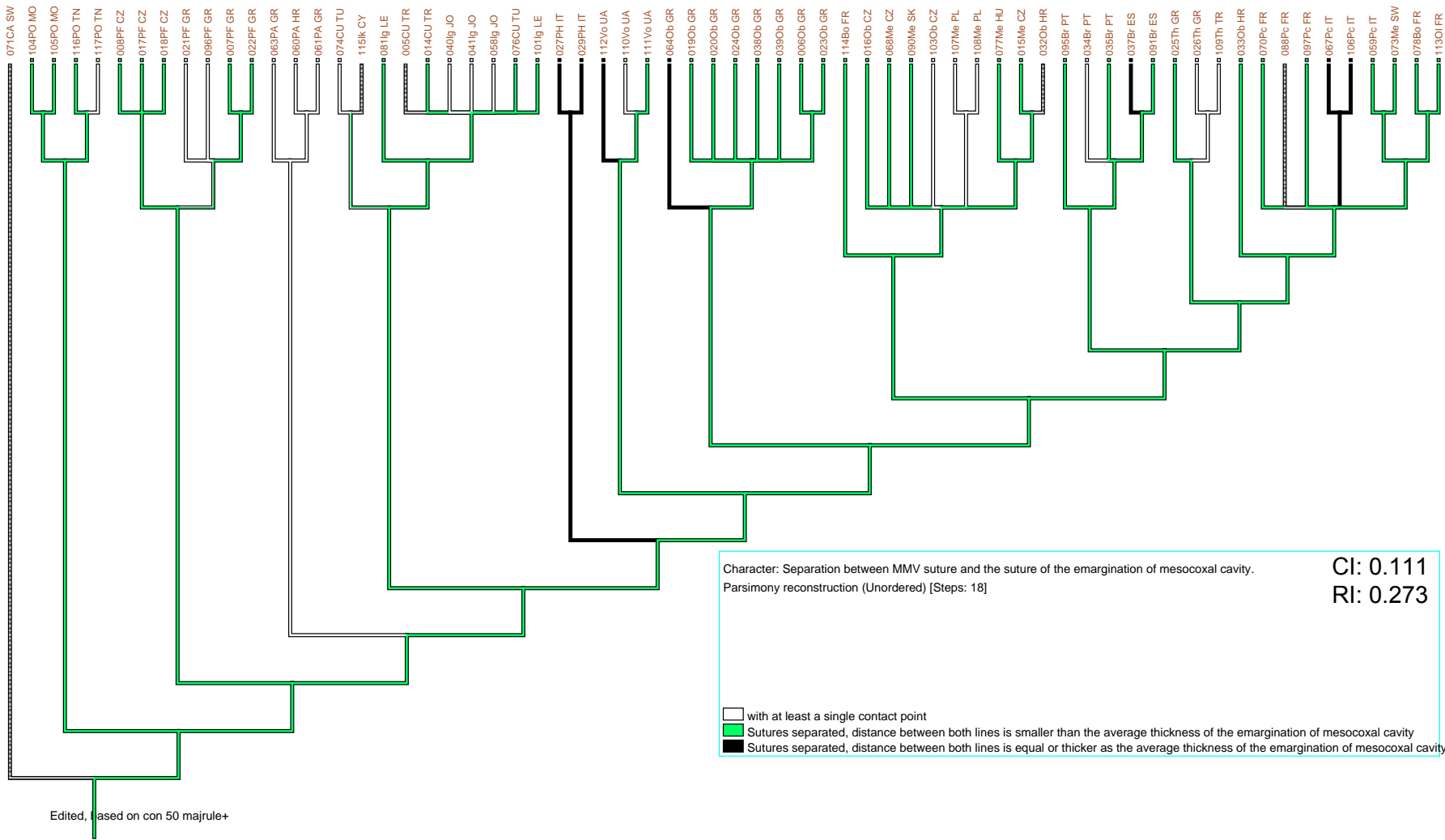

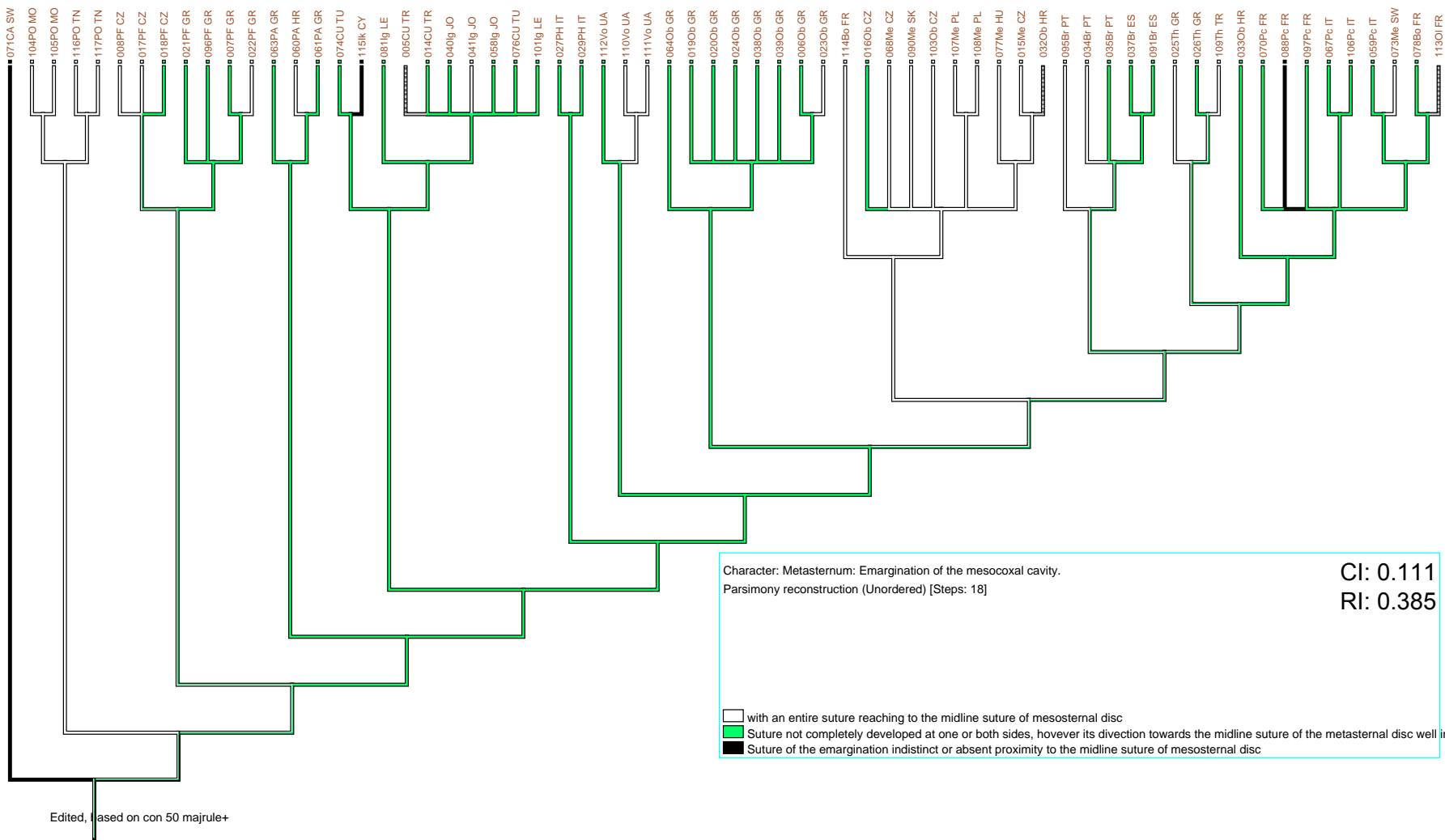

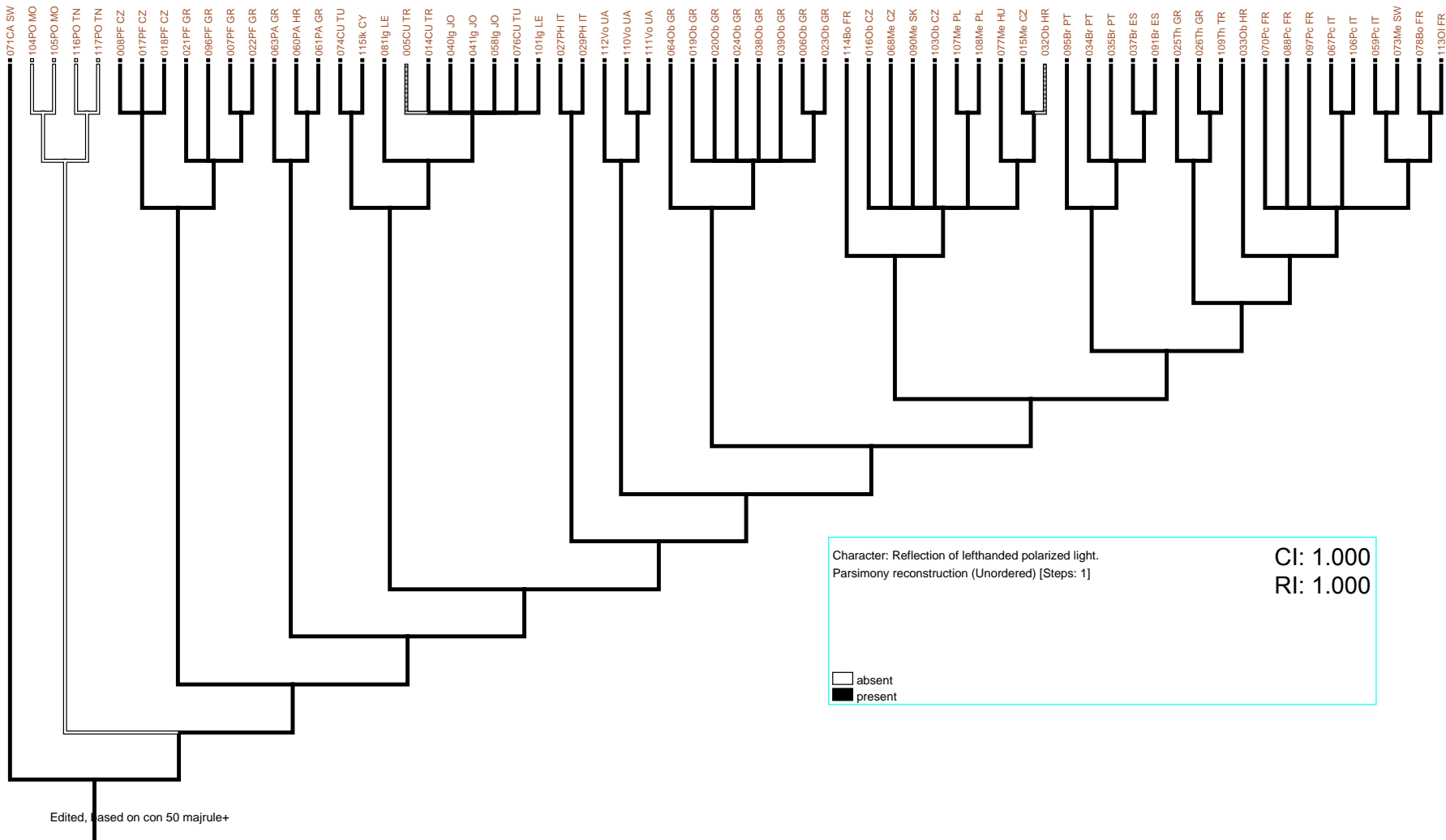

Supplement: S2 Fig — All 29 characters mapped separately on the phylogenetic tree from Fig 1. Legend as well as consistency (CI) and retention (RI) index values included in each tree. (PDF) [file pone.0192349.s007.pdf]

# Elytra

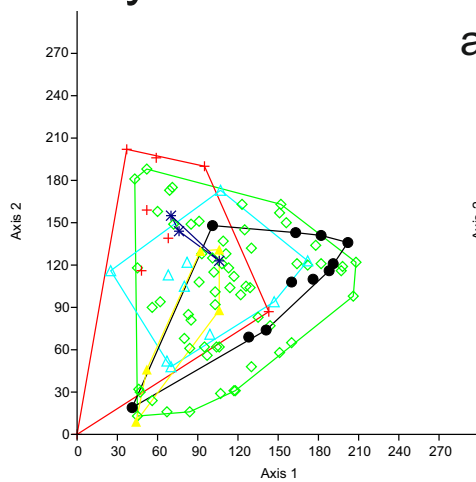

a

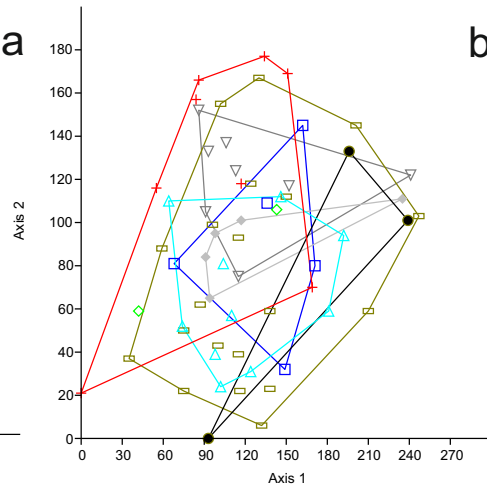

b

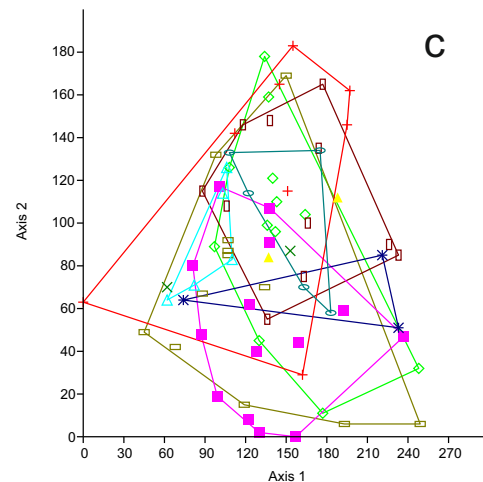

c

# Aedeagus

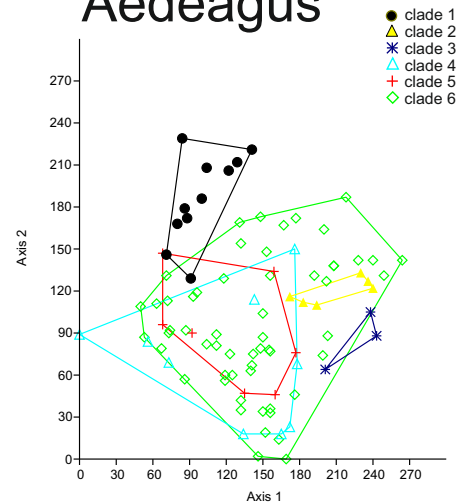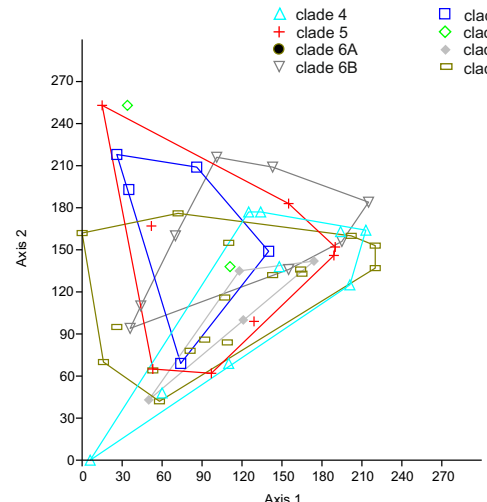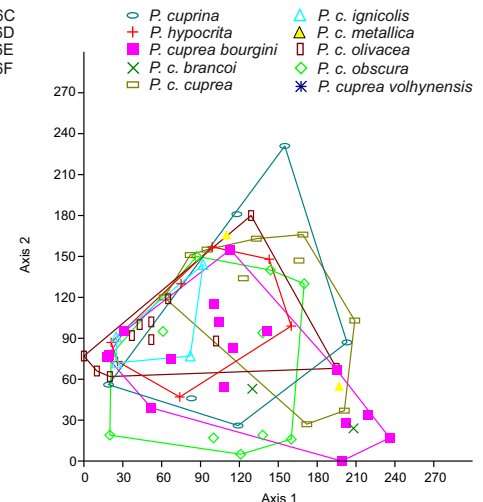

# MMV processus

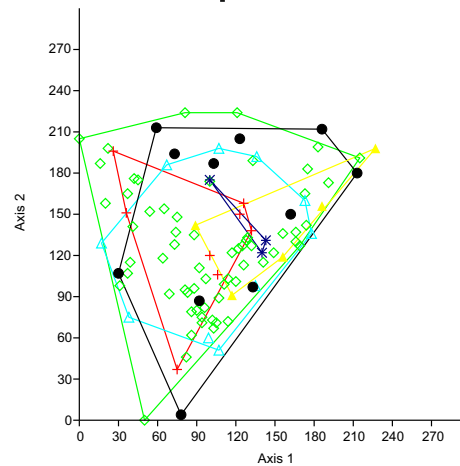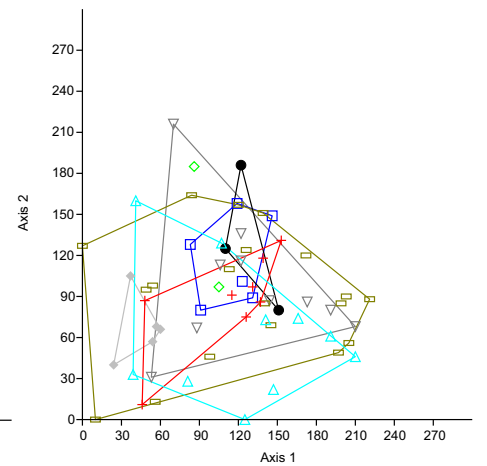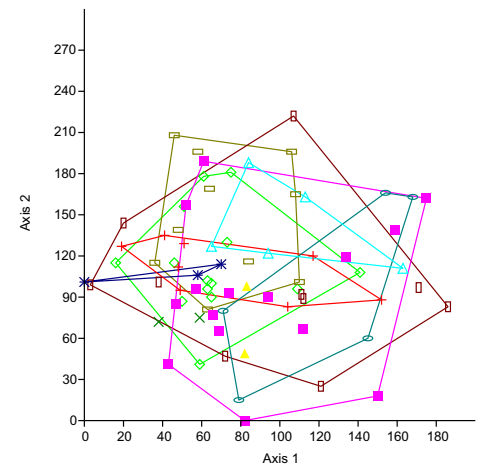

# Pronotum

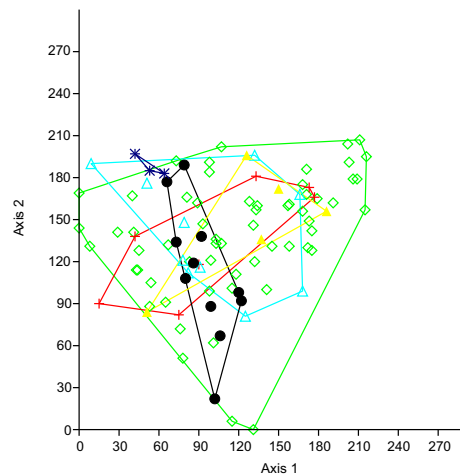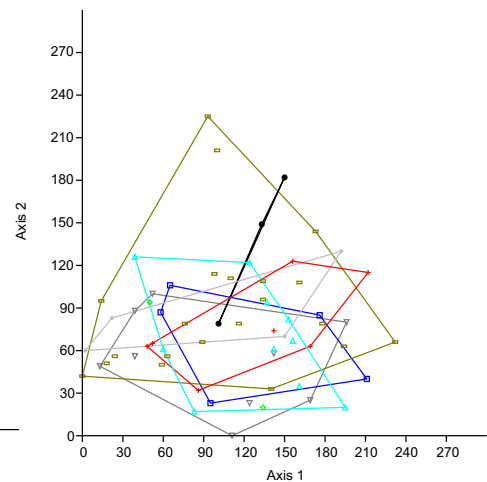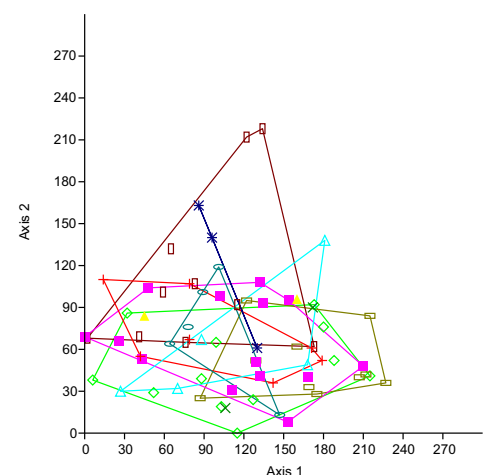

Supplement: S3 Fig — (PDF) [file pone.0192349.s008.pdf]
